# Supplementary material for: Mirvetuximab soravtansine plus pembrolizumab in recurrent folate receptor alpha-positive uterine serous carcinoma: a phase II trial
Source: Nat Commun. 2026 Mar 26;17:4449. doi: 10.1038/s41467-026-71102-x (PMC13183910; doi:10.1038/s41467-026-71102-x)
Supplement: Supplementary file 1 — Supplementary Information [file 41467_2026_71102_MOESM1_ESM.pdf]

Mirvetuximab Soravtansine plus Pembrolizumab in Recurrent Folate Receptor Alpha-Positive Uterine Serous Carcinoma: a phase II trial

Rebecca Porter[1*], Yinglu Zhou[1], Nebiyat Eskndir[1], Martin Hayes[1], Madeline Polak[1], Elizabeth K. Lee[1], Carolyn Krasner[1], Susana Campos[1], Alexi A. Wright[1], Joyce F. Liu[1], Elizabeth H. Stover[1], Hannah Sawyer[1], Niya Xiong[1], Kathleen L. Pfaff[1], Scott J. Rodig[2], Nabihah Tayob[1], Susan Zweizig[3], Veena John[4], Jennifer Veneris[1], Ursula A. Matulonis[1], Panagiotis A. Konstantinopoulos[1]

[1]Dana-Farber Cancer Institute, Boston, MA, USA

[2]Brigham & Women's Hospital, Boston, Massachusetts, USA

[3]University of Massachusetts Memorial Medical Center, Worcester, MA, USA

[4]Northwell Health Cancer Institute, New Hyde Park, NY, USA

***Corresponding Author**:
Rebecca L. Porter, M.D., Ph.D.
Division of Gynecologic Oncology
Dana-Farber Cancer Institute, Harvard Medical School
Yawkey Center for Cancer Care, YC-1437
450 Brookline Ave, Boston, MA, 02215
Email: rebecca_porter@dfci.harvard.edu

SUPPLEMENTARY INFORMATION

Supplemental Table 1. Associations between prior ICI exposure and clinical activity

	**Number of patients**	
**Best Response**	**ICI-exposed** N=8	**ICI-naïve** N=10
CR	0	1
PR	0	4
Unconfirmed PR	2	0
SD	1	3
PD	5	2
Confirmed ORR, % (95% CI)	0 (0, 37)	50 (19, 81)
Fisher's Exact Test	0.036	
Median PFS, months (95% CI)	1.23 (1.12, NR)	4.5 (1.15, NR)
Hazard Ratio (95% CI)<br>Log-rank Test p-value	0.16 (95% CI 0.04, 0.63)<br>p=0.003	

Supplemental Table 2. TRAEs of any grade occurring in >10% of patients

	Maximum Grade			
**Adverse Event***	**Grade 1**	**Grade 2**	**Grade 3**	**Any Grade**
Diarrhea	4 (22%)	3 (17%)	1 (6%)	8 (45%)
Fatigue	6 (33%)	2 (11%)	0 (0%)	8 (44%)
Aspartate aminotransferase increased	8 (44%)	0 (0%)	0 (0%)	8 (44%)
Blurred vision	5 (28%)	2 (11%)	0 (0%)	7 (39%)
Anemia	3 (17%)	2 (11%)	1 (6%)	6 (34%)
Peripheral sensory neuropathy	1 (6%)	3 (17%)	1 (6%)	5 (29%)
Anorexia	3 (17%)	2 (11%)	0 (0%)	5 (28%)
Platelet count decreased	2 (11%)	2 (11%)	1 (6%)	5 (28%)
Headache	3 (17%)	1 (6%)	0 (0%)	4 (23%)
Pneumonitis	2 (11%)	2 (11%)	0 (0%)	4 (22%)
Alanine aminotransferase increased	4 (22%)	0 (0%)	0 (0%)	4 (22%)
Creatinine increased	2 (11%)	1 (6%)	0 (0%)	3 (17%)
Nausea	3 (17%)	0 (0%)	0 (0%)	3 (17%)
Vomiting	3 (17%)	0 (0%)	0 (0%)	3 (17%)
Alkaline phosphatase increased	3 (17%)	0 (0%)	0 (0%)	3 (17%)
Pruritus	3 (17%)	0 (0%)	0 (0%)	3 (17%)
Keratitis	1 (6%)	1 (6%)	0 (0%)	2 (12%)
Fever	1 (6%)	1 (6%)	0 (0%)	2 (12%)
Lymphocyte count decreased	1 (6%)	1 (6%)	0 (0%)	2 (12%)
Rash maculo-papular	1 (6%)	1 (6%)	0 (0%)	2 (12%)
Neutrophil count decreased	1 (6%)	0 (0%)	1 (6%)	2 (12%)
Hypertension	1 (6%)	0 (0%)	1 (6%)	2 (12%)
Dry eye	2 (11%)	0 (0%)	0 (0%)	2 (11%)
Hypomagnesemia	2 (11%)	0 (0%)	0 (0%)	2 (11%)

* The denominator to all calculated percentages is 18, the number of patients who received at least one dose of study drug.

Supplemental Table 3. Associations between FOLR1 expression and clinical activity

	Number of patients	
**Best Response**	**FOLR1 50-74%**<br>N=9	**FOLR1 75-100%**<br>N=9
CR	0	1
PR	1	3
Unconfirmed PR	1	1
SD	3	1
PD	4	3
Confirmed ORR, % (95% CI)	11 (0, 48)	44 (14, 79)
Fisher's Exact Test	0.29	
Median PFS, months (95% CI)	2.63 (1.12, 4.5)	2.73 (1.18, 8.25)
Hazard Ratio (95% CI)<br>Log-rank Test p-value	0.66 (95% CI 0.23, 1.89)<br>0.4	

Supplemental Table 4. Conditions for multiplex immunofluorescence staining panel 1

Target	Antibody Vendor	Antibody clone	Catalog Number	Antigen Retrieval (buffer, min)	Primary Antibody dilution	Opal Fluorophore
CD8	Leica	4B11	MCA1817T	ER2, 40 min	1:1000	480
PD-L1	Cell Signaling Technology	E1L3N	13684	ER2, 10 min	1:300	520
FOXP3	Cell Signaling Technology	D2W8E	98377	ER2, 30 min	1:125	570
PD-1	Abcam	EPR4877(2)	ab137132	ER2, 30 min	1:300	620
CD163	Leica	10D6	10D6	ER1, 20 min	1:150	690
Cytokeratin	Dako	AE1/AE3	IR05361-2	ER1, 20 min	1:100	780

Supplemental Table 5. Conditions for multiplex immunofluorescence staining panel 2

Target	Antibody Vendor	Antibody clone	Catalog Number	Antigen Retrieval (buffer, min)	Primary Antibody dilution	Opal Fluorophore
CD8	Leica	4B11	MCA1817T	ER2, 40 min	1:1000	480
PD-L1	Cell Signaling Technology	E1L3N	13684	ER2, 10 min	1:300	520
FOLR1	Abcam	EPR20277	EPR20277	ER1, 30 min	1:1000	570
PD-1	Abcam	EPR4877(2)	ab137132	ER2, 30 min	1:300	620
CD163	Leica	10D6	10D6	ER1, 20 min	1:150	690
Cytokeratin	Dako	AE1/AE3	IR05361-2	ER1, 20 min	1:100	780

Supplemental Figure 1.

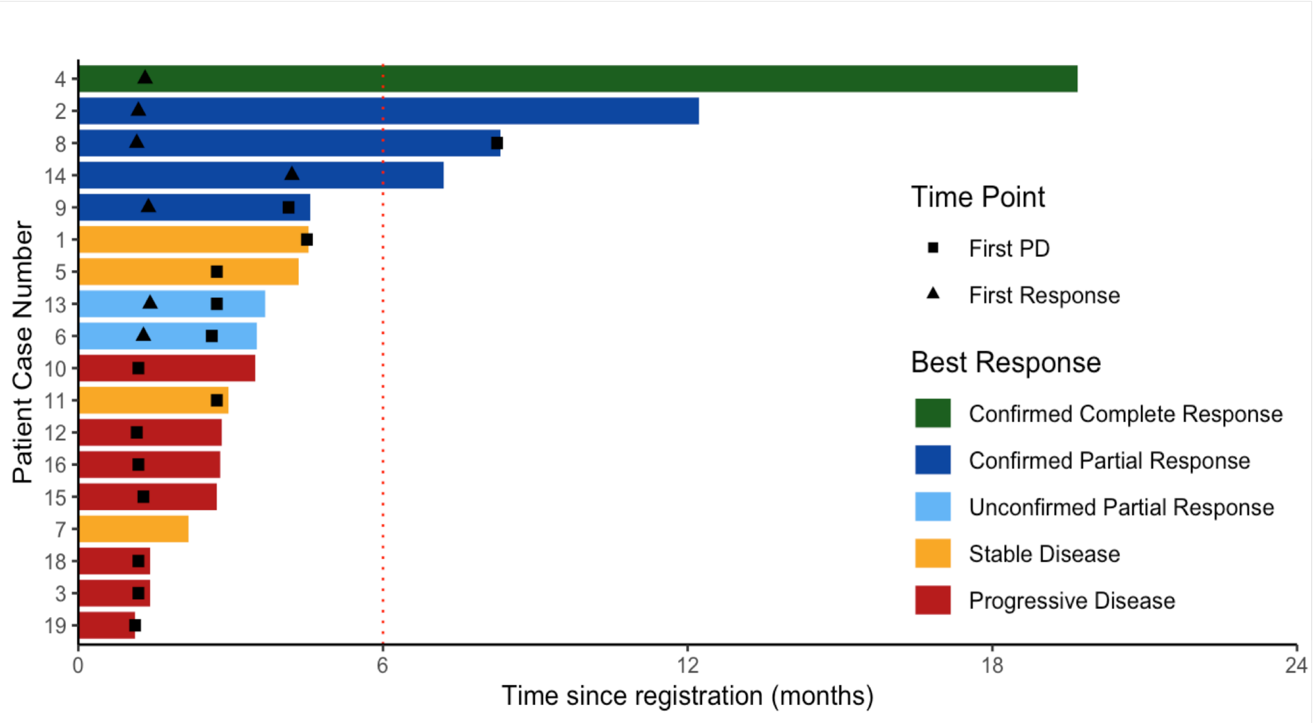

Supplemental Figure 1. Duration of treatment by subject. Swimmer plot of 18 patients treated on trial. Note: No patients remain on study treatment. PD, progressive disease.

Supplemental Figure 2.

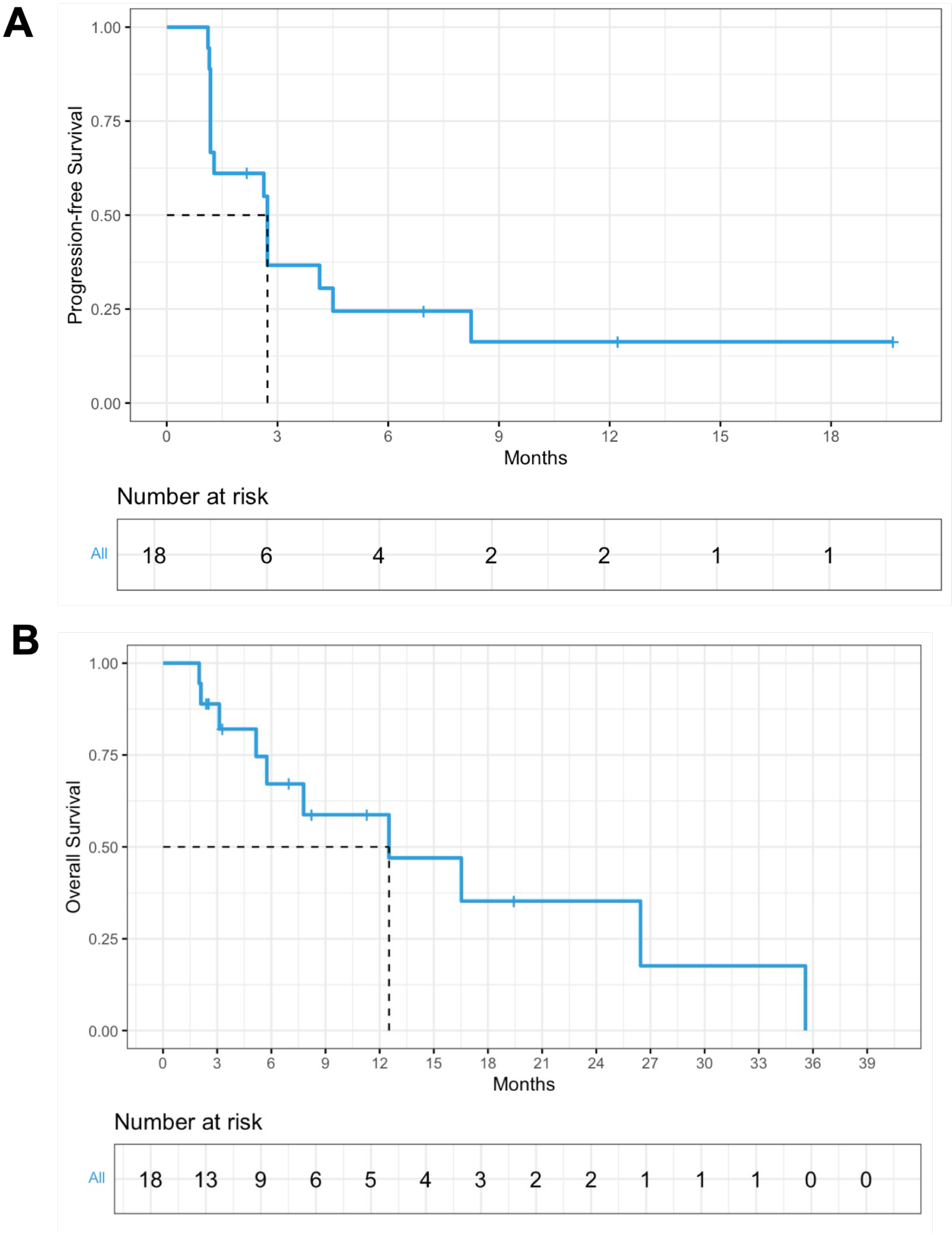

Supplemental Figure 2. Kaplan-Meier analysis of progression free (A) and overall survival (B) for all 18 treated patients.

Supplemental Figure 3.

A

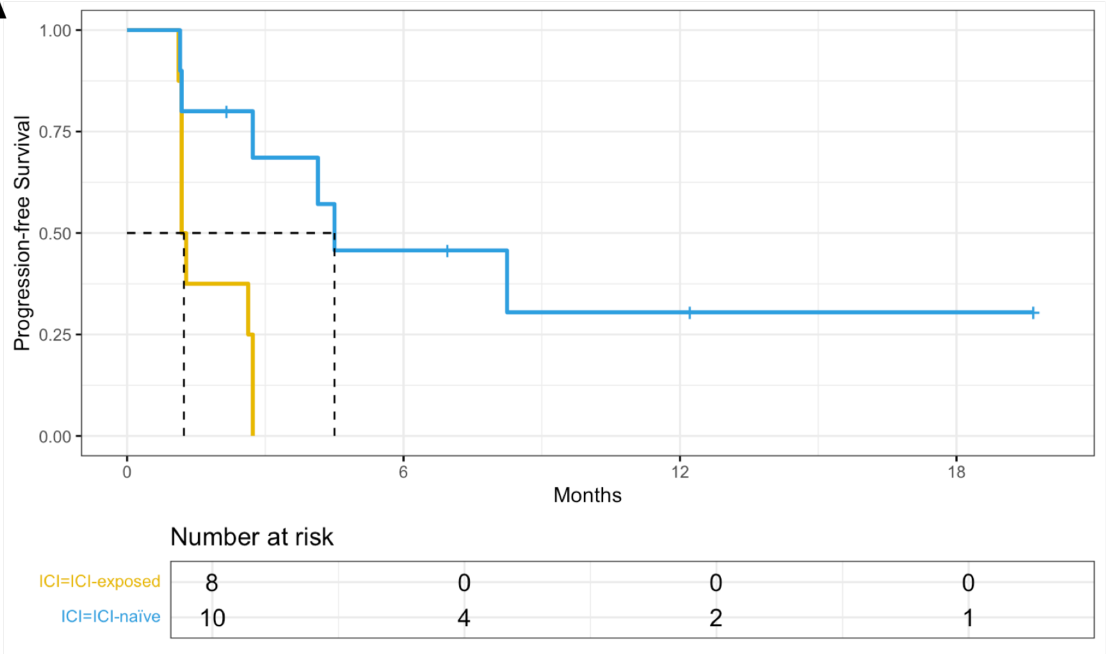

B

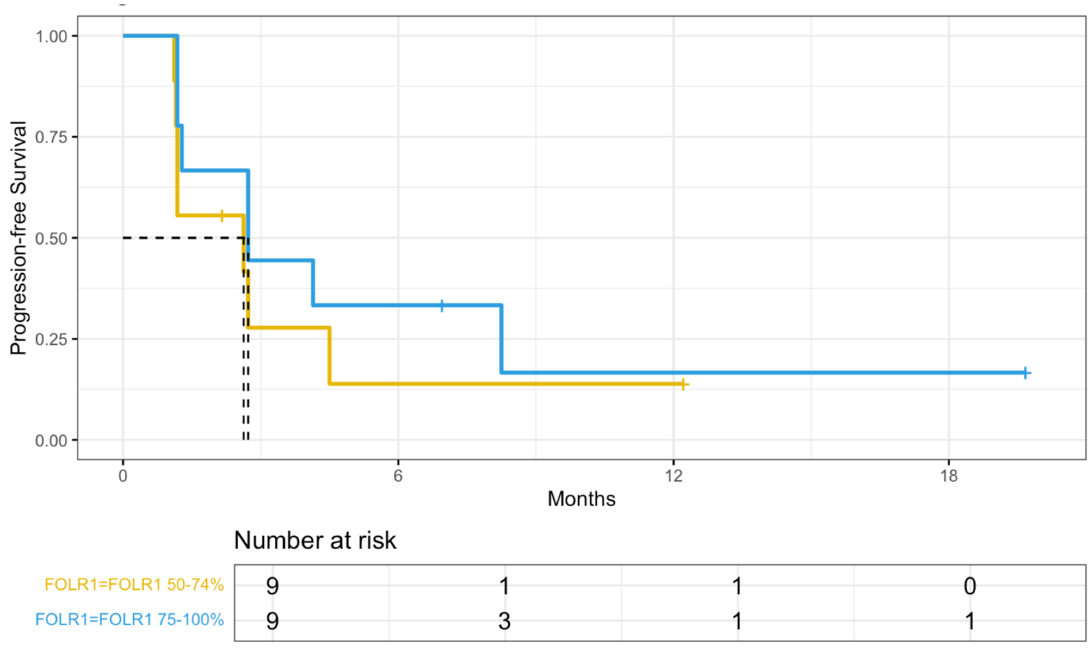

Supplemental Figure 3. Kaplan-Meier analyses of progression free survival for all 18 patients based on (A) prior IO exposure history and (B) FOLR1 expression level.

Supplemental Figure 4.

A

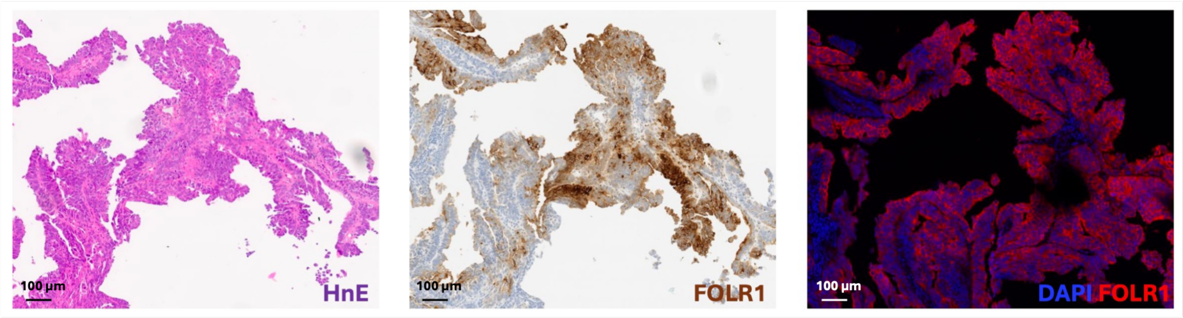

B

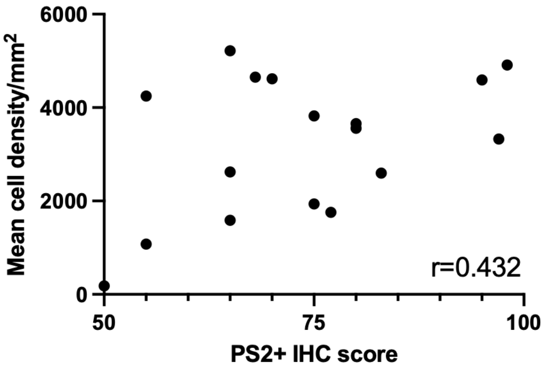

Supplemental Figure 4. Correlation of FOLR1 IF with IHC. A. Representative hematoxylin and eosin (H&E) (*left*), FOLR1 IHC (*middle*) and FOLR1 IF (*right*) images for the same patient. Images are representative of the 17 patients whose tumors underwent immunoprofiling. **B.** Scatter plot illustrating the relationship between FOLR1 IF and FOLR1 IHC in 17 patients with immunoprofiling. Each point represents a single tumor. A positive association using Pearson correlation is indicated with r=0.432, p=0.0836.

Supplemental Figure 5.

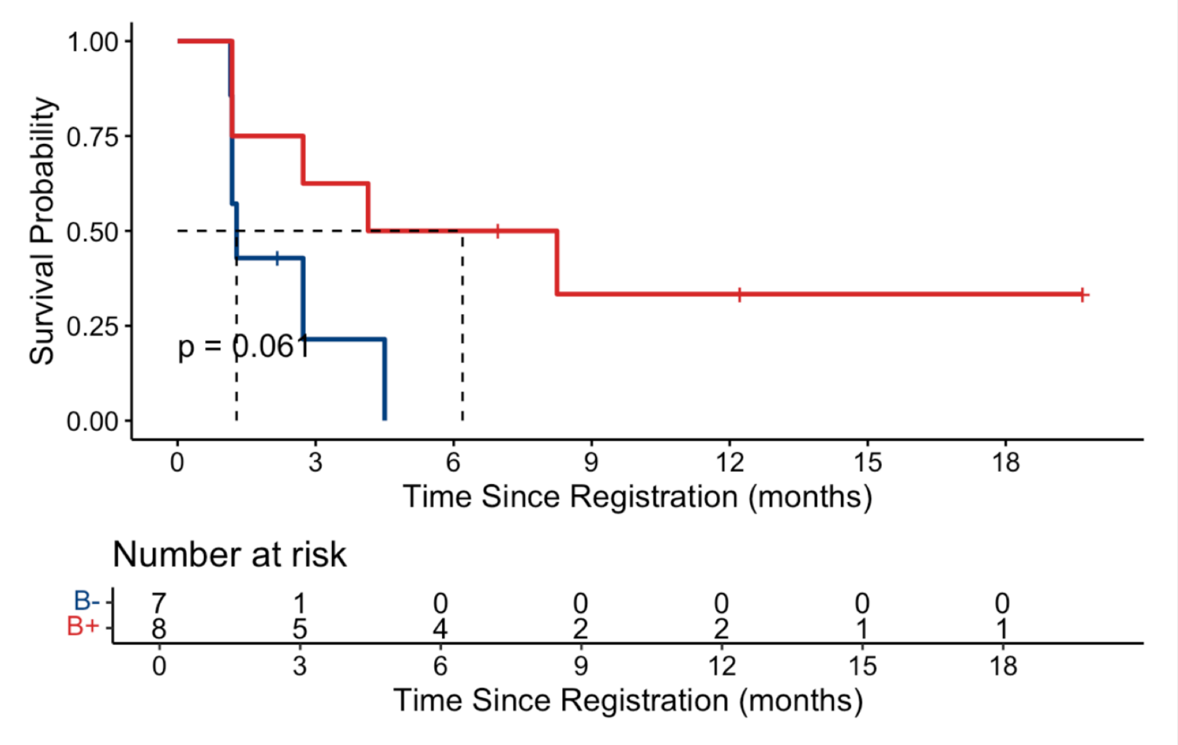

Supplemental Figure 5. Kaplan-Meier analysis of progression free survival by composite biomarker score. Kaplan Meier analysis of PFS for all 18 patients based on Biomarker score. Tumors with a B-score of 2 points or higher are B+; tumors with B-score of 0 or 1 are B-.

NCI Protocol #: N/A

DF/HCC Protocol #: 18-602

TITLE: A Phase 2, two-stage, study of mirvetuximab soravtansine (IMGN853) in combination with pembrolizumab in patients with microsatellite stable (MSS) recurrent or persistent endometrial cancer (EC)

Coordinating Center: Dana-Farber/Harvard Cancer Center (DF/HCC)

Principal Investigator (PI): Rebecca Porter, MD, PhD
Dana-Farber Cancer Institute
450 Brookline Ave
Boston, MA 02215
Telephone: 617-632-2334
Fax: 617-582-7921
Rebecca_Porter@dfci.harvard.edu

Statistician:
Nabihah Tayob, PhD
Dana-Farber Cancer Institute
450 Brookline Ave
Boston, MA 02215
Telephone: 857-215-0006
ntayob@ds.dfci.harvard.edu

Co-PI:
Panagiotis Konstantinopoulos, MD, PhD
Dana-Farber Cancer Institute
450 Brookline Ave
Boston, MA 02215
Telephone: 617-632-5269
Fax: 617-582-7921
Panagiotis_Konstantinopoulos@dfci.harvard.edu

Responsible Research Nurse:
Sarah Hindenach, RN
Dana-Farber Cancer Institute
450 Brookline Ave.
Boston, MA 02215
Telephone: 617-632-3472
Fax: 617-582-7921
Sarah_Hindenach@dfci.harvard.edu

Project Manager:
Martin Hayes
Dana-Farber Cancer Institute
450 Brookline Ave.
Boston, MA 02215
Telephone: 857-215-2911
Fax: 617-582-7921
Martin_Hayes@dfci.harvard.edu

NCI-Supplied Agent(s): N/A
Other Agent(s): Mirvetuximab soravtansine (IMGN853, ImmunoGen, Inc.); Pembrolizumab (Keytruda, Merck)

IND #: 140407
IND Sponsor: Panagiotis Konstantinopoulos, MD, PhD

Protocol Type / Version # / Version Date: Amendment 14 / Version 15 / 11 December 2023

SCHEMA
1 cycle = 21 days

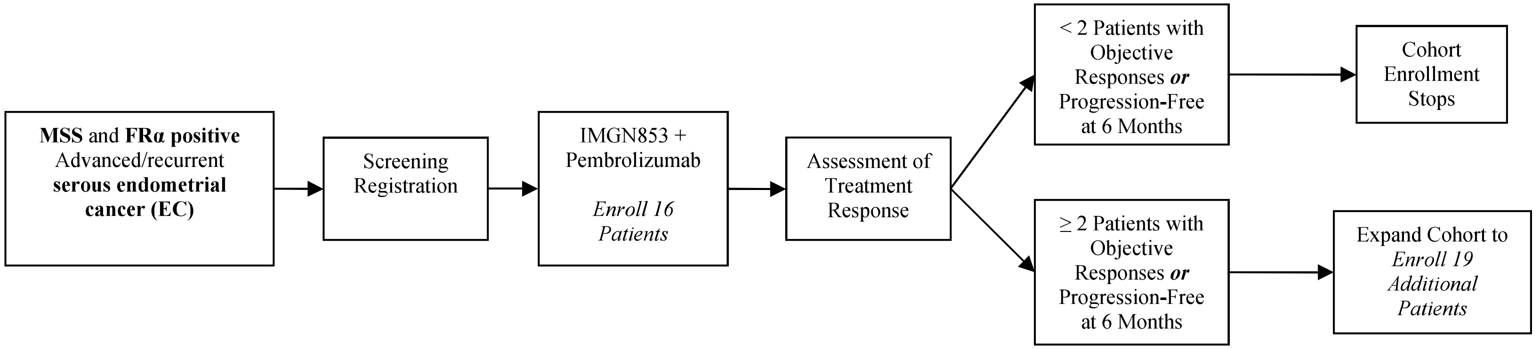

TABLE OF CONTENTS

SCHEMA 2

OBJECTIVES 6

1.1 Study Design 6
1.2 Primary Objectives 6
1.3 Secondary Objectives 6
1.4 Exploratory Objectives 6

BACKGROUND 7

2.1 Endometrial Cancer 7
2.2 Investigational agents – Mirvetuximab Soravtansine (IMGN853) 8
2.3 Rationale 12
2.4 Correlative Studies Background 14

PARTICIPANT SELECTION 15

3.1 Eligibility Criteria 15
3.2 Exclusion Criteria 17
3.3 Inclusion of Women and Minorities 19

REGISTRATION PROCEDURES 19

4.1 General Guidelines for DF/HCC Institutions 19
4.2 Registration Process for DF/HCC Institutions 19
4.3 General Guidelines for Other Investigative Sites 19
4.4 Registration Process for Other Investigative Sites 20

TREATMENT PLAN 20

5.1 Treatment Regimen 20
5.2 Pre-Treatment Criteria 21
5.3 Agent Administration 21
5.4 General Concomitant Medication and Supportive Care Guidelines 26
5.5 Criteria for Taking a Participant Off Protocol Therapy 29
5.6 Duration of Follow Up 30
5.7 Criteria for Taking a Participant Off Study 30

DOSING DELAYS/DOSE MODIFICATIONS 31

6.1 Mirvetuximab Soravtansine (IMGN853) 31
6.2 Pembrolizumab 35
6.3 Re-treatment Criteria 39

ADVERSE EVENTS: LIST AND REPORTING REQUIREMENTS 40

7.1 Expected Toxicities 40
7.2 Adverse Event Characteristics 41
7.3 Serious Adverse Events 41
7.4 Adverse Event Reporting to Overall PI 42
7.5 Reporting Period 43

7.6 Adverse Event Reporting to ImmunoGen....43
7.7 Adverse Event Reporting to Merck....44
7.8 Reporting to the Food and Drug Administration (FDA)....46
7.9 Reporting to Hospital Risk Management....47
7.10 Routine Adverse Event Reporting....47

PHARMACEUTICAL INFORMATION....47
8.1 Mirvetuximab Soravtansine (IMGN 853)....47
8.2 Pembrolizumab....53

BIOMARKER, CORRELATIVE, AND SPECIAL STUDIES....57
9.1 Biomarker Studies....57
9.2 Laboratory Correlative Studies....58

STUDY CALENDAR....59

MEASUREMENT OF EFFECT....61
11.1 Response Evaluation Criteria in Solid Tumors (RECIST) Version 1.1 Guidelines....61
11.2 Immune-related Response Criteria Derived from RECIST 1.1 (irRECIST)....64
11.3 Methods for Evaluation of Disease....66

DATA REPORTING / REGULATORY REQUIREMENTS....68
12.1 Data Reporting....68
12.2 Data Safety Monitoring....68
12.2 Multi-Center Guidelines....68

STATISTICAL CONSIDERATIONS....69
13.1 Study Design/Endpoints....69
13.2 Sample Size, Accrual Rate and Study Duration....70
13.3 Stratification Factors....71
13.4 Interim Monitoring Plan....71
13.5 Analysis of Primary Endpoints....71
13.6 Analysis of Secondary Endpoints....71
13.7 Reporting and Exclusions....72

PUBLICATION PLAN....72

REFERENCES....74

APPENDIX AECOG Performance Status....77

APPENDIX B: Dana-Farber/Harvard Cancer Center Multi-Center Data and Safety Monitoring Plan 78

1. INTRODUCTION....80
1.1. Purpose....80

2. GENERAL ROLES AND RESPONSIBILITIES 80
2.1. Coordinating Center 80
2.2. External Site 81

3. DF/HCC REQUIREMENTS FOR MULTI-CENTER PROTOCOLS 81
3.1. Protocol Revisions and Closures 81
3.2. Informed Consent Requirements 82
3.3. IRB Re-Approval 82
3.4. DF/HCC Multi-Center Protocol Confidentiality 82
3.5. Participant Registration 83
3.6. Initiation of Therapy 83
3.7. Eligibility Exceptions 83
3.8. Data Management 83
3.9. Protocol Reporting Requirements 84

4. MONITORING: QUALITY CONTROL 84
4.1. Ongoing Monitoring of Protocol Compliance 84
4.2. Monitoring Reports 85
4.3. Accrual Monitoring 85

5. AUDITING: QUALITY ASSURANCE 85
5.1. DF/HCC Internal Audits 85
5.2. Audit Notifications 85
5.3. Audit Reports 85
5.4. External Site Performance 86

APPENDIX C CTCAE Version 5.0 Grading for selected Adverse Events 87

APPENDIX D Schema for administration of study-required eye drops 89

OBJECTIVES

1.1 Study Design

An open label, two-stage, non-randomized phase 2 trial examining mirvetuximab soravtansine (IMGN853) and pembrolizumab in participants with advanced or recurrent serous endometrial cancer (EC). The combination will be tested in patients with microsatellite stable (MSS) and FRα positive tumors.

Enrollment will follow a standard Simon two-stage optimal design [1] for a single cohort to inform whether the combination has significant clinical activity worthy of further evaluation. Statistical considerations are developed for co-primary objectives to evaluate the objective response rate (ORR) and rate of progression-free survival at 6 months (PFS6), with a two-stage design that allows for early stopping for futility.

Target enrollment is a maximum of 35 patients.

1.2 Primary Objectives

To assess the activity of mirvetuximab soravtansine and pembrolizumab in advanced or recurrent EC as determined by the frequency of patients who are alive and progression-free for a minimum of 6 months (PFS6) after initiating therapy, or who have objective tumor responses by RECIST 1.1 criteria.

1.3 Secondary Objectives

1. To determine the duration of progression-free survival (PFS), overall survival (OS) and duration of response (DoR)
2. To determine the nature and degree of toxicity of protocol therapy as classified using the Common Terminology Criteria for Adverse Events (CTCAE) version 5.0
3. To determine Immune-related objective response rate (irORR)
4. To determine Immune-related progression-free survival (irPFS) rate defined as time from treatment initiation to death or to immune-related progression of disease (irPD) as defined in Sections 11.2 and 13.6

1.4 Exploratory Objectives

Correlative studies will be performed in the Immune Oncology and DNA Repair Centers at DFCI and Ventana Medical Systems. The aims are to:

1. To assess CD3+ tumor infiltrating lymphocytes (TILs) and circulating lymphocytes,CD8+ TILs, CD8+/CD4+FOXP3+ TIL ratio, CD137+CD8+ TILs, CD137+CD8+/CD4+FOXP3+ TIL ratio and correlate with response.
2. To assess myeloid, stromal and other immunoactive cell types from blood, tissue and fluid samples and correlate with response.
3. To assess the expression pre-, during, and at time of progression of immune checkpoints including TIM-3, LAG-3, CTLA-4, PD-L2, PD-L1, PD-1, IDO within tumor and/or

blood samples and correlate with response.

4. To perform whole exome sequencing (WES) to assess for specific DNA gene repair mutations and neoantigens as well as for single nucleotide polymorphisms (SNPs) in immunologically relevant genes and correlate with response

Additional exploratory analyses will include a comparison of the activity of mirvetuximab soravtansine and pembrolizumab in patients with and without prior exposure to IO therapy as determined by the frequency of patients who are alive and progression-free for a minimum of 6 months (PFS6) after initiating therapy, or who have objective tumor responses by RECIST 1.1 criteria in each category. The secondary objectives as defined in Section 1.3 will also be evaluated in each category.

BACKGROUND

2.1 Endometrial Cancer

Endometrial cancer (EC) is the most common gynecologic malignancy in the United States with approximately 60,000 new cases and 10,000 deaths anticipated in 2017 [2]. Approximately two thirds of women with endometrial cancer present with early-stage, uterus-confined disease. This is typically treated surgically with or without radiotherapy with excellent outcomes. However, women with recurrent disease or metastatic disease are incurable with a five-year survival rate of 17% and limited treatment options [3].

There have been several randomized studies performed to ascertain the optimal therapy in patients with recurrent or metastatic disease. These studies have focused on three active chemotherapy agents identified to have significant activity as monotherapy in phase 2 trials: doxorubicin, platinum agents, and paclitaxel. In GOG-0107, 281 women were randomized to doxorubicin alone versus doxorubicin plus cisplatin (AP). There was a statistically significant advantage to combination therapy with regard to response rate (RR) (25% versus 42%; p=0.004) and PFS (3.8 vs 5.7 months; HR 0.74, 95% CI 0.58, 0.94; p=0.14), although no difference in OS was observed [4]. Phase 2 data published by the GOG in 1996 demonstrated that paclitaxel had significant single agent activity with a response rate of 36% in advanced or recurrent endometrial cancer [5]. Thus 317 patients were randomized to paclitaxel and doxorubicin or the standard arm of AP in GOG-0163 [6]. This trial failed to demonstrate a significant difference in RR, PFS, or OS between the two arms, and AP remained the standard of care.

However, since both platinum and paclitaxel had demonstrated high single agent activity, there was a strong interest in including paclitaxel and cisplatin in a front-line regimen for advanced and recurrent endometrial cancer. Subsequently, GOG-0177 randomized 263 patients to AP versus doxorubicin, paclitaxel, and cisplatin (TAP): doxorubicin and cisplatin on day 1, followed by paclitaxel on day 2. TAP was superior to AP in terms of ORR (57% vs 34%; p<0.01), median PFS (8.3 vs 5.3 months; p<0.01), and OS with a median of 15.3 (TAP) versus 12.3 months (AP) (p=0.037) [7]. This improved efficacy came at the cost of increased toxicity. In GOG-0209 TAP was compared in a randomized fashion to paclitaxel and carboplatin (CT) in an attempt to address the issue of toxicity; CT was not inferior to TAP in terms of PFS and OS. At the reported interim analysis, the median PFS was 14 months in both arms (HR, 1.03), while median OS was

32 months vs. 38 months in patients treated with CT vs. TAP (not significant, HR, 1.01). Additionally, CT was better tolerated than TAP.

Patients with a relapse not amenable to local therapy currently receive treatment with a CT combination as a first-line alternative to the more toxic triplets [8]. In the second line setting, there is currently no single standard of care – various cytotoxic agents have been used including paclitaxel, docetaxel, liposomal doxorubicin, topotecan, oxaliplatin, ixabepilone, pemetrexed, gemcitabine with response rates of 4-27%, with only paclitaxel having a response rate >20% [9, 10]. Other agents with modest activity in the second line setting include hormonal (progestins, antiestrogens, estrogen modulators), and targeted therapies including human epidermal growth factor receptor 2 (Her-2), vascular endothelial growth factor (VEGF) inhibitors, and PI3K/mTOR inhibitors, among others. The treatment of women with advanced, recurrent, or metastatic EC represents an important unmet need.

2.2 Investigational agents – Mirvetuximab Soravtansine (IMGN853)

Investigational Agent Rationale

Mirvetuximab soravtansine (IMGN853) is a specific, targeted antibody drug conjugate (ADC) that binds with high affinity to folate receptor alpha (FRα), a glycophosphatidylinositol-linked protein which is highly expressed on the surface of solid tumors, particularly epithelial ovarian cancer (EOC), endometrial cancer, and non-small cell lung carcinoma (NSCLC). Mirvetuximab soravtansine consists of a humanized anti-FRα monoclonal antibody attached via a disulfide containing linker to the cytotoxic maytansinoid, DM4. Once released within the target cell, DM4 acts as an anti-mitotic agent that inhibits tubulin polymerization and microtubule assembly, resulting in cell cycle arrest and apoptosis.

Rationale for Dose Selection

Dose selection for this study is based on the recommended Phase 2 dose (RP2D) of the combination of IMNG853 and pembrolizumab, as determined from the Phase Ib Study 0402.

Clinical Safety Data Related to Dose

As of 22 October 2020, 737 patients had received at least 1 dose of MIRV in 1 of 7 studies. Two hundred and six patients were dosed in Study IMGN853-0401, a first-in-human, Phase 1, single-agent, dose escalation and dose expansion study; 264 patients have been dosed in the ongoing Study IMGN853-0402, Phase 1b/2, dose escalation, dose expansion combination study with BEV, Carbo, Pembro, PLD, or BEV + Carbo (data cut-off 16 October 2020); 243 patients were dosed in the pivotal stage of Study IMGN853-0403, a Phase 3, single-agent, pivotal study; 10 patients have been dosed with MIRV monotherapy in the ongoing Study IMGN853-0416, Phase 3, safety and efficacy study of MIRV versus IC Chemo (Pac, PLD, or topotecan) (data cut-off 16 October 2020); 14 patients have been dosed in the ongoing Study IMGN853-0417, Phase 3 safety and efficacy monotherapy study (data cut-off 22 October 2020); and 1 patient received MIRV in Study IMGN853-EAP-01, a single-agent, single-patient, expanded access study to

provide continued access to MIRV for a patient with advanced platinum-resistant primary peritoneal cancer.
TEAEs experienced by > 10% of patients in monotherapy Study IMGN853-0401 (expansion phase) or Study IMGN853-0403 (Phase 3 portion) clustered around the following SOCs: Gastrointestinal disorders, Eye disorders, General and administration site disorders, Nervous system disorders, Investigations, and Metabolism and nutrition disorders. The most common PTs within these SOCs were diarrhea, vision blurred, aspartate aminotransferase increased, nausea, vomiting, headache, asthenia, dry eye, keratopathy, decreased appetite, ALT increased, abdominal pain, fatigue, constipation, vomiting, visual acuity reduced, and peripheral neuropathy. The majority of TEAEs were ≤ Grade 2. The nature, frequency, and severity of TEAE were consistent with the safety profile of MIRV, and there were no clinically significant differences between the EOC and Endometrial cohorts.

The most common SAEs in Study IMGN853-0401 and Study IMGN853-0403 included abdominal pain, constipation, diarrhea, large intestinal obstruction, nausea, small intestinal obstruction, vomiting, metastatic neoplasm, gastroenteritis, pneumonia, urinary tract infection, decreased appetite, dehydration, organizing pneumonia, and pneumonitis. The nature and frequency of SAEs experienced were consistent with the drug's known safety profile and/or underlying disease.

Review of preliminary safety data from Study 0402 (combination therapy), did not indicate any trend with regard to the nature, severity, or frequency of treatment-emergent adverse events seen in the combination regimens as compared to the known safety profiles of either mirvetuximab soravtansine or the marketed drug.

TEAEs experienced by > 10% of patients treated with MIRV in combination with BEV or Pembro or BEV + Carbo in the expansion phase of Study IMGN853-0402 and ≥ 10% patients treated with MIRV in combination with Carbo or PLD in the escalation phase of Study IMGN853-0402 clustered around the following SOCs: Gastrointestinal disorders, Eye disorders, General disorders and administration site conditions, Nervous system disorders, Respiratory, thoracic, and mediastinal disorders, and Metabolism and nutritional disorders. The majority of TEAEs were ≤ Grade 2 and consistent with underlying disease and/or the known safety profiles of each drug.
SAEs occurring in 3 or more BEV or Pembro or BEV + Carbo patients included intestinal obstruction, nausea, vomiting, pneumonitis, general physical health deterioration, and small intestinal obstruction. SAEs across expansion cohorts were consistent with the known safety profiles of the drugs administered.

There were no clinically significant trends identified in hematology, chemistry, coagulation, or ECG data.

Please refer to the Investigator Brochure for detailed safety information.

Mirvetuximab soravtansine received accelerated approval for the treatment of adult patients with FRα-positive, platinum-resistant epithelial ovarian, fallopian tube, or primary peritoneal cancer,

from the FDA on November 14, 2022.

Clinical Efficacy Data Related to PK

Study 0401 is a first-in-human (FIH) Phase 1 trial designed to establish the MTD and determine the recommended Phase 2 dose (RP2D) of mirvetuximab soravtansine when administered intravenously as a single agent in adult patients with FRα positive solid tumors who have relapsed or are refractory to standard therapies. The study includes two stages: dose escalation and dose expansion and two dosing schedules, Schedule A (mirvetuximab soravtansine on Day 1, every 21 days; Q3W) and Schedule B (mirvetuximab soravtansine on Days 1, 8, and 15, every 28 days; modified weekly schedule). The primary aim of the dose-escalation phase was to evaluate the safety and tolerability of mirvetuximab soravtansine, to identify the MTD, and to characterize the pharmacokinetics (PK) profile of mirvetuximab soravtansine for each of the two schedules. The dose escalation stage is complete and the RP2D for Schedule A was determined to be 6.0 mg/kg adjusted ideal body weight (AIBW). Schedule A (Q3W) was evaluated in the dose expansion cohorts, which are defined as follows:

a. Dose Expansion Cohort 1: Patients with Platinum-Resistant Epithelial Ovarian Cancer (EOC) – 40 patients planned/*46 enrolled (enrollment complete)*
b. Dose Expansion Cohort 2: Advanced or recurrent uterine cancer – 20 patients/*24 enrolled (enrollment complete)*
c. Dose Expansion Cohort 3: Patients with relapsed EOC, which is amenable to biopsy – 20 patients planned/*27 enrolled (enrollment complete)*
d. Dose Expansion Cohort 4: Relapsed/refractory NSCLC adenocarcinoma or bronchoalveolar carcinoma (BAC) – *not yet opened*
e. Dose Expansion Cohort 5: Patients with EOC which has relapsed following platinum-based therapy: this cohort of patients will self-administer prophylactic corticosteroid eye drops – 40 patients planned/*40 enrolled (enrollment complete)*

At the time of data cut off (January 31, 2018), preliminary PK data were available for up to a total of 172 patients dosed with mirvetuximab soravtansine on Schedule A (Q3W; N=154). Following the administration of the first dose of mirvetuximab soravtansine, mean exposure (Cmax and AUC0-inf) increased with dose in a generally proportional manner from 1.0 to 7.0 mg/kg. For Cycle 1 cohorts ≥ 1.0 mg/kg, mean t1/2 of mirvetuximab soravtansine ranged from ~79 to 121 hours across doses with no meaningful dose-dependence noted in CL or Vss. In the two lowest dose cohorts (0.15 & 0.5 mg/kg), there appeared to be a trend towards higher CL, however, due to low patient numbers in these groups definitive conclusions are not possible. Exposure metrics (Cmax and AUC0-inf) at Cycle 3 indicated that there was no meaningful accumulation following multiple doses of mirvetuximab soravtansine.

Study 0402 (FORWARD-II) is an open-label, Phase 1b, non-randomized study of mirvetuximab soravtansine in combination with Bevacizumab (BEV), Carboplatin (Carbo), Pegylated Liposomal Doxorubicin (PLD) or Pembrolizumab in adult patients with FRα positive advanced EOC, primary peritoneal, fallopian tube, or endometrial cancer. There is no apparent effect of combination therapy in Study 0402 on preliminary mirvetuximab soravtansine PK parameters, where values are generally consistent among Regimens A, B, C and D, and with data reported for Study 0401.

Single agent mirvetuximab soravtansine has a 26% confirmed overall response rate (95% CI 14%-41%), including 1 complete and 11 partial responses in FRα positive platinum-resistant EOC in the patient expansion cohort in study 0401 [11]. Median PFS (mPFS) was 4.8 months (95% CI 3.9-5.7 months). The overall response rate was 39% (95% CI 20%-62%) and the median PFS (mPFS) is 6.7 months (95% CI 3.9-11 months) in the subset of 16 patients with one to three prior regimens [11]. Evaluation of the efficacy of mirvetuximab soravtansine in combination with pembrolizumab at the R2PD is ongoing in Study 0402 in ovarian cancer patients.

Based on these data, mirvetuximab soravtansine 6 mg/kg AIBW in combination with pembrolizumab 200 mg on day 1 of every 21-day cycle has been recommended as the phase 2 dose and appears to have a tolerable risk benefit profile and thus represents an appropriate dose for further evaluation in this patient population.

Nonclinical Pharmacology

The suitability of FRα as a target in the treatment of solid tumors was demonstrated by immunohistochemistry (IHC). FRα was shown to have limited normal tissue expression and marked expression in solid tumors, particularly cancers of the ovary, endometrium, lung, and kidney. In vitro studies demonstrated that mirvetuximab soravtansine binds cell surface FRα with high apparent affinity (≤ 0.1 nM) and shows potent (IC50 ≤ 1 nM) and selective cytotoxicity against cells expressing FRα.

Nonclinical Pharmacokinetics

Upon binding to the FRα receptor, mirvetuximab soravtansine undergoes receptor-mediated internalization and subsequent lysosomal degradation, resulting in intracellular release of DM4-containing cytotoxic catabolites. Binding of DM4 and a second metabolite, S-methyl-DM4, to tubulin inhibits tubulin polymerization and microtubule assembly, causing cell death. The lipophilic molecules S-methyl DM4 and DM4 can also diffuse to neighboring cells and induce bystander killing. Mirvetuximab soravtansine demonstrated significant activity against FRα-positive xenografts. Partial and/or complete regressions in xenograft models of epithelial ovarian cancer and NSCLC were seen at doses well below its maximum tolerated dose (MTD).

Toxicology

Nonclinical toxicology studies were performed to assess the potential clinical risks of mirvetuximab soravtansine, to define the maximum recommended starting dose (MRSD) and to support initiation of the pivotal Phase 3 study (Study 0403). These included the following:

1. IHC tissue cross reactivity and binding studies to define the most representative toxicology species.
2. A Ki-67 IHC study to define the mitotic activity of FRα-positive normal human tissues.
3. A single dose GLP toxicology study in cynomolgus monkeys to define the MRSD.

4. A repeat dose (Q3W for 4 doses) GLP 3-month chronic toxicology study in cynomolgus monkeys to support initiation of the pivotal Phase 3 study (Study 0403).
5. Single dose acute toxicity studies with DM4 in mice and cynomolgus monkeys to assess toxicity of the mirvetuximab soravtansine cytotoxic payload.
6. Single dose acute toxicity studies in mice to compare the tolerability and toxicity of mirvetuximab soravtansine (M9346A-sulfo-SPDB-DM4), which contains the novel sulfo-SPDB linker to an SPDB-DM4 conjugate (M9346A-SPDB-DM4), for which extensive preclinical toxicity data exists.

Toxicology Summary

Single dose (1, 3, 5, or 10 mg/kg) and 3-month repeat-dose (2, 4, or 8 mg/kg Q3 weekly) GLP toxicology studies were conducted in cynomolgus monkeys (a cross reactive species). The principal findings of the single-dose were changes in hematology parameters, depletion of bone marrow and lymphoid tissue, changes in serum chemistry, and injection site reactions. All mirvetuximab soravtansine-related events were considered non-adverse and had returned to normal or showed significant restoration towards normal by Day 29. The principal findings of the repeat dose study were similar and included hematologic abnormalities, electrolyte alterations, dermal reactions at the injection site and other skin areas and ophthalmic findings. The no-observed-adverse-effect level (NOAEL) established from repeat doses was 4 mg/kg.

Interactions

The risk of a clinically significant in vivo drug-drug interaction caused by DM4 inhibition of CYP3A is unknown; therefore, treatment of patients with concomitant medications that are sensitive substrates of CYP3A or are CYP3A substrates with a narrow therapeutic index should be carefully monitored (Section 5.4). Drinking greater than one serving (250 ml) of grapefruit juice per day should be avoided.

2.3 Rationale

Endometrial cancers have been historically classified as either type I or type II cancers. Type I cancers, which are typically of endometrioid histology, comprise approximately 85% of endometrial cancers and are linked to increased estrogens, obesity, hormone receptor positivity and generally favorable prognosis. Type II cancers include non-endometrioid cases, most commonly of papillary serous histology, and are more common in older, non-obese women and have a worse outcome. Recent identification of distinct genomic subtypes of endometrial cancer from The Cancer Genome Atlas (TCGA) has opened the opportunity to stratify response to therapy by subtype. This study examined 373 primary endometrioid and serous endometrial cancers and identified four major molecular subgroups associated with outcome: POLE-mutated, microsatellite unstable (MSI-H), copy number low, and copy number high groups [12]. POLE-mutated and MSI-H endometrial cancers are associated with high number of tumor-infiltrating lymphocytes and high neoantigen load [13], and are predicted to respond well to immunotherapy. Although outcomes are favorable in these two subgroups, the majority of endometrioid (72%) and serous (98%) of cases were classified in the copy number low or copy number high groups,

which lacked evidence of microsatellite instability and were associated with inferior outcome.

There are limited data on the use of immune checkpoint inhibitors in endometrial cancer in the clinic. Le *et al.* evaluated pembrolizumab in a phase II study of patients with or without mismatch repair deficiency. This study enrolled nine patients with non-colorectral mismatch repair-deficient tumors, and in this cohort, which included two endometrial cancer patients, there was an objective response rate of 71% (five out of seven) patients [14]. In the ongoing, multicenter phase 2 study of pembrolizumab in patients with non-colorectal MSI-H tumors (KEYNOTE-158), enrolled 4 endometrial cancer patients. The overall response rate at the time of reporting at ASCO 2016 was 42.9% with a disease control rate of 66.7% with 8 confirmed responses, and 1 complete response among the 21 MSI-H non-colorectal cancer cases. The responses appeared durable, and were continuing at the time of report [15]. The endometrial cancer cohort of the KEYNOTE-28 pembrolizumab study selected patients for PD-L1 expression (but did not evaluate mutation burden or MSI status) and enrolled 24 heavily pretreated patients. The objective response rate was 26% with 3 patients achieving partial response and three patients achieving stable disease, and all responses appeared durable, with two patients remaining on treatment at the time of reporting [16].

Pembrolizumab is now FDA approved in MSI tumors, including endometrial cancers, which have failed standard treatment options. Responses to checkpoint inhibitors alone in MSS patients are likely to be modest, though have not yet been reported. Strategies are needed to improve response rates to immunotherapy in MSS patients.

Folate receptor-alpha (FRα) is a receptor expressed on the surface of many solid tumors, including endometrial cancer. It is present in limited amounts in normal endometrium, however is highly expressed in endometrial hyperplasia and endometrial cancers [17]. Moderate to strong staining intensity is associated with increased grade, stage and poor prognosis in endometrial cancers [18]. Mirvetuximab soravtansine is an antibody drug conjugate (ADC) comprised of a FRα-binding antibody conjugated to the maytansinoid DM4 by a cleavable linker. DM4 is a potent tubulin-targeting agent. Early clinical evaluation of this drug has had good tolerability and encouraging single-agent activity in several cancer types, including endometrial cancer. In a Phase 1 expansion trial mirvetuximab soravtansine in previously treated patients with FRα positive advanced or recurrent endometrial cancer, there were 2 confirmed partial responses and 11 with stable disease out of 24 patients for an overall clinical benefit rate greater than 50% [19]. In the study, 16/24 (67%) tumors were of serous histology, and 5/24 (21%) were endometrioid histology. Antitumor activity was particularly pronounced in serous histology.

In addition to its direct toxicity on tumor cells bearing the folate receptor target, there are also emerging preclinical data that an additional mechanism of action of ADCs are on the local tumor immune microenvironment [20-22]. Most antibody drug conjugates in development use maytansinoid (DM1 or DM4, eg. T-DM1 Kadcycla), or auristatins (MMAE, eg. Brentuximab Vendotin) which are microtubule destabilizers. These agents have been identified as potent activators of dendritic cells. Mueller *et al.* showed that the MMAE and the MMAE analog, dolastatin were able to promote tumor directed immunity by inducing the maturation of dendritic cells, increased migration of dendritic cells to tumor-draining lymph nodes, and promoted proliferation and activation of CD8+ T-cells, essentially acting as an adjuvant in a vaccination

setting [21]. While the mechanism of how anti-tubulin agents activate dendritic cells in the tumor microenvironment is not yet understood, preclinical data suggest that tumor cytotoxicity is not sufficient, as other cytotoxic agents including cisplatin and etoposide were not sufficient to activate dendritic cells, and potent dendritic cell activation by dolastatin occurred in the absence of tumor cell death. The mirvetuximab soravtansine ADC also activates monocytes and promotes phagocytosis of mirvetuximab-treated FRα-positive tumor cells through a mechanism of Fc-FcγR interaction [22].

In addition to effects of ADCs in monotherapy, there is strong preclinical rationale for the combination of ADCs with immune checkpoint inhibitors. Treatment of mice bearing a Her2-expressing orthotopic tumor model with the anti-Her2 ADC T-DM1 promoted increased tumor-associated macrophage PD-L1 expression [20]. Combined treatment of orthotopic models with T-DM1 and anti CTLA-4/PD-1 caused complete tumor regression in a model with primary resistance to immunotherapy, and was accompanied by massive T-cell activation, proliferation, and increased tumor T-cell infiltration [20]. Similarly, in a preclinical study of the MMAE analog dolastain 10, treatment of immune-competent mice bearing MC38 mouse adenocarcinoma xenografts with systemic dolastatin 10 synergized with anti CTLA4/PD-1 to cause complete tumor regression in 7/12 animals [21].

In a phase 1 dose finding and dose expansion clinical trial, monotherapy with the ADC mirvetuximab soravtasine had a tolerable safety profile and demonstrated promising results in patients with recurrent platinum-resistant ovarian cancer and recurrent endometrial cancer. Based on these results, a multi-arm phase Ib/II clinical trial (FORWARD II, NCT02606305) of mirvetuximab soravtansine in combination with carboplatin, liposomal doxorubicin, bevacizumab, or pembrolizumab was developed and preliminary safety results demonstrate the combination is tolerable [23]. A dose expansion phase is ongoing. The response of microsatellite stable endometrial cancer to immune checkpoint inhibitor monotherapy is expected to be low, and combination therapy with immune-stimulatory drug-antibody conjugates is particularly relevant in this population. Thus, we propose in this study to measure the response of FRα positive, microsatellite stable advanced and recurrent endometrial cancers to the ADC mirvetuximab soravtansine in combination with the PD-1 checkpoint inhibitor, pembrolizumab.

2.4 Correlative Studies Background

Correlative studies may be performed in the Immune Oncology and DNA Repair Centers here at DFCI and Ventana Medical Systems. Depending on funding availability these may include: i) determination of CD3+, CD8+ TILs, CD8+/CD4+FOXP3+ TIL ratio, CD137+CD8+ TILs, CD137+CD8+/CD4+FOXP3+ TIL ratio, peritumoral lymphocytes and correlation with response; ii) expression of immune checkpoints including TIM-3, LAG-3, CTLA-4, PD-L2, PD-L1, PD-1, IDO and correlation with response; iii) targeted next generation sequencing (Oncopanel) to determine specific genetic alterations and correlate with response

The identification of predictive correlates to response to treatment with checkpoint blockade has proven a critical, but elusive goal, with numerous proposed approaches across different tumor types, but none that has thus far gained widespread clinical acceptance [24, 25]. Several efforts have focused on the identification of PD-L1 expression via immunohistochemistry (IHC) on the

surface of tumor, or infiltrating immune cells. Some studies have suggested a significant correlation between tumoral PD-L1 expression and response in nonsmall cell lung cancer [26], although others utilizing a similar approach have failed to identify a significant correlation [27, 28]. Notable challenges in this strategy include the lack of standardization among IHC assays, the use of differential thresholds for assay positivity and a lack of consensus regarding the importance of explicitly accounting for PD-L1 expression on infiltrating immune cells. Complicating the development of these approaches are concerns that inadequate negative predictive value and full understanding of the underlying immune dynamics between the host and tumor may result in the exclusion of patients who may benefit from treatment.

For this trial we may characterize PD-L1 and PD-L2 expression on tumor cells and infiltrating immune cells in baseline formalin fixed paraffin embedded (FFPE) blocks of cancer tissue (which are required for eligibility) in conjunction with the Dana Farber Cancer Institute Center for Immunooncology. We may also characterize CD3+ T-cells, CD8+ T-cells and CD4+ T-cells as well as other immune cell markers and checkpoints in baseline FFPE blocks of cancer tissue..

Although less advanced in clinical development and practice, characterization of ex vivo surface markers on tumor cells, T-cells and other immune cells via multiparametric analysis (e.g. via flow cytometry or mass cytometry) has significant potential to reveal predictive biomarkers given the ability to measure multifarious combinations and to identify combinatorial patterns of expression among diverse cell populations. Using multiparametric assays developed in conjunction with the Dana Farber Cancer Institute Center for Immunooncology, we may determine the relative frequencies of T-cell subsets and evaluate the expression of immune checkpoints PD-1, PD-L1, PD-L2 and other immunologically active markers including TIM-3, LAG-3, CTLA-4, and IDO.

Genomic predictive correlates for response to checkpoint blockade have taken several forms. First, and serving as a critical line of evidence supporting this trial, numerous studies have suggested a correlation between mutational and neoepitope burden and response to checkpoint blockade [14, 29, 30]. Furthermore, as noted above, identification of microsatellite instability resulting from defects in the mismatch repair pathway and specifically within MLH1, MSH2, MSH6 and PMS2 have been suggested to portend a significantly improved response to checkpoint blockade [14]. This trial is limited to MSS tumors, therefore additional genomic alterations that correlate with response to the combination may be sought by characterizing genomic changes in FFPE tissue specimen. These include specific DNA gene repair mutations, neoantigens, and single nucleotide polymorphisims (SNPs) in immunologically relevant genes.

PARTICIPANT SELECTION

Remote consenting procedures may be implemented in the FRα pre-screening process for this trial as allowed per institutional policy.

3.1 Eligibility Criteria

3.1.1 Participants must have advanced or recurrent **serous endometrial cancer**. Patients with mixed histologies/tumors are eligible if the serous component is the dominant histological subtype. In addition, the tumors must be:

1) microsatellite stable **(MSS)** as documented by either intact immunohistochemical (IHC) nuclear expression of the mismatch repair genes MSH2, MSH6, MLH1 and PMS2; or microsatelitte stable by polymerase chain reaction (PCR), next generation sequencing, or other CLIA-approved method;

AND

2) **FRα positive** by central immunohistochemistry (IHC, Section 9.1). If archival tissue does not meet FRα criteria, a fresh biopsy tumor sample may be submitted and used to meet this criterium. If a fresh tumor biopsy cannot be done safely the patient will not be allowed to enroll on this study.

3.1.2 Participants must have measurable disease as defined by RECIST 1.1. Lesions situated in a previously irradiated area are considered measurable if progression has been demonstrated in such lesions.

3.1.3 Prior therapy: Patients must have had one, but no more than four lines of chemotherapy for endometrial carcinoma.

- Prior hormonal therapy is allowed (no washout period is required after hormonal therapy) and does not count as a prior line of therapy. Hormonal therapy in combination with CDK4/6 inhibitors or mTOR or other PI3K-pathway inhibitors is allowed and does not count as a line of prior therapy.
- Prior IO therapy targeted to the PD-1/PD-L1 pathway is allowed in up to 19 patients of the total cohort.
- Patients must NOT have received prior therapy with any folate receptor ortholog agents.

3.1.4 Age 18 or greater years. Because insufficient dosing or adverse event data are currently available on the use of mirvetuximab soravtansine and pembrolizumab in participants <18 years of age, children are excluded. Endometrial cancer is rare in the pediatric population.

3.1.5 ECOG performance status 0 or 1

3.1.6 Availability of archival tissue for research purposes in the form of a formalin-fixed paraffin embedded (FFPE) block or 5 unstained slides (Section 9). Participants without available archival tissue may be eligible for enrollment after discussion with the Principal Investigator.

3.1.7 Participants must have normal organ and marrow function as defined below:

– leukocytes	≥3,000/mcL
– absolute neutrophil count	≥1,500/mcL

–	platelets	≥100,000/mcL
–	hemoglobin	≥ 9.0 g/dL
–	total bilirubin	≤ 1.5 x institutional upper limit of normal
–	AST(SGOT)/ALT(SGPT)	≤2.5 × institutional upper limit of normal
–	creatinine	≤ institutional upper limit of normal
		OR
–	creatinine clearance	≥40 mL/min/1.73 m^2 for participants with creatinine levels above institutional normal.

3.1.8 Time from prior therapy:
a. Systemic anti-neoplastic therapy: 5 half-lives or 4 weeks, whichever is shorter. Hormonal therapy is not considered anti-neoplastic therapy.
b. Radiotherapy: wide-field radiotherapy (e.g. > 30% of marrow-bearing bones) completed at least 4 weeks, or focal radiation completed at least 2 weeks, prior to starting study treatment

3.1.9 The effects of agents used in this study on the developing human fetus are unknown. For this reason, women of child-bearing potential must agree to use adequate contraception (hormonal or barrier method of birth control; abstinence) prior to study entry, during study treatment, and for at least twelve weeks after the last dose of study treatment. Should a woman become pregnant or suspect she is pregnant while she is participating in this study, she should inform her treating physician immediately.

3.1.10 Women of child-bearing potential must have a negative serum pregnancy test within 3 days prior to the first dose of study treatment.

3.1.11 Ability to understand and the willingness to sign a written informed consent document.

3.2 Exclusion Criteria

3.2.1 Participants who have had chemotherapy within 5 half-lives or 4 weeks (whichever is shorter) or radiotherapy within 2 weeks prior to entering the study. Patients completing wide-field radiotherapy (e.g. >30% of marrow-bearing bones) must not have had treatment within 4 weeks prior to entering study. Participants must have recovered from all AEs due to previous therapy to Grade 1 ≤ or baseline, except alopecia. Participants with endocrine-related AEs who are adequately treated with hormone replacement are eligible.

3.2.2 Participants who are receiving any other investigational agents.

3.2.3 Participants with prior exposure to IO agents targeting the PD-1/PD-L1 pathway who discontinued therapy due to treatment-related toxicity deemed to be specifically related to IO therapy.

3.2.4 Required use of folate-containing supplements (e.g. folate deficiency).

3.2.5 Known active CNS metastases and/or carcinomatous meningitis. Participants with previously treated brain metastases may participate provided they are radiologically stable, i.e. without evidence of progression for at least 4 weeks by repeat imaging (note that the repeat imaging should be performed during study screening), clinically stable and without requirement of steroid treatment for at least 14 days prior to first dose of study treatment.

3.2.6 History of allergic reactions attributed to compounds of similar chemical or biologic composition to monoclonal antibodies (including antibody drug-conjugates or checkpoint inhibitors).

3.2.7 Uncontrolled intercurrent illness including, but not limited to, any of the following within 6 months of first study treatment: symptomatic congestive heart failure, unstable angina pectoris, uncontrolled hypertension (≥ Grade 3), hypertensive crisis or hypertensive encephalopathy, uncontrolled cardiac arrhythmias, thrombotic or ischemic stroke, clinically-significant vascular disease (e.g. aortic aneurysm, or dissecting aneurysm), severe aortic stenosis, clinically significant peripheral vascular disease, or ≥ Grade 3 cardiac toxicity following prior chemotherapy, cardiac arrhythmia, or psychiatric illness/social situations that would limit compliance with study requirements.

3.2.8 Active or chronic corneal disorder, including but not limited to the following: Sjogren's syndrome, Fuchs corneal dystrophy (requiring treatment), history of corneal transplantation, active herpetic keratitis, and also active ocular conditions requiring on-going treatment/monitoring such as wet age-related macular degeneration requiring intravitreal injections, active diabetic retinopathy with macular edema, presence of papilledema, and acquired monocular vision.

3.2.9 Serious clinically-relevant active infection, including known HIV infection, varicella-zoster virus, cytomegalovirus infection, has a known history of Hepatitis B (defined as Hepatitis B surface antigen [HbsAg] reactive) or known active Hepatitis C virus (defined as HCV RNA is detected) or any other known concurrent infectious disease requiring IV antibiotics with within 2 weeks of study enrollment are ineligible because of the potential for immune side effects.

3.2.10 Has received a live vaccine or live-attenuated vaccine within 30 days prior to the first dose of study drug. Administration of killed vaccines is allowed.

3.2.11 Current or prior use of immunosuppressive medication within 7 days prior to enrollment with the following exceptions to this exclusion criterion:

a. Intranasal, inhaled, topical steroids, or local steroid injections (eg, intra-articular injection);
b. Systemic corticosteroids at physiologic doses not to exceed 10 mg/day of prednisone or equivalent;
c. Steroids as premedication for hypersensitivity reactions (eg, CT scan premedication).

3.2.12 Active autoimmune disease that might deteriorate when receiving an immunostimulatory agent. Patients with diabetes type I, vitiligo, psoriasis, hypo-or hyperthyroid disease not requiring immunosuppressive treatment are eligible.

3.2.13 Has a history of (non-infectious) pneumonitis/interstitial lung disease that required steroids or has current pneumonitis/interstitial lung disease.

3.2.14 Pregnant or nursing women are excluded from this study because effects of agents used in this study on infants or the developing human fetus are unknown

3.2.15 Presence of other malignancies unless they are considered cured by patient's oncologist.

3.3 Inclusion of Women and Minorities

Women of all races and ethnic groups are eligible for this trial. This disease does not affect men.

REGISTRATION PROCEDURES

4.1 General Guidelines for DF/HCC Institutions

Institutions will register eligible participants in the Clinical Trials Management System (CTMS) OnCore. Registrations must occur prior to the initiation of any protocol-specific therapy or intervention. Any participant not registered to the protocol before protocol-specific therapy or intervention begins will be considered ineligible and registration will be denied.

An investigator will confirm eligibility criteria and a member of the study team will complete the protocol-specific eligibility checklist.

Following registration, participants may begin protocol-specific therapy and/or intervention. Issues that would cause treatment delays should be discussed with the Overall Principal Investigator (PI). If the subject does not receive protocol therapy following registration, the subject must be taken off-study in the CTMS (OnCore) with an appropriate date and reason entered.

4.2 Registration Process for DF/HCC Institutions

Applicable DF/HCC policy (REGIST-101) must be followed.

4.3 General Guidelines for Other Investigative Sites

Eligible participants will be entered on study centrally at the Dana-Farber Gynecologic Oncology Coordinating Center by the Project Manager. All sites should contact the Project Manager (Martin Hayes; Martin_Hayes@dfci.harvard.edu) when a potential participant is identified to confirm enrollment availability.

Following registration, participants should begin protocol therapy within 5 days. Issues that would cause treatment delays should be discussed with the Overall PI. If the subject does not receive protocol therapy following registration, the subject must be taken off study in the CTMS (OnCore) with an appropriate date and reason entered.

4.4 Registration Process for Other Investigative Sites

To register a participant, the following documents should be completed by the participating non-DF/HCC site and e-mailed (Martin_Hayes@dfci.harvard.edu) to the Project Manager:

- Copy of required laboratory tests: complete blood count with differential and serum chemistries (including liver function tests)
- Screening clinic note (including documentation of oncologic history, past medical history, concomitant medications, performance status, and vitals)
- Pathology report (with documentation of MSS status)
- CT scan (with documentation of measurable disease per RECIST 1.1)
- Documentation of FRα positivity
- Clinic note from pulmonary function test
- Clinic note from eye exam
- Documentation of consent process
- Signed informed consent form
- HIPAA authorization form (if separate from the informed consent document)
- Eligibility Checklist

The participating site will then e-mail (Martin_Hayes@dfci.harvard.edu) the Project Manager to verify eligibility. The Project Manager will follow DF/HCC policy (REGIST-101) and register the participant on the protocol. The Project Manager will e-mail the participant study number, and if applicable the dose treatment level, to the participating site.

TREATMENT PLAN

5.1 Treatment Regimen

Treatment will be administered on an outpatient basis.

Following premedications, mirvetuximab soravtansine will be administered at 6 mg/kg by Adjusted Ideal Body Weight (AIBW) intravenously on Day 1 of a 21-day cycle. Then, pembrolizumab will be administered intravenously over 30 minutes at 200 mg flat dose on Day 1 of 21-day cycle (one cycle =21 days), after all procedures and assessments have been completed. Study medications may be administered up to 3 days before or after the scheduled day of administration of each cycle due to administrative reasons.

The duration of treatment with the combination will be until progression or adverse event up to 35 cycles. Beyond 35 cycles, mirvetuximab soravtansine will be continued until progression or adverse event.

Agent	Dose	Route	Schedule	Cycle Length
Mirvetuximab soravtansine (IMGN853)*	6 mg/kg (AIBW)	IV before Pembrolizumab +/- 10 min**	Day 1 each cycle	21 days (3 weeks)
Pembrolizumab	200 mg	IV over 30 minutes -5/+10 min	Day 1 each cycle	
*Note: Mirvetuximab soravtansine is incompatible with 0.9% sodium chloride (saline)<br>** Infusion rate will vary based on patient tolerability as described in Section 5.3.1				

Reported adverse events and potential risks are described in Section 7. Appropriate dose modifications are described in Section 6. No investigational or commercial agents or therapies other than those described below may be administered with the intent to treat the participant's malignancy.

5.2 Pre-Treatment Criteria

5.2.1 Cycle 1, Day 1 (or within 7 days prior to Cycle 1, Day 1)

- Absolute neutrophil count ≥1,500/mcL
- Platelets ≥100,000/mcL
- Hemoglobin ≥ 9g/dL
- Total bilirubin ≤ 1.5 x institutional upper limit of normal
- AST (SGOT) / ALT (SGPT) ≤2.5 x institutional upper limit of normal
- Creatinine ≤ institutional upper limit of normal or creatinine clearance ≥ 40 mL/min/1.73 m2 for subjects with creatinine levels above institutional normal
- All toxicities of previous therapy (aside from alopecia) must have resolved to ≤ grade 1
- ECOG performance of 0 or 1
- No evidence of life-threatening medical problems

5.2.2 Subsequent Cycles

- Absolute neutrophil count ≥1,000/mcL
- Platelets ≥75,000/mcL
- AST (SGOT) / ALT (SGPT) ≤ 2.5 x institutional upper limit of normal
- Creatinine ≤ institutional upper limit of normal or creatinine clearance >= 40 mL/min/1.73 m2 for subjects with creatinine levels above institutional normal
- All toxicities pf previous cycles must have resolved to ≤ grade 2
- ECOG performance of 0 to 2
- No evidence of life-threatening medical problems

5.3 Agent Administration

5.3.1 Mirvetuximab Soravtansine (IMGN853)

<u>Dosing</u>

Mirvetuximab soravtansine 6 mg/kg by Adjusted Ideal Body Weight (AIBW) IV on Day 1 of each 21-day cycle. Dosing by AIBW as follows:

Adjusted Ideal Body Weight (AIBW)
IBW[1] + 0.4 (Actual weight – IBW[1])

Where:

Ideal Body Weight (IBW)
IBW[1] (male) = 0.9H[1]-88
IBW[1] (female) = 0.9H[1]-92

([1]H=height in cm; W=weight in kg)

The weight used for calculation should be obtained prior to study drug administration on Cycle 1 Day 1 (+/- 14 days) and thereafter should only be modified for significant (≥ 10%) changes in body weight (not influenced by weight gain or loss attributed to fluid retention).

<u>Preparation and Administration</u>

Only qualified personnel who are familiar with procedures that minimize undue exposure to them and to the environment should undertake the preparation, handling, and safe disposal of chemotherapeutic and investigational agents. Mirvetuximab soravtansine will be administered at the investigational site. Investigational product should be prepared and dispensed by an appropriately qualified and experienced member of the study staff (eg, physician, nurse, physician's assistant, practitioner, or pharmacist) as allowed by local, state, and institutional guidance

The desired amount should be withdrawn from the vial(s) and diluted using 5% dextrose or 5% D-glucose to a final concentration as outlined in Section 8.1.7. **Note: Mirvetuximab soravtansine is incompatible with saline (0.9% sodium chloride).** Therefore, dilutions must be made using 5% dextrose or 5% D-glucose. Infusion bags must be labeled with the protocol number, patient number, storage temperature, dose, and volume of mirvetuximab soravtansine filtered into the bag, or according to institutional protocol. Light protection is not required for prepared IMGN853. The infusion must be completed within 8 hours of preparation.

If necessary, study drug from different drug lots may be mixed in a single-dose administration.

Mirvetuximab soravtansine should be administered at a rate of 1 mg/min; after 30 minutes, the rate can be increased to 3 mg/min if well tolerated. If well tolerated after 30 minutes at 3 mg/min, the mirvetuximab soravtansine infusion rate may be increased to 5mg/min. Subsequent infusions may be delivered at the tolerated rate. For patients who have been administered mirvetuximab soravtansine as a prior anti-cancer therapy, mirvetuximab soravtansine should be administered at the tolerated rate of prior mirvetuximab soravtansine infusions. Therefore, the overall length of infusion will vary depending on dose and patient tolerability. Following

infusion, the IV line should be flushed with 5% dextrose or 5% D-glucose as needed to ensure delivery of the full dose.

In combination, mirvetuximab soravtansine should be administered first, followed by pembrolizumab. Mirvetuximab soravtansine will be administered as an IV infusion following preparation as outlined in Section 8.1.7.

Drug, Tubing and Filtration

An IV tubing administration set with a 0.2 micron or 0.22 micron in-line filter must be used for infusion. Refer to section 8.1.4 for more information.

Observation Period

Patients will be carefully observed during each infusion and vital signs taken as outlined in the Study Calendar. They will remain in the clinic under observation for 2 hours after the completion of the first infusion of mirvetuximab soravtansine, and for at least 1 hour after each subsequent infusion, during which pembrolizumab infusion can be administered. The observation period will start after completion of the mirvetuximab soravtansine infusion for all infusions.

Infusion Reactions

Some patients treated with IV infusions of therapeutic drugs have experienced concurrent infusion-related reactions (see CTCAE Version 5.0). The signs and symptoms may vary and include for example, headache, fever, facial flushing, pruritus, myalgia, nausea, chest tightness, dyspnea, vomiting, erythema, abdominal discomfort, diaphoresis, shivers, lightheadedness, hypotension, palpitations, and somnolence. Anaphylaxis might occur at any time during an infusion. Before any infusion is started, appropriate medical personnel, medication (e.g. epinephrine, inhaled beta agonists, antihistamines, and corticosteroids), and other required resources to treat anaphylaxis must be readily available. In general, Investigators should manage acute allergic or hypersensitivity reactions according to institutional practices. General guidelines for the management of acute infusion-related reactions and for subsequent retreatment are provided in Table 1. Delayed infusion-related reactions may occur; therefore, patients should be advised to seek immediate medical treatment if symptoms newly develop and/or recur after discharge from clinic.

Table 1: Management Guidelines for Potential Mirvetuximab Soravtansine Infusion-related Reactions

CTCAE v5.0 Severity Grade	Management
**Grade 1**<br>Mild reaction; infusion interruption not indicated; intervention not indicated	• Maintain infusion rate unless progression of symptoms to ≥ Grade 2; if symptoms worsen, refer to guidelines below<br>• Promethazine (or equivalent) 150 mg PO per day for nausea (or equivalent)<br>• Diphenhydramine (or equivalent) 25-50 mg PO or IV prn<br>• Methylprednisolone 125 mg (or equivalent) IV prn
**Grade 2**<br>Therapy or infusion interruption indicated but responds promptly to symptomatic treatment (e.g., antihistamines, NSAIDs, narcotics, IV fluids); prophylactic medications indicated for ≤24 hrs	• Interrupt infusion and disconnect infusion tubing from patient<br>• Promethazine (or equivalent) 150 mg PO per day for nausea<br>• Diphenhydramine (or equivalent) 25-50 mg PO or IV prn<br>• Acetaminophen (or equivalent) 650 mg PO prn<br>• Methylprednisolone 125 mg (or equivalent) IV prn<br>• After recovery from symptoms, resume the infusion at 50% of the previous rate and if no further symptoms appear, gradually increase rate until infusion is completed.<br>• For subsequent dosing in future cycles, patients should be pre-medicated with dexamethasone (or equivalent) 8 mg PO BID the day prior to drug administration and acetaminophen (or equivalent) 650 mg PO and diphenhydramine (or equivalent) 25-50 mg PO 30-60 minutes prior to dosing.
**Grades 3 or 4**<br>Grade 3: Prolonged (i.e. not rapidly responsive to symptomatic medication and/or brief interruption of infusion); recurrence of symptoms following initial improvement; hospitalization indicated for clinical sequelae<br>OR<br>Grade 4: Life-threatening consequences, urgent intervention indicated	• Immediately stop infusion and disconnect infusion tubing from subject<br>• Administer diphenhydramine (25-50 mg) IV (or equivalent)<br>• Administer IV steroids (methylprednisolone (or equivalent) up to 0.5mg/kg Q 6h) to treat ongoing reaction and prevent recurrence<br>• Administer bronchodilators (nebulized albuterol/salbutamol, 2.5-5 mg in 3 mL of saline or equivalent) as medically indicated<br>• Administer normal saline as medically indicated<br>• Administer epinephrine (0.2-0.5 mL of a 1:1000 dilution (0.2-0.5 mg) SQ or IM) as medically indicated. Epinephrine should only be used if all other treatment methods fail to manage the infusion-related reaction.<br>• Advise patient to seek emergency treatment and notify investigator/clinic if the infusion-related symptoms recur after discharge from clinic.<br>• Report as a serious adverse event (see Section 7)<br>• Permanently discontinue study medication treatment

<u>Order of Administration</u>

Mirvetuximab soravtansine should be administered first, followed by pembrolizumab.

<u>Caregiver Precautious</u>

Mirvetuximab soravtansine is an experimental anticancer drug, and, as with other potentially toxic compounds, caution should be exercised when handling this compound. It is recommended that gloves and protective garments be worn during preparation and clean-up of any spills or bodily fluids from a patient treated with mirvetuximab soravtansine. Refer to Section 8.1 and package inserts for more information.

5.3.2 Pembrolizumab (MK-3475)

<u>Dosing</u>

Pembrolizumab 200 mg as a 30 minute infusion on Day 1 of each 21-day cycles

Sites should make every effort to target infusion timing to be as close to 30 minutes as possible. However, given the variability of infusion pumps from site to site, a window of -5 minutes and +10 minutes is permitted (i.e., infusion time is 30 minutes: -5 min/+10 min).

<u>Drug, Tubing and Filtration</u>

The liquid drug product (DP) can be diluted with normal saline or 5% dextrose in the concentration range of 1 to 10 mg/mL in IV containers made of material listed in Section 8.2.7. Section 8.2.7 contains specific instructions for the preparation of the pembrolizumab infusion fluid and administration of infusion solution.

<u>Infusion Reactions</u>

Infusion-related reactions have been reported with pembrolizumab, and are non-immune-mediated events.

Pembrolizumab may cause severe or life threatening infusion-reactions including severe hypersensitivity or anaphylaxis. Signs and symptoms usually develop during or shortly after drug infusion and generally resolve completely within 24 hours of completion of infusion. Dose modification and toxicity management guidelines on pembrolizumab associated infusion reaction are provided in Table 2.

Table 2: Management Guidelines for Potential Pembrolizumab Infusion-related Reactions

CTCAE v5.0 Severity Grade	Treatment	Premedication at Subsequent Dosing
**Grade 1**<br>Mild reaction; infusion interruption not indicated; intervention not indicated	Increase monitoring of vital signs as medically indicated until the participant is deemed medically stable in the opinion of the investigator.	None
**Grade 2**<br>Therapy or infusion interruption indicated but responds promptly to symptomatic treatment (e.g., antihistamines, NSAIDs, narcotics, IV fluids); prophylactic medications indicated for ≤24 hrs	**Stop Infusion.**<br>Additional appropriate medical therapy may include but is not limited to:<br>IV fluids<br>Antihistamines<br>NSAIDs<br>Acetaminophen<br>Narcotics<br>Increase monitoring of vital signs as medically indicated until the participant is deemed medically stable in the opinion of the investigator.<br>If symptoms resolve within 1 hour	Participant may be premedicated 1.5h (± 30 minutes) prior to infusion of pembrolizumab with:<br>Diphenhydramine 50 mg po (or equivalent dose of antihistamine).<br>Acetaminophen 500-1000 mg po (or equivalent dose of analgesic).

	of stopping drug infusion, the infusion may be restarted at 50% of the original infusion rate (e.g. from 100 mL/hr to 50 mL/hr). Otherwise dosing will be held until symptoms resolve and the participant should be premedicated for the next scheduled dose.<br>**Participants who develop Grade 2 toxicity despite adequate premedication should be permanently discontinued from further study drug treatment**	
**Grades 3 or 4**<br>Grade 3:<br>Prolonged (i.e., not rapidly responsive to symptomatic medication and/or brief interruption of infusion); recurrence of symptoms following initial improvement; hospitalization indicated for other clinical sequelae<br>Grade 4:<br>Life-threatening consequences, urgent intervention indicated	**Stop Infusion.**<br>Additional appropriate medical therapy may include but is not limited to:<br>Epinephrine**<br>IV fluids<br>Antihistamines<br>NSAIDs<br>Acetaminophen<br>Narcotics<br>Oxygen<br>Pressors<br>Corticosteroids<br>Increase monitoring of vital signs as medically indicated until the participant is deemed medically stable in the opinion of the investigator.<br>Hospitalization may be indicated.<br>**In cases of anaphylaxis, epinephrine should be used immediately.<br>**Participant is permanently discontinued from further study drug treatment.**	No subsequent dosing
Appropriate resuscitation equipment should be available at the bedside and a physician readily available during the period of drug administration.<br>For further information, please refer to the Common Terminology Criteria for Adverse Events v5.0 (CTCAE) at http://ctep.cancer.gov/protocolDevelopment/electronic_applications/ctc.htm.		

Order of Administration

Mirvetuximab soravtansine should be administered first, followed by pembrolizumab.

5.4 General Concomitant Medication and Supportive Care Guidelines

All concomitant medications, over-the-counter, herbal supplements and supportive therapy taken within 28 days of Cycle 1, day 1 and through 30 days after last study treatment must be recorded on the appropriate eCRF. The identity of all medications, dosage, and route of administration,

frequency, duration of administration, and indication for use will be recorded in the appropriate sections of the eCRF.

5.4.1 Required Supportive Care Medications

5.4.1.1 Premedications

All patients must receive 325-650 mg of acetaminophen/paracetamol (PO or IV), 10 mg IV dexamethasone, and 25-50 mg diphenhydramine (PO or IV)(equivalent drugs of similar drug classes is also acceptable) approximately 30-90 minutes prior to each infusion of mirvetuximab soravtansine. If individual patients require more intensive treatment to prevent infusion-related reactions, investigators may modify the regimen accordingly.

5.4.1.2 Eye drops

Lubricating artificial tears and corticosteroid eye drops are mandatory for all patients receiving mirvetuximab soravtansine as follows (Appendix D):

- Patients are required to use corticosteroid eye drops as prescribed by the treating physician unless the risk outweighs the benefit as per the ophthalmologist/physician. All patients enrolled will be instructed to self-administer 1% prednisolone (Pred Forte® or generic equivalent) six times daily on Days 1-4 and four times daily on Days 5-8 of each cycle during the study. For individual patients who cannot tolerate the preservative contained in 1% prednisolone, other corticosteroid eye drops may be substituted (e.g. difluprednate 0.05%; Durezol®) and administered on Days 1-8 of each cycle at a frequency prescribed by the ophthalmologist.

- Patients will be required to use preservative-free, lubricating artificial tears on a daily basis (as directed by the product label or the treating physician). Patients should be advised to wait at least 15 minutes following steroid administration before instilling lubricating eye drops.

- The patient should continue use of the corticosteroid eye drops per the ophthalmologist recommendation after discontinuation of mirvetuximab soravtansine. The patient should continue the lubricating eye drops for 4 weeks following discontinuation of mirvetuximab soravtansine.

5.4.2 Other supportive care

- Antiemetic (e.g. 5-HT_3 serotonin receptor antagonists such as palonosetron, granisetron or ondansetron), antidiarrheal (e.g. loperamide) medications, and other supportive care may be used at the discretion of the treating physician, unless prohibited below in Section 5.4.3.

- Patients are strongly advised to avoid using contact lenses while on mirvetuximab soravtansine.

- Baby shampoo and a soft cloth should be used to clean the eyes, and a warm compress at bedtime may be used to decrease any possible inflammation on the eyelid's surface.

- The use of UVA/UVB sunglasses is recommended in full daylight during the course of the study.

- The use of temporary lower punctal plugs to increase lubrication of the eyes is optional if lubricating artificial tears and corticosteroid eye drops are not sufficient.

The management and dose modification for ocular symptoms is outlined in Section 6.

5.4.3 Prohibited Medications

Potent CYP3A4 inhibitors such as ketoconazole and erythromycin should be avoided during study treatment. *In vitro* metabolism studies demonstrated that DM4 is predominantly metabolized by thiol s-methyltransferase (TMT) to form Me-DM4, which is further metabolized into sulfoxide-methyl-DM4. As Me-DM4 has been shown to be primarily metabolized by CYP3A, its exposure could potentially be increased in the presence of strong CYP3A inhibitors. Drinking greater than 1 serving (250 mL) of grapefruit juice per day should be avoided.

In addition, participants are prohibited from receiving the following therapies during Screening and Treatment phase of the trial

- Antineoplastic systemic chemotherapy or biological therapy

- Immunotherapy not specified in this protocol

- Investigational agents other than the study drugs

- Folate-containing vitamins

- Growth factors (G-CSF, GM-CSF) cannot be used during cycle 1, but are allowed on subsequent cycles of treatment as per published ASCO or institutional guidelines.

- Available in vitro metabolism data suggest DM4 has a potential to inhibit CYP3A activity *in vivo*. The risk of a significant in vivo drug-drug interaction caused by DM4 inhibition of CYP3A is not currently known, therefore, treatment of patients with concomitant medications that are sensitive substrates of CYP3A or are CYP3A substrates with a narrow therapeutic index should be carefully monitored.

- Grapefruit juice and or the oral consumption of grapefruit should be avoided.

- Live vaccines within 30 days prior to the first dose of study treatment and while participating in the study. Examples of live vaccines include, but are not limited to, the following: measles, mumps, rubella, varicella/zoster, yellow fever, rabies, BCG, and typhoid vaccine. Seasonal influenza vaccines for injection are generally killed virus

vaccines and are allowed; however, intranasal influenza vaccines (eg, FluMist®) are live attenuated vaccines and are not allowed.

- Systemic glucocorticoids for any purpose other than to modulate symptoms from an event of clinical interest of suspected immunologic etiology. The use of physiologic doses of corticosteroids may be approved after consultation with the Overall PI.
- Contact lens use should be avoided during study treatment period

5.5 Criteria for Taking a Participant Off Protocol Therapy

Duration of therapy will depend on individual response, evidence of disease progression and tolerance. In the absence of treatment delays due to adverse event(s), treatment with both agents may continue for up to 35 doses (approximately 2 years), or with mirvetuximab soravtansine alone after 35 cycles, or until one of the following criteria applies:

- Disease progression (as defined in sections 5.5.1 and 5.5.2)
- Intercurrent illness that prevents further administration of treatment
- Unacceptable adverse event(s)
- Participant demonstrates an inability or unwillingness to comply with the oral medication regimen and/or documentation requirements
- Participant decides to withdraw from the protocol therapy
- General or specific changes in the participant's condition render the participant unacceptable for further treatment in the judgment of the treating investigator

Participants will be removed from the protocol therapy when any of these criteria apply. The reason for removal from protocol therapy, and the date the participant was removed, must be documented in the case report form (CRF). Alternative care options will be discussed with the participant.

When a participant is removed from protocol therapy and/or is off of the study, the relevant Off-Treatment/Off-Study information will be updated in OnCore.

5.5.1 Tumor Response Assessments

Antitumor activity will be assessed through radiological tumor assessments conducted at baseline (screening) of starting therapy and every 2 cycles (6 weeks) thereafter. In addition, radiological tumor assessments will also be conducted whenever disease progression is suspected (eg, symptomatic deterioration) and at the time of withdrawal from the treatment (if not done in the previous 4 weeks). Complete, partial responses and progressive disease must be confirmed on repeated imaging ≥ 4 weeks after initial documentation, but not more than 6 weeks after initial documentation.

Assessment of response will be made using RECIST version 1.1 and as per immune-related RECIST criteria (irRECIST).

5.5.2 Treatment after Initial Evidence of Radiologic Disease Progression

Immunotherapeutic agents such as pembrolizumab may produce antitumor effects by potentiating endogenous cancer-specific immune responses. The response patterns seen with such an approach may extend beyond the typical time course of responses seen with chemotherapeutic agents, and may manifest as a clinical response after an initial increase in tumor burden or even the appearance of new lesions.

If radiologic imaging shows progression, tumor assessment should be repeated > 4 weeks later in order to confirm progression, and study treatment may be continued at the Investigator's discretion while awaiting radiologic confirmation of disease progression. For the subjects who continue on study therapy beyond progression, further progression is defined as an additional 10% increase in tumor burden from time of initial PD. This includes an increase in the sum of diameters of all target lesions and/ or the diameters of new measurable lesions compared to the time of initial PD. Study treatment should be discontinued permanently upon documentation of further progression. If repeat imaging shows a reduction in the tumor burden demonstrating CR, PR or SD compared to the initial scan, treatment with study treatment may be continued or resumed.

5.6 Duration of Follow Up

Participants will be followed for 3 years by phone after removal from study or until death, whichever occurs first. Survival status should be checked every 6 months during that time. Participants removed from study for unacceptable adverse events will be followed until resolution or stabilization of the adverse event. Date and cause of death should be provided for participants who become deceased within the 3-year interval following removal from the study. Should a participant become pregnant while on trial, the patient will be withdrawn from the study. However, the outcome of the pregnancy and the newborn's health, if the pregnancy is carried to term, will be monitored.

5.7 Criteria for Taking a Participant Off Study

Patients may voluntarily withdraw from the study treatment at any time for any reason, and without prejudice to further treatment. In addition, patients may be withdrawn by the Investigator if they do not feel the patient is deriving clinical benefit or because the patient is experiencing unacceptable toxicity, patient is lost to follow up, or patient is not compliant with study procedures.

Patients who withdraw or are removed from the study treatment will have an off-treatment visit within 7 days of the decision to discontinue study treatment. The final visit will occur at the time the participant discontinues study medication. Following the Final Visit, participants will be followed for disease progression (if reason for treatment discontinuation is other than

progression) and survival, and reassessment for toxicity will be performed up to 30 days after the last treatment dose. Participants will be followed for up to 3 years by phone after removal from study or until death, whichever occurs first. Survival status should be checked every 6 months during that time

The reason for taking a participant off study, and the date the participant was removed, must be documented in the case report form (CRF). In addition, the study team will ensure Off Treatment/Off Study information is updated in OnCore in accordance with DF/HCC policy REGIST-101.

DOSING DELAYS/DOSE MODIFICATIONS

Every effort should be made to administer study treatment on the planned dose and schedule.

Dose delays and modifications will be made as indicated in the following table(s). The descriptions and grading scales found in the revised NCI Common Terminology Criteria for Adverse Events (CTCAE) version 5.0 will be utilized for dose delays and dose modifications. A copy of the CTCAE version 5.0 can be downloaded from the CTEP website http://ctep.cancer.gov/protocolDevelopment/electronic_applications/ctc.htm.

Patients experiencing AEs that can be clearly attributed to mirvetuximab soravtansine or pembrolizumab will have the corresponding agent modified or withheld. In case of an AE recurrence, the other drug in the combination regimen will then be modified or withheld. If a relationship of an AE to a particular drug cannot be clearly determined, the order in which the drugs are to be reduced will require approval from the Overall PI.

6.1 Mirvetuximab Soravtansine (IMGN853)

6.1.1 Monitoring and Management of Potential Ocular Disorders

In early dose escalation, there was a relationship between mirvetuximab soravtansine plasma exposure with increased likelihood of an ocular event as well as with response. Exposure-response modeling suggested that a dose of 6.0 mg/kg AIBW provided adequate exposure for response while also maintaining overall exposure within a range that decreased the potential for ocular adverse events. Due to the observation of ocular disorders consistent with reversible keratopathy/corneal epitheliopathy in patients treated with mirvetuximab soravtansine, ocular function will be carefully monitored. Ocular symptom assessments will be performed at baseline and at Day 1 of every cycle thereafter (Study Calendar and Appendix C). Complete ophthalmologic exams will be performed in all patients at baseline and every other cycle thereafter if there is a TEAE reported (Study Calendar and Appendix C).

Patients are strongly advised to avoid using contact lenses while on mirvetuximab soravtansine. Baby shampoo and a soft cloth should be used to clean the eyes, and a warm compress at bedtime may be used to decrease any possible inflammation on the eyelid's surface. Please refer

to Section 5.4 for details on the prophylactic use of steroid eye drops and lubricating artificial tears. The use of UVA/UVB sunglasses is recommended in full daylight during the course of the study. The use of temporary lower punctal plugs to increase lubrication of the eyes is optional if lubricating artificial tears and corticosteroid eye drops are not sufficient. If patients report signs or symptoms of ocular disorders, including, but not limited to, blurred vision or eye irritation, the management and dose modification is outlined below.

If a patient develops ocular symptoms of any grade, the patient is required to have a complete examination by an ophthalmologist. If a patient develops ≥ CTCAE Grade 2 ocular symptoms, treatment with mirvetuximab soravtansine will be interrupted. Therapy may resume if ocular symptoms improve to Grade 1 or baseline within 28 days of the next scheduled mirvetuximab soravtansine dose (refer to Table 3 for details). Subsequent eye examinations will be scheduled to occur in every other cycle going forward, from the time that the AE was initially reported, and at either the End of Treatment visit or 30-day follow-up visit following treatment discontinuation, even if the results of the patient's ocular exam shows no obvious clinical findings. Management of treatment-emergent ocular AEs with inflammatory characteristics should include corticosteroid eye drops and/or other measures as indicated by an ophthalmologist.

Table 3: Management of Ocular Disorders

CTCAE v5.0 Severity Grade*	Management	Guidelines for Mirvetuximab Soravtansine Dose Modifications
Grade 1	• Complete eye exam as outlined in Study Calendar and Appendix C.<br>• Monitor for worsening symptoms<br>• Repeat complete exam as clinically indicated, at least every other cycle (from point at which toxicity first reported) at a minimum	• Continue mirvetuximab soravtansine dosing
Grade 2	• Complete eye exam as outlined in schedule of clinical assessments (Study Calendar and Appendix C).<br>• Repeat complete exam as clinically indicated<br>• Patients should have weekly symptomatic ocular assessments until the symptoms resolve to Grade 1 or baseline or are deemed to irreversible by investigator<br>• Repeat complete exam as clinically indicated, at least every other cycle (from point at which toxicity first reported) at a minimum	• Hold mirvetuximab soravtansine dosing until AE has resolved to Grade 1 or better.<br>• Patients with ocular symptoms lasting < 14 days may be allowed to resume mirvetuximab soravtansine at the same dose level<br>• Patients with ocular symptoms lasting ≥ 14 days but no more than 28 days may resume mirvetuximab soravtansine reduced by one dose level

Grade 3	• Complete eye exam as outlined in schedule of assessments (Study Calendar and Appendix C). Repeat complete exam as clinically indicated.<br>• Patients should have weekly symptomatic ocular assessments until the symptoms resolve to Grade 1 or baseline or are deemed to be irreversible by the investigator.<br>• Repeat complete exam as clinically indicated, at least every other cycle (from point at which toxicity first reported) at a minimum	• Hold mirvetuximab soravtansine dosing.<br>• Patients may be allowed to resume mirvetuximab soravtansine reduced by one dose level after AE has resolved to Grade 1 or better within 28 days.
Grade 4	• Complete eye exam as outlined in Study Calendar and Appendix C Repeat complete exam as clinically indicated.<br>• Patients should have weekly symptomatic ocular assessments until the symptoms resolve to Grade 1 or baseline (Study Calendar and Appendix C) or are deemed to be irreversible by the investigator.<br>• Repeat complete exam as clinically indicated, at least every other cycle (from point at which toxicity first reported) at a minimum	• Permanently discontinue mirvetuximab soravtansine dosing.
* Appendix C includes CTCAE version 5.0 Grade definitions for select ocular disorders		

6.1.2 Monitoring of Non-Infectious Pneumonitis Following the Administration of Study Treatment

Non-infectious pneumonitis has been observed following the administration of mirvetuximab soravtansine and pembrolizumab and may result in fatigue, shortness of breath, cough or respiratory distress. Drug-induced pneumonitis may be immediately life-threatening. If a patient presents with signs or symptoms consistent with pneumonitis and/or a clinically meaningful change in pulse oximetry value, the patient should be immediately evaluated. Patients are advised to notify their treating physician immediately if they experience new or worsening shortness of breath, cough or respiratory distress.

For patients diagnosed with pneumonitis without an infectious etiology, the management and treatment guidelines outlined in Table 4 should be followed.

Table 4: Management of Pneumonitis

CTCAE v5.0 Severity	Dose Modifications	irAE management	Monitor and follow-

**Grade**		**with corticosteroid and/or other therapies**	**up**
Grade 1	Continue dosing after discussion with the Overall PI		
Grade 2	Withhold mirvetuximab soravtansine and pembrolizumab (both agents)<br><br>Mirvetuximab soravtansine can be resumed at a dose reduced by one dosing level (see Table 6), and pembrolizumab can be resumed at same dosing level after discussion with the Overall PI when symptoms resolve to ≤ Grade 1	Administer corticosteroids (initial dose of 1-2 mg/kg prednisone or equivalent) followed by taper*	• Monitor participants for signs and symptoms of pneumonitis<br>• Evaluate participants with suspected pneumonitis with radiographic imaging and initiate corticosteroid treatment<br>• Add prophylactic antibiotics for opportunistic infections
Grade 3 or 4, or recurrent Grade 2	Permanently discontinue mirvetuximab soravtansine and pembrolizumab (both agents)	Administer corticosteroids (initial dose of 1-2 mg/kg prednisone or equivalent) followed by taper*	• Monitor participants for signs and symptoms of pneumonitis<br>• Evaluate participants with suspected pneumonitis with radiographic imaging and initiate corticosteroid treatment<br>• Add prophylactic antibiotics for opportunistic infections

* Notes on steroids for irAE:

1. Corticosteroid taper should be initiated upon AE improving to Grade 1 or less and continue to taper over at least 4 weeks.
2. Pembrolizumab and mirvetuximab soravtansine can be resumed after AE has been reduced to Grade 1 or 0 and corticosteroid has been tapered. Both agents should be permanently discontinued if AE does not resolve within 12 weeks of last dose or corticosteroids cannot be reduced to ≤10 mg prednisone or equivalent per day within 12 weeks.
3. For severe and life-threatening irAEs, IV corticosteroid should be initiated first followed by oral steroid. Other immunosuppressive treatment should be initiated if irAEs cannot be controlled by corticosteroids.

6.1.3 Other symptoms

Mirvetuximab soravtansine dose may be reduced at the discretion of the Investigator after discussion with the Overall PI. Patients who develop adverse events requiring an interruption of mirvetuximab soravtansine may resume treatment at a reduced dose level as shown in Tables 5 and 6 provided the criteria outlined in Section 5.2 are met.

Table 5: Mirvetuximab Soravtansine Dose Modification Guidelines

Adverse Event	Management/Next Dose for Mirvetuximab Soravtansine
≤ Grade 1	No change in dose
Grade 2	Hold until ≤ Grade 1. Resume at same dose level.
Grade 3	Holduntil < Grade 2. Resume at one dose level lower..
Grade 4	Off protocol therapy

Table 6: Mirvetuximab Soravtansine Dose Levels

Dose Level	Mirvetuximab Soravtansine Dose
-2	4 mg/kg AIBW, Q21 day
-1	5 mg/kg AIBW, Q21 day
0	6 mg/kg AIBW, Q21 day

A second dose reduction will be permitted for second event of Grade 3 or higher. Dose reductions below 4 mg/kg (Dose Level -2) will not be permitted.

Mirvetuximab soravtansine may be interrupted for situations other than treatment-related Aes such as medical / surgical events or logistical reasons not related to study therapy. Participants should be placed back on study therapy within 3 weeks of the scheduled interruption, unless otherwise discussed with the Overall PI. The reason for interruption should be documented in the patient's study record.

6.2 Pembrolizumab

AEs associated with pembrolizumab exposure, including coadministration with additional compounds, may represent an immunologic etiology. These immune-related AEs (irAEs) may occur shortly after the first dose or several months after the last dose of pembrolizumab/combination treatment and may affect more than one body system simultaneously. Therefore, early recognition and initiation of treatment is critical to reduce complications. Based on existing clinical study data, most irAEs were reversible and could be managed with interruptions of pembrolizumab/combination treatment, administration of corticosteroids and/or other supportive care. For suspected irAEs, ensure adequate evaluation to confirm etiology or exclude other causes. Additional procedures or tests such as bronchoscopy, endoscopy, skin biopsy may be included as part of the evaluation. Based on the severity of

irAEs, withhold or permanently discontinue pembrolizumab and administer corticosteroids. Dose modification and toxicity management guidelines for irAEs associated with pembrolizumab/combination treatment are provided in Table 7.

Attribution of Toxicity:

When study interventions are administered in combination, attribution of an adverse event to a single component is likely to be difficult. Therefore, while the investigator may attribute a toxicity event to the combination, to mirvetuximab soravtansine alone or to pembrolizumab alone, for adverse events listed in Table 7, both interventions must be held according to the criteria in Table 7 Dose Modification Guidelines for Pembrolizumab.

Holding Study Interventions:

When study interventions are administered in combination, if the AE is considered immune-related, both interventions should be held according to recommended dose modifications.

Restarting Study Interventions:

Participants may not have any dose modifications (no change in dose or schedule) of pembrolizumab in this study, as described in Table 7.

- If the toxicity does not resolve or the criteria for resuming treatment are not met, the participant must be discontinued from all study interventions.
- If the toxicities do not resolve and conditions are aligned with what is defined in Table 7, the combination of mirvetuximab soravtansine and pembrolizumab bay be restarted at the discretion of the investigator. In these cases where the toxicity if attributed to the combination or to mirvetuximab soravtansine alone, re-initiation of pembrolizumab as a monotherapy may be considered at the PI's discretion.

Table 7: Dose Modification Guidelines for Pembrolizumab

Instructions:

1. Corticosteroid taper should be initiated upon AE improving to Grade 1 or less and continue to taper over at least 4 weeks.
2. For situations where pembrolizumab has been withheld, pembrolizumab can be resumed after AE has been reduced to Grade 1 or 0 and corticosteroid has been tapered. Pembrolizumab should be permanently discontinued if AE does not resolve within 12 weeks of last dose or corticosteroids cannot be reduced to ≤10 mg prednisone or equivalent per day within 12 weeks.
3. For severe and life-threatening irAEs, IV corticosteroid should be initiated first followed by oral steroid. Other immunosuppressive treatment should be initiated if irAEs cannot be controlled by corticosteroids.

Immune-related AEs	Toxicity grade or conditions (CTCAEv5.0)	Action taken to pembrolizumab	irAE management with corticosteroid and/or other therapies	Monitor and follow-up
Pneumonitis	See Table 4	See Table 4	See Table 4	See Table 4

Diarrhea / Colitis	Grade 2 or 3	Withhold	• Administer corticosteroids (initial dose of 1-2 mg/kg prednisone or equivalent) followed by taper	• Monitor participants for signs and symptoms of enterocolitis (ie, diarrhea, abdominal pain, blood or mucus in stool with or without fever) and of bowel perforation (ie, peritoneal signs and ileus).<br>• Participants with ≥ Grade 2 diarrhea suspecting colitis should consider GI consultation and performing endoscopy to rule out colitis.<br>• Participants with diarrhea/colitis should be advised to drink liberal quantities of clear fluids. If sufficient oral fluid intake is not feasible, fluid and electrolytes should be substituted via IV infusion.
	Grade 4	Permanently discontinue		
AST / ALT elevation or Increased bilirubin	Grade 2	Withhold	• Administer corticosteroids (initial dose of 0.5- 1 mg/kg prednisone or equivalent) followed by taper	• Monitor with liver function tests (consider weekly or more frequently until liver enzyme value returned to baseline or is stable
	Grade 3 or 4	Permanently discontinue	• Administer corticosteroids (initial dose of 1-2 mg/kg prednisone or equivalent)	

			followed by taper	
Type 1 diabetes mellitus (T1DM) or Hyperglycemia	Newly onset T1DM or<br>Grade 3 or 4 hyperglycemia associated with evidence of β-cell failure	Withhold	• Initiate insulin replacement therapy for participants with T1DM<br>• Administer anti-hyperglycemic in participants with hyperglycemia	• Monitor participants for hyperglycemia or other signs and symptoms of diabetes.
Hypophysitis	Grade 2	Withhold	• Administer corticosteroids and initiate hormonal replacements as clinically indicated.	• Monitor for signs and symptoms of hypophysitis (including hypopituitarism and adrenal insufficiency)
	Grade 3 or 4	Withhold or permanently discontinue[1]		
Hyperthyroidism	Grade 2	Continue	• Treat with non-selective beta-blockers (eg, propranolol) or thionamides as appropriate	• Monitor for signs and symptoms of thyroid disorders.
	Grade 3 or 4	Withhold or permanently discontinue[1]		
Hypothyroidism	Grade 2-4	Continue	• Initiate thyroid replacement hormones (eg, levothyroxine or liothyroinine) per standard of care	• Monitor for signs and symptoms of thyroid disorders.
Nephritis and Renal dysfunction	Grade 2	Withhold	• Administer corticosteroids (prednisone 1-2 mg/kg or equivalent) followed by taper.	• Monitor changes of renal function
	Grade 3 or 4	Permanently discontinue		
Neurological Toxicities	Grade 3 or 4	Permanently discontinue	• Based on severity of AE administer corticosteroids	• Ensure adequate evaluation to confirm etiology and/or exclude other causes
Myocarditis	Grade 1	Withhold	• Based on severity of AE administer corticosteroids	• Ensure adequate evaluation to confirm etiology and/or exclude other
	Grade 2, 3 or 4	Permanently discontinue		

				causes
Exfoliative Dermatologic Conditions	Suspected SJS, TEN, or DRESS	Withhold	• Based on severity of AE administer corticosteroids	• Ensure adequate evaluation to confirm etiology or exclude other causes
	Confirmed SJS, TEN, or DRESS	Permanently discontinue		
All other immune-related AEs	Intolerable/ persistent Grade 2	Withhold	• Based on type and severity of AE administer corticosteroids	• Ensure adequate evaluation to confirm etiology and/or exclude other causes
	Grade 3	Withhold or discontinue based on the type of event. Events that require discontinuation include and not limited to: Gullain-Barre Syndrome, encephalitis		
	Grade 4 or recurrent Grade 3	Permanently discontinue		
1. Withhold or permanently discontinue pembrolizumab is at the discretion of the investigator or treating physician.<br>**NOTE:**<br>• For participants with Grade 3 or 4 immune-related endocrinopathy where withhold of pembrolizumab is required, pembrolizumab may be resumed when AE resolves to ≤ Grade 2 and is controlled with hormonal replacement therapy or achieved metabolic control (in case of T1DM).				

Pembrolizumab may be interrupted for situations other than treatment-related AEs such as medical / surgical events or logistical reasons not related to study therapy. Participants should be placed back on study therapy within 3 weeks of the scheduled interruption, unless otherwise discussed with the Overall PI. The reason for interruption should be documented in the patient's study record.

6.3 Re-treatment Criteria

For a patient to begin a new cycle or continue a cycle of therapy after a dose modification, the following criteria must be met.

- Pre-treatment criteria as described in Section 5.2.2 with the following exceptions
  - Treatment-emergent ocular disorders, which must have recovered to ≤ Grade 1 or baseline
  - Treatment-emergent pneumonitis which must have recovered to ≤ Grade 1

If the patient does not meet these criteria, dosing with the two agents in the combination regimen will be delayed and the patient should be re-evaluated within 48-72 hours. Dosing with both agents may resume if these criteria have been met. If the adverse event can be attributed to one of the agents in the combination regimen, that agent will be held or discontinued as per the Dose Modification Guidelines

described in Sections 6.1 and 6.2 respectively; however, treatment with the second agent may resume provided all the other criteria are met. However, if the next cycle is delayed due to treatment-related toxicity longer than 12 weeks then the patient should be removed from study treatment. In such cases, continuation of study treatment may be considered for those patients who have experienced clinical benefit if agreed upon between the Overall PI and the Investigator.

ADVERSE EVENTS: LIST AND REPORTING REQUIREMENTS

Adverse event (AE) monitoring and reporting is a routine part of every clinical trial. The following list of reported and/or potential AEs (Section 7.1) and the characteristics of an observed AE (Section 7.2) will determine whether the event requires expedited reporting **in addition** to routine reporting.

7.1 Expected Toxicities

7.1.1 Adverse Events List(s)

7.1.1.1 Adverse Event List(s) for Mirvetuximab Soravtansine (IMGN853)

For the purposes of regulatory reporting the events listed in Table 8 are considered expected for mirvetuximab soravtansine.

Table 8: Mirvetuximab Soravtansine Adverse Drug Reactions (ADR)

MedDRA SOC	SARs	Number of Patients Exposed N = 404		
		All SARs	Occurrence of Fatal SARs	Occurrence of Life-threatening SARs
		n (%)	n (%)	n (%)
**Gastrointestinal disorders**	Diarrhea ≤ Grade 3	137 (34)	0	0
	Nausea ≤ Grade 3	167 (41)	0	0
	Vomiting ≤ Grade 3	64 (16)	0	0
**General disorders and site conditions**	Fatigue ≤ Grade 3	129 (32)	0	0
	Asthenia ≤ Grade 3	45 (11)	0	0
**Metabolism and nutrition disorders**	Dehydration ≤ Grade 3	11 (3)	0	0
**Respiratory, thoracic and mediastinal disorders**	Pneumonitis ≤ Grade 2	34 (8)	0	0

Abbreviations: IB = Investigator Brochure; MedDRA = Medical Dictionary for Regulatory Activities; MIRV = mirvetuximab soravtansine; SAR = serious adverse reaction; SOC = system organ class.

Additional detailed information can be found in the Investigators Brochure.

7.1.1.2 Adverse Event List(s) for Pembrolizumab

Detailed information on adverse events associated with pembrolizumab can be found in the Investigators Brochure.

7.2 Adverse Event Characteristics

- **CTCAE term (AE description) and grade:** The descriptions and grading scales found in the revised NCI Common Terminology Criteria for Adverse Events (CTCAE) version 5.0 will be utilized for AE reporting. All appropriate treatment areas should have access to a copy of the CTCAE version 5.0. A copy of the CTCAE version 5.0 can be downloaded from the CTEP web site http://ctep.cancer.gov/protocolDevelopment/electronic_applications/ctc.htm.

- **For expedited reporting purposes only:**
  - AEs for the agent(s) that are listed above should be reported only if the adverse event varies in nature, intensity or frequency from the expected toxicity information which is provided.

- **Attribution** of the AE:
  - Definite – The AE *is clearly related* to the study treatment.
  - Probable – The AE *is likely related* to the study treatment.
  - Possible – The AE *may be related* to the study treatment.
  - Unlikely – The AE *is doubtfully related* to the study treatment.
  - Unrelated – The AE *is clearly NOT related* to the study treatment.

7.3 Serious Adverse Events

A serious adverse event (SAE) is any AE occurring at any dose that occurs after the initial dose of study treatment, during treatment, or within 30 days of the last dose of treatment that results in any of the following outcomes:

- Death
- Is life-threatening
- Requires inpatient hospitalization $\geq$ 24 hours
- Requires prolongation of existing hospitalization
- A persistent or significant disability or incapacity or substantial disruption of the ability to conduct normal life functions
- A congenital anomaly or birth defect

Important medical events that may not result in death, be life-threatening, or require hospitalization may be considered an SAE when, based upon appropriate medical judgment, they may jeopardize the patient and may require medical or surgical intervention to prevent one of the outcomes listed in this definition. Examples of such medical events include allergic bronchospasm requiring intensive treatment in an emergency room or at home, blood dyscrasias or convulsions that do not result in inpatient hospitalization.

Note that hospitalization is defined as admission to treat a clinical adverse event.

The following events would not be considered hospitalizations for SAE reporting purposes:

- <24-hour hold for observation, admission to a hospice facility or nursing home, respite

care, outpatient surgery, social admission (e.g., a homeless patient) or admission not associated with a precipitating clinical adverse event (e.g., elective or pre-planned surgery, or in-patient administration of subsequent chemotherapy, etc.).

- Events of progression of the patient's underlying cancer as well as events clearly related to progression of the patient's cancer (signs and symptoms of progression) should not be reported as a serious adverse event unless the outcome is fatal within the safety reporting period. If the event has a fatal outcome within the safety reporting period, then the event of Progression of Disease must be recorded as an important medical event and as a SAE with CTC Grade 5 (fatal outcome) indicated.

Note: In addition to the above criteria, adverse events meeting either of the below criteria, although not serious per ICH definition, are reportable to ImmunoGen and Merck in the same timeframe as SAEs to meet certain local requirements. Therefore, these events are considered serious for collection purposes.

- Is a new cancer (that is not a condition of the study);
- Is associated with an overdose

7.4 Adverse Event Reporting to Overall PI

7.4.1 In the event of an unanticipated problem or life-threatening complications treating investigators must immediately notify the Overall PI.

7.4.2 Investigators **must** report within 1 business day of investigator awareness to the Overall PI any serious adverse event (SAE) that occurs from the time that the subject provides informed consent, through and including the 30 calendar days after the last administration of study treatment.

7.4.3 Adverse Event Reporting Guidelines

All participating sites will report AEs to the Sponsor-Investigator per DF/HCC requirements, and the IRB of record for each site as applicable per IRB policies. The table below indicates which events must be reported to the Overall PI.

Table 9: DF/HCC Reportable Adverse Events (AEs)

Attribution	DF/HCC Reportable Adverse Events(AEs)				
	**Gr. 2 & 3 AE Expected**	**Gr. 2 & 3 AE Unexpected**	**Gr. 4 AE Expected**	**Gr. 4 AE Unexpected**	**Gr. 5 AE Expected or Unexpected**
Unrelated Unlikely	Not required	Not required	5 calendar days#	5 calendar days	24 hours*
Possible Probable Definite	Not required	5 calendar days	5 calendar days#	5 calendar days	24 hours*

# If listed in protocol as expected and not requiring expedited reporting, event does not need to be reported.

* For participants enrolled and actively participating in the study ***or*** for AEs occurring within 30 days of the last intervention, the AE should be reported within <u>1 business day</u> of learning of the event.

7.5 Reporting Period

For the time period beginning when the consent form is signed until 30 days following cessation of treatment, any serious adverse event, or follow up to a serious adverse event, including death due to any cause whether or not related to the study products, must be reported within 24 hours to the Overall PI.

All participants with serious adverse events must be followed up for outcome.

7.6 Adverse Event Reporting to ImmunoGen

The following events will be reported to ImmunoGen:

- Any serious adverse event (SAE), considered by an investigator who is a qualified physician to be related to mirvetuximab soravtansine (Section 7.6.1)
- Any new cancers or overdose of mirvetuximab soravtansine (Section 7.6.1)
- Pregnancy or Lactation (Section 7.6.2)
- Events of Special/Clinical Interest (Section 7.6.3)

7.6.1 Serious Adverse Event Reporting to ImmunoGen

For SAE reporting, including those that are expedited, the MedWatch (MW) format is preferred. A line listing is acceptable for non-expedited cases and should be sent within 2 working days to ImmunoGen using: IMGNPVGSAFETY@immunogen.com (CC).

IND Safety Reports (final MedWatch only) must be sent, upon submission to FDA, to: IMGNPVGSAFETY@immunogen.com
In addition to SAE, any new cancer (that is not a condition of the study) or overdose of mirvetuximab soravtansine will be reported to ImmunoGen the same timeframe as SAEs using the procedure for SAEs.

7.6.2 Reporting a Pregnancy and Lactation to ImmunoGen

Pregnancy and lactation are exclusion criteria. The Overall PI and ImmunoGen must be immediately notified (within 24 hours) in the event of a pregnancy occurring during the course of the study and through 120 days after a patient's last dose of mirvetuximab soravtansine and through 30 days following cessation of study treatment if the subject initiates new anticancer therapy, whichever is earlier. Pregnancy is not to be reported as an AE; the pregnancy report form should be used to report a pregnancy. All reported pregnancies must be followed to the completion/termination of the pregnancy. Pregnancy outcomes of spontaneous abortion, missed abortion, benign hydatidiform mole, blighted ovum, fetal death, intrauterine death, miscarriage and stillbirth must be reported as serious events (Important Medical Events). The reporting procedures for SAE will be followed. Pregnancy outcomes listed above are also reportable to ImmunoGen in the same timeframe as SAEs. If the pregnancy continues to term, the outcome

(health of infant) must also be reported.

7.6.3 Reporting Adverse Events of Special Interest (AESI) to ImmunoGen

An adverse event of special interest (AESI) (serious or non-serious) is one of scientific and medical concern specific to the ImmunoGen product for which ongoing monitoring and rapid communication by the investigator to ImmunoGen could be appropriate. These are reportable to ImmunoGen in the same timeframe as SAEs.

Mirvetuximab soravtansine:

- AESI: pneumonitis.

Any symptom or sign potentially suggestive of pneumonitis should be promptly reported to ImmunoGen as for SAE, even if non-serious and/or unrelated to mirvetuximab soravtansine. If it meets requirements for expedited reporting it will be reported to the IRB and FDA per guidelines as well. These AESIs are to be reported to ImmunoGen expeditiously within 48 hours of knowledge of the event, during the study through 30 days after receiving the last dose of study treatment. It is important that the investigator provide an assessment of relationship of the SAE or AESI to study treatment at the time of the initial report. The Sponsor-Investigator's study-specific Serious Adverse Event (SAE)/Adverse Events of Special Interest (AESI) Report Form (MW) must be used for reporting SAEs and AESIs. The contact information for reporting of SAEs and AESIs can be found on the SAE/AESI Reporting Form and Pregnancy Report Forms.

Radiologic findings suggestive of pneumonitis include new onset of any of the following:

- Pulmonary consolidation
- Pulmonary infiltrate
- Reticular infiltrate
- Nodular infiltrate
- Reticulo-nodular infiltrate
- Ground-glass pulmonary infiltrate
- Increased interstitial markings
- Interstitial infiltrate
- Honeycomb's appearance

7.7 Adverse Event Reporting to Merck

The following events will be reported to Merck & Co., Inc.:

- Any serious adverse event (SAE), considered by an investigator who is a qualified physician to be related to pembrolizumab (Section 7.7.1)
- Any new cancers (Section 7.7.1) or overdose of pembrolizumab (Section 7.7.2)
- Pregnancy and Lactation (Section 7.7.3)
- Events of Special/Clinical Interest (Section 7.7.4)

7.7.1 Serious Adverse Event Reporting to Merck

Any serious adverse event, considered by an investigator who is a qualified physician to be related to Merck product that is brought to the attention of the investigator at any time following consent through the end of the specified safety follow-up period specified in the paragraph above, or at any time outside of the time period specified in the previous paragraph also must be reported immediately to Merck Global Safety.

SAE reports and any other relevant safety information are to be reported within 24 hours to the Overall PI and forwarded within 2 working days to Merck Global Safety (Attn: Worldwide Product Safety; FAX 215-661-6229). Following submission to Merck Global Safety, forward the SAE report and a copy of the fax confirmation to .

In addition to SAE, any new cancer (that is not a condition of the study) will be reported to Merck within the same timeframe as SAEs, using the procedure for SAEs.

7.7.2 Reporting of Pembrolizumab Overdose to Merck

For purposes of this study, an overdose of pembrolizumab will be defined as any dose of 1,000 mg or greater (≥5 times the indicated dose). No specific information is available on the treatment of overdose of pembrolizumab. In the event of overdose, the participant should be observed closely for signs of toxicity. Appropriate supportive treatment should be provided if clinically indicated.

If an adverse event(s) is associated with ("results from") the overdose of a Merck product, the adverse event(s) is reported as a serious adverse event, even if no other seriousness criteria are met.

If a dose of Merck's product meeting the protocol definition of overdose is taken without any associated clinical symptoms or abnormal laboratory results, the overdose is reported to Merck as a non-serious Event of Clinical Interest (ECI), using the terminology "accidental or intentional overdose without adverse effect."

All reports of overdose with and without an adverse event must be reported within 24 hours to the Overall PI and within 2 working days hours to Merck Global Safety. (Attn: Worldwide Product Safety; FAX 215-661-6229)

7.7.3 Reporting of Pregnancy and Lactation to Merck

Although pregnancy and infant exposure during breast-feeding are not considered adverse events, it is the responsibility of investigators or their designees to report any pregnancy or lactation in a participant (spontaneously reported to them) that occurs during the study.

Pregnancies and infant exposures during breastfeeding that occur after the consent form is signed but before treatment allocation/randomization must be reported by the investigator if they cause the participant to be excluded from the trial, or are the result of a protocol-specified intervention, including but not limited to washout or discontinuation of usual therapy, diet, placebo treatment or a procedure.

Pregnancies and infant exposures during breastfeeding that occur from the time of treatment allocation/randomization through 120 days following cessation of pembrolizumab, or 30 days following cessation of treatment if the participant initiates new anticancer therapy, whichever is earlier, must be reported by the investigator. All reported pregnancies must be followed to the completion/termination of the pregnancy. Pregnancy outcomes of spontaneous abortion, missed abortion, benign hydatidiform mole, blighted ovum, fetal death, intrauterine death, miscarriage and stillbirth must be reported as serious events (Important Medical Events). If the pregnancy continues to term, the outcome (health of infant) must also be reported.

Such events must be reported within 24 hours to the Overall PI and within 2 working days to Merck Global Safety. (Attn: Worldwide Product Safety; FAX 215-661-6229)

7.7.4 Reporting Events of Clinical Interest to Merck

Selected non-serious and serious adverse events are also known as Events of Clinical Interest (ECI) and must be reported within 2 working days to Merck Global Safety. (Attn: Worldwide Product Safety; FAX 215-661-6229).

For the time period beginning when the consent form is signed until treatment allocation/randomization, any ECI, or follow up to an ECI, that occurs to any participant must be reported within 2 working days to Merck Global Safety if it causes the participant to be excluded from the trial, or is the result of a protocol-specified intervention, including but not limited to washout or discontinuation of usual therapy, diet, placebo treatment or a procedure.

For the time period beginning at treatment allocation/randomization through 90 days following cessation of treatment, or 30 days following cessation of treatment if the participant initiates new anticancer therapy, whichever is earlier, any ECI, or follow up to an ECI, whether or not related to Merck product, must be reported within 2 working days to Merck Global Safety.

Events of clinical interest for this trial include:

1. an overdose of Merck product, as defined in Section 7.7.2, that is not associated with clinical symptoms or abnormal laboratory results.
2. an elevated AST or ALT lab value that is greater than or equal to 3X the upper limit of normal and an elevated total bilirubin lab value that is greater than or equal to 2X the upper limit of normal and, at the same time, an alkaline phosphatase lab value that is less than 2X the upper limit of normal, as determined by way of protocol-specified laboratory testing or unscheduled laboratory testing,* whether or not related to Merck product

*Note: These criteria are based upon available regulatory guidance documents. The purpose of the criteria is to specify a threshold of abnormal hepatic tests that may require an additional evaluation for an underlying etiology.

7.8 Reporting to the Food and Drug Administration (FDA)

The Overall PI, as study sponsor, will be responsible for all communications with the FDA. The Overall PI will report to the FDA, regardless of the site of occurrence, any serious adverse event that meets the FDA's criteria for expedited reporting following the reporting requirements and timelines set by the FDA.

7.9 Reporting to Hospital Risk Management

Participating investigators will report to their local Risk Management office any participant safety reports, sentinel events or unanticipated problems that require reporting per institutional policy.

7.10 Routine Adverse Event Reporting

All Adverse Events **must** be reported in routine study data submissions to the Overall PI on the toxicity case report forms. **AEs reported through expedited processes (e.g., reported to the IRB, FDA, etc.) must <u>also</u> be reported in routine study data submissions.**

PHARMACEUTICAL INFORMATION

A list of the adverse events and potential risks associated with the investigational or other agents administered in this study can be found in Section 7.1.

8.1 Mirvetuximab Soravtansine (IMGN 853)

8.1.1 Description

Mirvetuximab soravtansine is an antibody-drug conjugate designed to target FRα. It consists of the humanized anti-FRα monoclonal antibody M9346A attached via a cleavable disulfide linker to the cytotoxic maytansinoid, DM4 (Figure 1).

DM4 is ~2% by weight relative to monoclonal antibody.

Due to the nature of the conjugation process, the number of DM4 molecules attached to the monoclonal antibody ranges from 1 to 7 molecules per antibody, with an average of 3 or 4 DM4 molecules per antibody. Conjugation of the maytansinoid to the tumor-targeting antibody ensures that the cytotoxic component remains inactive in the circulation. Release of the cytotoxic payload requires binding, internalization, and degradation of the antibody. The released payload then kills the cell by inducing G2-M arrest and cell death. Cellular processing of maytansinoid conjugates can also generate lipophilic catabolites that cross cell membranes and kill neighboring cells.

Figure 1: Mirvetuximab soravtansine

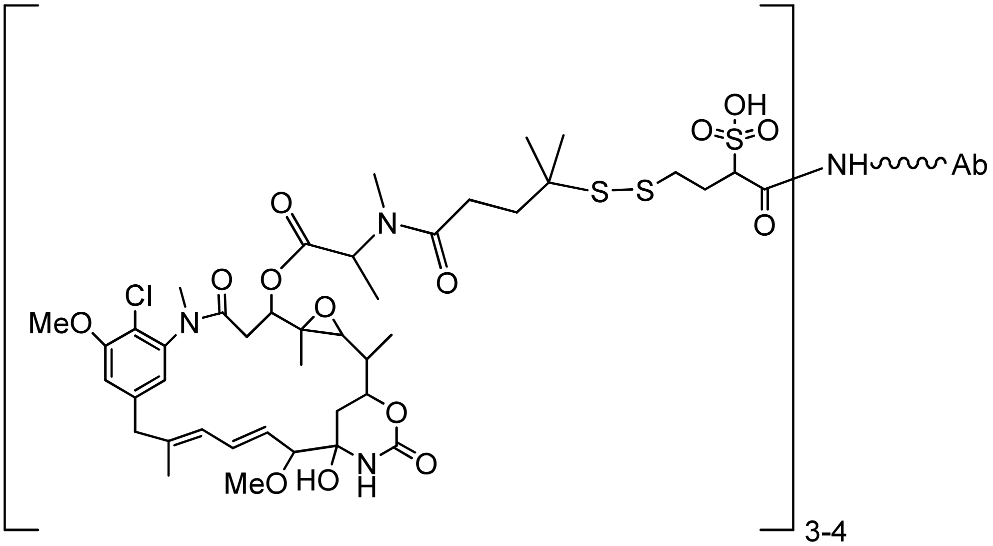

PK studies demonstrated the stability of mirvetuximab soravtansine in circulation, showed clearance via a distribution phase lasting about 24 hours followed by a slower terminal elimination phase after intravenous administration, and suggested linear PK.

8.1.2 Form

The investigational study drug, mirvetuximab soravtansine, will be provided by ImmunoGen, Inc. at a protein concentration of 5.0 mg/mL in an aqueous pH 5.0 buffered solution. IMGN853 will be available in a 20 mL Type I glass vial with 20 mL deliverable volume. The container closure for the Type I glass vials will consist of a 20 mm ETFE-coated serum stopper (Flurotec®) on the top and product contact surface with a 20 mm aluminum TruEdge® seal with blue Flip-off® top. Refer to the Pharmacy Manual for labeling information.

8.1.3 Storage and Stability

Mirvetuximab soravtansine should be stored upright in the carton supplied, protected from light, refrigerated at 2-8°C (36 to 46°F) until the time of preparation and should not be shaken or frozen.

Table 3 specifies the cumulative allowable time limits for specific temperature ranges and reporting requirements for IMGN853 while in storage. The total cumulative temperature excursion time for vials must be documented by the pharmacy in the applicable drug accountability logs.

Table 1: IMGN853 Storage, Temperature Excursion Allowance, and Reporting Requirements

**Temperature Range**	**Time Duration**	**Allowable?**	**Comments**
< -5.4°C	Any	No	Investigative sites to quarantine product

			and report excursion to sponsor-investigator.
-5.4 to 1.4°C	Up to 24 hours (1,440 minutes) cumulative	Yes	IMGN853 can be used<br>Investigative sites to check cumulative time. If less than or equal to 24 hours (1,440 minutes) then no reporting required.
1.5 to 8.4°C	Any	Yes	IMGN853 can be used.
8.5 to 25.4°C	More than 6 hours (360 minutes) cumulative	Yes	IMGN853 can be used.<br>Investigative sites to check cumulative time. If less than or equal to 6 hours (360 minutes) then no reporting required.
8.5 to 25.4°C	More than 6 hours (360 minutes) cumulative	No	Investigative sites to quarantine product and report excursion and cumulative time to sponsor-investigator.<br>*Use may be permitted upon review.
> 25.4°C	Any	No	Investigative sites to quarantine product and report excursion to sponsor-investigator.

Note: The temperature excursion allowances for onsite storage of IMGN853 do not apply during the shipment period. Any excursion outside of 2 to 8ºC (36 to 46 ºF) experienced during transit must be reported.

8.1.4 Compatibility

IMGN853 is NOT compatible with saline (0.9% sodium chloride). Therefore, dilutions must be made in 5% dextrose and 5% D-glucose. Other diluents have not been tested, and therefore, cannot be deemed compatible.

Any combination of the materials used in the fluid contact pathways (e.g., infusion bag, syringe, needle, infusion set, closed system transfer device, filter, or catheter materials) listed below are acceptable for use:

- Acetyltributylcitrate (ATBC)
- Acrylonitrile-butadiene-styrene copolymer (ABS)
- Diethylhydroxylamine (DEHA)
- Diisononyl ester (DINCH)
- Dioctyl terephthalate (DEHT or DOTP)
- Ethylene Vinyl Acetate (EVA)
- Fluorinated ethylene propylene (FEP)
- Polyacetal
- Polycarbonate
- Polyethersulfone (PES)

- Polyethylene
- Polyisoprene
- Polyolefin (e.g., polymer of ethylene and/or propylene)
- Polypropylene (PP)
- Polytetrafluoroethylene (PTFE), Acrylic
- Polytetrafluoroethylene (PTFE)
- Polyurethane (PUR)
- Polyvinyl chloride (PVC) plasticized with Di-2-ethyl-hexylphthalate (DEHP)
- Polyvinyl chloride (PVC) plasticized without Di-2-ethyl-hexylphthalate (non- DEHP)
- Silicone
- Stainless steel
- Thermoplastic elastomer (TPE)
- Trioctyl Trimellitate (TOTM or TETM)

Contact Sponsor-Investigator for materials not listed above.

8.1.5 Handling

Qualified personnel, familiar with procedures that minimize undue exposure to themselves and the environment, should undertake the preparation, handling, and safe disposal of the chemotherapeutic agent in a self-contained and protective environment.

8.1.6 Availability

Each site is responsible for requesting the investigational products from ImmunoGen.

8.1.7 Preparation

The total dose of IMGN853 will be based upon the adjusted ideal body weight (AIBW) using the following formula:

Adjusted Ideal Body Weight (AIBW)

$$IBW^1 + 0.4\ (\text{Actual weight} - IBW^1)$$

Where:

Ideal Body Weight (IBW)

$$IBW^1\ (\text{male}) = 0.9H^1\text{-}88$$

$$IBW^1\ (\text{female}) = 0.9H^1\text{-}92$$

(^{1}H=height in cm; W=weight in kg)

The patient's weight will be collected at each cycle. Dosing will be based off the wight at baseline prior to C1D1 unless current weight is > 10% different (not influenced by weight gain

or loss attributed to fluid retention). If required per institutional policy, it is acceptable to recalculate AIBW at each cycle using the patient's current weight. Sites must clearly document the weight used to calculate dose at each cycle.

Prior to preparation, the lot expiry date should be verified via the Certificate of Analysis (CoA) or label. A visual inspection for cracks, leaks or any other damage should be done before use and any damage must be reported to the Sponsor-Investigator and ImmunoGen at IMGNdrugresupply@immunogen.com.

Infusion bags must be labeled with the protocol number, subject number (patient ID), storage temperature, dose, and volume of IMGN853 in the bag, or according to Institutional protocol.

If necessary, study drug from different drug lots may be mixed in a single-dose administration.

- Remove the required number of vials from the refrigerator 25 minutes before preparation to allow IMGN853 solution for injection to reach ambient temperature (18 to 25°C)
- Keep IMGN853 vials protected from direct light
- Gently invert vials (without shaking) 8 to 10 times before withdrawing the drug
- Withdraw the required volume aseptically using an appropriate syringe and needle or CSTD (closed system transfer device)
- Choose a suitable infusion bag based on final volume for infusion.
- Dilute the drug with 5% dextrose or 5% D-glucose to a final concentration of 1 mg/mL to 2 mg/mL
    - **Important:** IMGN853 is **NOT compatible with saline** (0.9% sodium chloride). Therefore, dilutions must be made in 5% dextrose or 5% D-glucose.
- IMGN853 does not contain a preservative. Prepared study drug:
    - May be stored at room temperature but infusion must be completed within 8 hours after preparation
    - May be stored at 2 to 8°C for up to 24 hours, and once removed from the fridge it may be stored at room temperature, but the infusion must be completed within 8 hours
- If the prepared study drug is stored at 2 to 8 °C, it should be removed from the refrigerator at least 30 to 60 minutes prior to administration, to allow the solution to reach room temperature. Allow the prepared study drug to reach room temperature without warming methods.
- The total time at room temperature may not exceed 8 hours, inclusive of infusion time. Light protection is not required for prepared IMGN853. Ensure that in-line filter of 0.2 or 0.22 µm is used to filter diluted IMGN853 from the bag during administration

8.1.8 Administration

During combination treatment, mirvetuximab soravtansine should be administered first, followed by pembrolizumab.

- IMGN853 should be administered IV, using an IV tubing administration set with a 0.2 µm or 0.22 µm in-line filter.
- The infusion tubing may be primed with 5% dextrose (or 5% D-glucose) or may be primed with IMGN853, ensuring that no drug is wasted in the process.
- Following the infusion, the IV line should be flushed with 5% dextrose, or 5% D-glucose as required to ensure delivery of the full dose.

Infusion Rate: Initially IMGN853 should be administered at a rate of 1 mg/min; after a continuous 30 minutes of infusion, the rate can be increased to 3 mg/min if well tolerated. If well tolerated after a continuous 30 minutes of infusion at 3 mg/min the rate may be increased to 5 mg/min. Subsequent infusions may be delivered at the highest tolerated rate.

Duration of infusion: The overall length of infusion will vary depending on dose and patient tolerability. Patients will remain in the clinic under observation for 2 hours after the first infusion, and for at least 1 hour after each subsequent infusion of MIRV, during which pembrolizumab infusion can be administered. While in the treatment area, patients are closely monitored for AEs.

Details of the dose level and total dose administered should be documented in the source records as noted below:

- Start and stop times for each administration (end of infusion should be the time of completion of the post-dose 5% dextrose or 5% D-glucose flush)
- Infusion rate(s)
- Total calculated dose (in mg)
- Total calculated volume (in mL)
- Total volume administered (in mL)
- Times for any interruptions
- Reason if any administration is stopped prematurely or interrupted and volume administered prior to interruption.

8.1.9 Ordering

Investigative sites will order their own supply of mirvetuximab soravtansine.

8.1.10 Accountability

The investigator, or a responsible party designated by the investigator, should maintain a careful record of the inventory and disposition of the agent using the NCI Drug Accountability Record Form (DARF) or another comparable drug accountability form. (See the NCI Investigator's Handbook for Procedures for Drug Accountability and Storage.)

8.1.11 Destruction and Return

Expired investigational product will be destroyed at the site per institutional policy. Upon completion or termination of the study, all unused and/or partially used investigational product will be destroyed at the site per institutional policy.

8.2 Pembrolizumab

8.2.1 Description

Pembrolizumab is a highly selective humanized mAb designed to block the interaction between PD-1 and its ligands, PD-L1 and PD-L2. Pembrolizumab is an IgG4/kappa isotype with a stabilizing sequence alteration in the Fc region. The theoretical molecular weights of the heavy and light chains derived from the amino acid sequences, excluding glycosylation, are 49.4 kiloDaltons (KDa) and 23.7 KDa, respectively. The antibody is heterogeneously glycosylated at asparagine 297 within theFc domain of each heavy chain, yielding a molecular weight of approximately 149 KDa for intact pembrolizumab.

8.2.2 Form

Pembrolizumab Solution for Infusion is a sterile, non-pyrogenic clear to slightly opalescent, colorless to slightly yellow aqueous solution supplied in single-use Type I glass vial containing 100 mg/ 4 mL of pembrolizumab. The drug product is manufactured using facilities and practices under Good Manufacturing Practice (GMP) requirements.

Pembrolizumab Solution for Infusion 100 mg/ 4 mL vial is a liquid DP (manufactured using the fully formulated DS with L-histidine as a buffering agent, polysorbate 80 as a surfactant, and sucrose as a stabilizer/tonicity modifier, and Water for Injection as Solvent). Pembrolizumab Solution for Infusion vials are filled to a target of 4.25mL (106.25mg) to ensure recovery of 4.0mL (100mg). At the point of use, Pembrolizumab Solution for Infusion is diluted with 0.9% Sodium Chloride Injection, USP (normal saline) or regional equivalent or 5% Dextrose Injection, USP (5% dextrose) or regional equivalent to 1-10 mg/mL before intravenous (IV) administration through an infusion filter.

8.2.3 Storage and Stability

Pembrolizumab Solution for Infusion 100 mg/ 4 mL vial should be stored under refrigerated conditions 2°C to 8°C (36-46 °F). Vials should be stored in the original box to ensure the drug product is protected from light. Do not shake and do not freeze.

Store the diluted solution from KEYTRUDA 100 mg/vial either

- At room temperature for no more than 6 hours from the time of dilution. This includes room temperature storage of the diluted solution, and the duration of infusion.
- Under refrigeration at 2°C to 8°C (36-46 °F) for no more than 96 hours from the time of dilution. If refrigerated, allow the diluted solution to come to room temperature prior to administration. Do not shake.

Discard after 6 hours at room temperature or after 96 hours under refrigeration.

Do not freeze.

Clinical supplies must be stored in a secure, limited-access location under the storage conditions specified on the label. Receipt and dispensing of trial medication must be recorded by an authorized person at the trial site. Clinical supplies may not be used for any other purpose other than that stated in the protocol.

8.2.4 Compatibility

Pembrolizumab should be prepared in 0.9% Sodium Chloride Injection, USP (normal saline) or regional equivalent or 5% Dextrose Injection, USP (5% dextrose) or regional equivalent. The final concentration of pembrolizumab in the infusion solutions should be between 1 mg/mL and 10 mg/mL. Pembrolizumab SHOULD **NOT** BE MIXED WITH OTHER DILUENTS.

See section 8.2.7 for infusion bag material compatibility.

8.2.5 Handling

Qualified personnel, familiar with procedures that minimize undue exposure to themselves and the environment, should undertake the preparation, handling, and safe disposal of the chemotherapeutic agent in a self-contained and protective environment.

8.2.6 Availability

Each site is responsible for requesting the investigational products from Merck.

8.2.7 Preparation

Aseptic technique must be strictly observed throughout the preparation procedure. Use of a biosafety cabinet is preferred since no anti-microbial preservative is present in the product; however, it is not mandatory unless specified by site standard operating procedure.

The preferred method of dose preparation is the volumetric method. The Sponsor recommends reconstitution and administration of pembrolizumab that follows the parameters in the Pharmacy Manual, however if use of gravimetric preparation is mandatory due to local site procedures, the following requirements must be satisfied and documented:

- Draw the required volume up to 4.0 mL (100 mg) of pembrolizumab from each vial
- Limit the number of punctures of each vial to one
- For gravimetric preparation method using density of pembrolizumab solution, a value of 1.03 g/mL should be used

Use of spikes or other CSTDs (closed system transfer devices) are permitted as long as the pharmacist is aware of the hold-up volume (volume of fluid that is left in the device) and that the contact time is ≤ 30 min. HQ approval is only needed for spikes/ CSTDs with contact time

greater than 30 min to ensure compatibility of the device. Please note that as long as the CSTD or spike does not come into contact with the drug product during infusion, then 30 mins does not count in infusion time.

Sites may choose to select an infusion bag size based on their institutional practice provided the following conditions are met:

- Concentration of pembrolizumab is between 1 mg/mL and 10 mg/mL
- The infusion volume to bag capacity ratio should not be less than 0.3. In other words, the bag must be filled to at least 30% capacity.

A suitable infusion bag material must be chosen. The bag may be empty, or it may contain normal saline. The following infusion bag materials are compatible with pembrolizumab:

- PVC plasticized with DEHP
- PVC, DEHP free
    - o Since it is still a PVC set, DEHP must be replaced with another plasticizer. If the alternate plasticizer is DEHT or TOTM then it is ok.
- Non-PVC (polyolefin)
- Ethylene-vinyl acetate (EVA)
- Polyethylene (PE) lined polyolefin
- Polypropylene (PP)
- Low density poly ethylene (LDPE)
- Clear Polyurethane (PVC Free, DEHP Free)
- Acrylic
- Polymethylmethacrylate,
- Silicone rubber with Fluorosilicone
- Polyethylene terephthalate (PET)
- Stainless steel
- Polybutadiene
- Polycarbonate (PC)
- Isoprene rubber
- Polyvinyl chloride
- Polyethersulfone (membrane pore size 0.2µm) non-PVC multilayer co-extrusion infusion bag: three layers co-extrusion infusion bag; polypropylene/ polypropylene/ polypropylene
- Acrylonitrile butadiene styrene (ABS)
- Polytetrafluoroethylene
- Dioctyl terephthalate (DOTP)

*Contact Sponsor-Investigator for materials not listed above

To prepare the pembrolizumab infusion bag:

1. Calculate the volume of pembrolizumab and normal saline required to prepare the infusion (admixture) bag. The dose of pembrolizumab used in this trial is 200mg.
    - Volume of pembrolizumab (mL) = required dose amount (mg) / 25 (mg/mL)
    - Volume of normal saline = total infusion volume – volume of pembrolizumab from

   above
   - If a bag pre-filled with normal saline is being used, remove the excess volume of normal saline using a sterile syringe (Polypropylene, latex-free) attached to a suitable needle. Keep in consideration the excess bag fill volume as well as the volume of pembrolizumab to be added to the bag to prepare the infusion solution. This helps ensure that the concentration in the bag can be accurately calculated and falls within the acceptable range of 1 mg/mL to 10 mg/ml. If the site would like to proceed without removing excess saline they must ensure that the concentration of pembrolizumab would still fall within acceptable range.
   - If an empty bag is being used, withdraw the necessary volume of normal saline from another appropriate bag and inject into the empty bag. Keep in consideration the volume of pembrolizumab to be added to the bag to prepare the infusion solution.

2. Withdraw the required volume of pembrolizumab from the vial(s) (up to 4 mL from each vial) using a sterile syringe attached to a suitable needle. The vial(s) may need to be inverted to remove solution.

- Volume of pembrolizumab (mL) = required dose amount (mg) / 25 (mg/mL)
- Note: If it is necessary to use several vials, it is advisable to withdraw from several vials into a suitable size single use syringe using a new needle for each vial.
- Discard any unused portion left in the vial as the product contains no preservative.

3. Add the required pembrolizumab into the infusion IV bag containing normal saline and gently invert the bag 10-15 times to mix the solution.

- From a microbiological point of view, diluted solution should be used as soon as possible after preparation
- Pembrolizumab solutions may be stored at room temperature for a cumulative time of up to 6 hours. This includes room temperature storage of admixture solutions in the IV bags and the duration of infusion.
- IV bags may be stored under refrigeration at 2 °C to 8 °C (36 °F to 46 °F), total cumulative storage time at room temperature and refrigeration should not exceed 24 hours. **(96 hours is acceptable dependent upon country approved commercial Keytruda label).**

If the infusion bag is excessively handled or shaken, particulates may form. If this occurs, discard the bag and create a new bag taking care not to shake. Please contact your HQ clinical study team if particulates are noticed for further instructions. Be prepared to provide the following information:

- IV bag manufacture, lot and expiry
- Target volume of admixture solution in the IV bag (e.g. 100 mL, 200 mL etc.)
- Amount of drug product (mL or mg) added to the bag
- Drug product lot
- Brief description of the nature of visible particles (color, shape, size, numbers etc.).

To prevent the formation of particulates:

- Minimize agitation
- Ensure dilution of admixture solution is 1mg/mL to 10 mg/mL
- Minimization of headspace (empty space over the liquid) in syringes and admixture bags

- Avoid siliconized products during preparation (Please note there is particulate formation when there is over agitation of the product and when concentrations are less than 1 mg/mL)

If particles are seen in the vials, these will need to be reported as clinical supply complaints per GCD 104. If particles are seen in the admixture solution, these need to be reported to the HQ clinical study team for further consultation with the pharmacy manual owner.

8.2.8 Administration

See Section 5.3.2. During combination treatment, mirvetuximab soravtansine should be administered first, followed by pembrolizumab. Details of the dose level and total dose administered should be documented in the source records

Pembrolizumab administration will be monitored to ensure compliance and proper documentation of the infusion procedure as well as management of infusion reactions should they occur.

8.2.9 Ordering

Investigative sites will order their own supply of pembrolizumab.

8.2.10 Accountability

The investigator, or a responsible party designated by the investigator, should maintain a careful record of the inventory and disposition of the agent using the NCI Drug Accountability Record Form (DARF) or another comparable drug accountability form. (See the NCI Investigator's Handbook for Procedures for Drug Accountability and Storage.)

8.2.11 Destruction and Return

Expired investigational product will be destroyed at the site per institutional policy. Upon completion or termination of the study, all unused and/or partially used investigational product will be destroyed at the site per institutional policy.

BIOMARKER, CORRELATIVE, AND SPECIAL STUDIES

9.1 Biomarker Studies

9.1.1 FRα Expression - Integral Biomarker

All patients must submit archived or new biopsy tumor tissue, or formalin-fixed, paraffin embedded (FFPE) block of sufficient size to allow for sectioning of ten 5-micron tissue sections or five unstained slides for central analysis of FRα expression prior to enrollment.

The tumor samples will be analyzed centrally for FRα expression by Immunohistochemistry (IHC) in the Companion Diagnostics Pharma Services CAP-accredited and CLIA-certified laboratory and Pathology Services at Ventana Medical Systems, Inc.

Only patients with confirmation of FRα positivity by the Ventana FOLR2.1 IHC assay (Ventana Medical Systems. Tucson, AZ) will be eligible to enroll in the study.

Samples will be shipped Monday – Thursday to (address below):

CDx CAP/CLIA Laboratory
Ventana Medical Systems, Inc.
Attn: CDx CAP/CLIA Laboratory
1910 E. Innovation Park Drive
Tucson, AZ 85755

9.1.2 Microsatellite Stability – Integral Biomarker

Prior to enrollment, patients must have documented MSS by routine methods including MMR IHC, MSI PCR or MSI by Next Generation Sequencing (NGS). Only patients with intact expression of all four MMR proteins by IHC, or MSS by PCR or NGS from a CLIA-certified laboratory will be eligible to enroll.

9.2 Laboratory Correlative Studies (FFPE Tumor Tissue)

Correlative studies may be performed in the Immune Oncology and DNA Repair Centers here at DFCI or Ventana Medical Systems. Depending on funding availability these studies may include: i) determination of CD3+, CD8+ TILs, CD8+/CD4+FOXP3+ TIL ratio, CD137+CD8+ TILs, CD137+CD8+/CD4+FOXP3+ TIL ratio, peritumoral lymphocytes and correlation with response; ii) expression of immune checkpoints including TIM-3, LAG-3, CTLA-4, PD-L2, PD-L1, PD-1, IDO and correlation with response; iii) targeted next generation sequencing (OncoPanel) to determine specific genetic alterations and correlate with response. Where OncoPanel sequencing has already been performed on patient samples clinically or through DF/HCC protocols 11-104, 17-000, and 20-000, we may plan to access these results. Additionally, we will characterize the FOLR1 expression in uterine serous cancers and overlap with other potential targets of interest, which may include but is not limited to HER2 and Trop2.

If archival tumor tissue in the form of unstained slides are used for central analysis of FRα expression prior to enrollment, the remaining five unstained slides should be shipped to the DFCI coordinating center along with the manifest form provided to each site. Please send the samples to:

Sandy Elias
Dana-Farber Cancer Institute
450 Brookline Ave, DA-122
Boston, MA 02215
Email contact: Sandy_Elias@dfci.harvard.edu; Martin_Hayes@dfci.harvard.edu

STUDY CALENDAR

Baseline screening evaluations are to be conducted within 14 days prior to start of protocol therapy. Scans and x-rays must be done ≤4 weeks prior to the start of therapy. In the event that the participant's condition is deteriorating, laboratory evaluations should be repeated within 48 hours prior to initiation of the next cycle of therapy.

Assessments must be performed prior to administration of any study agent. Study assessments and agents should be administered within ± 3 days of the protocol-specified date, unless otherwise noted.

	Pre-Study	Cycle 1			Cycle 2 and beyond	Off-treatment visit[e]
		Day 1	Day 8	Day 15	Day 1	
Mirvetuximab Soravtansine		X			X	
Pembrolizumab		X			X	
Archival FFPE sample	X					
Collection of MSS documentation	X					
Demographics	X					
Medical history	X					
Concurrent medications	X	X--X				
Physical exam	X	X	X	X	X	X
Vital signs	X	X	X	X	X	X
Height	X					
Weight	X	X	X		X	X
Pulse Oximetry	X	X	X	X	X	
ECOG Performance status	X	X	X		X	X
CBC w/diff, plts	X	X	X	X	X	X
Serum chemistry[a]	X	X	X	X	X	X
TSH	X	X			X	X
PT/INR/PTT	X					X
EKG (as indicated)	X					X
Pulmonary Function Tests[c]	X					
Ophthalmic examinations[d]	X	Every other cycle (from time toxicity first reported, until resolved to baseline or ≤Grade 1)				
Schirmer Test[d]	X	For patients who experience treatment-emergent eye disorders, the Schirmer Test will be repeated at the first on-study ophthalmic examination and at subsequently if clinically indicated				
Ocular Symptom Assessment[d]	X	X			X	X
Adverse event evaluation		X--X				
Tumor measurements	X	Tumor measurements are repeated every 2 cycles (6 weeks, ± 1 week). Documentation (radiologic) must be provided for				

		participants removed from study for progressive disease.				
B-HCG	X[b]					
Lubricating artificial tears administration		Daily as directed[f]				X[g]
Corticosteroid eye drop administration		Six times daily on Days 1-4 and four times daily on Days 5-8 of each cycle[f]				X[g]

a. Albumin, alkaline phosphatase, total bilirubin, bicarbonate, BUN, calcium, chloride, creatinine, glucose, LDH, phosphorus, potassium, total protein, SGOT [AST], SGPT [ALT], sodium.
b. Serum pregnancy test (women of childbearing potential) should be done within 3 days of the first dose of study treatment.
c. Pulmonary function tests (PFTs) should include spirometry, diffusion capacity, and lung volumes. PFTs will be performed at screening and in the event of pulmonary symptoms as clinically indicated
d. Baseline ophthalmic exams will be performed by a board-certified ophthalmologist and will include the following: visual acuity, indirect fundoscopy, slit lamp examination under dilatation, intraocular pressure measurement, and corneal photography. A Schirmer test will be performed at baseline for all patients, and for patients who experience ocular symptoms, it will be repeated at the first on-study ophthalmic examination and at subsequently if clinically indicated. May be performed within 14 days of Cycle 1, Day 1. Ocular symptom assessment will be performed prior to the start of each cycle by the treating physician or other qualified individual. If the subject reports ocular symptoms then IMGN853 will be stopped and the subject will then be referred to an ophthalmologist for a complete examination (detailed in full protocol). Patients who experience ocular toxicity will have a complete ophthalmologic exam performed every other cycle, including patients with blurred vision but normal eye exams. All patients who experience ocular toxicity will be followed until resolution.
e. Off-treatment evaluation. Off-treatment visit will occur within 7 days of the decision to discontinue study treatment. Participants will be followed for 30 days after the last dose of study drug for adverse events. Participants will be followed for 3 years by phone after removal from study or until death, whichever occurs first. Survival status should be checked every 6 months during that time
f. See Appendix D. The patient should continue use of the corticosteroid eye drops per the ophthalmologist recommendation after discontinuation of mirvetuximab soravtansine. The patient should continue the lubricating eye drops for 4 weeks following discontinuation of mirvetuximab soravtansine.

MEASUREMENT OF EFFECT

Measurement of effect will be assessed by both the Response Evaluation Criteria in Solid Tumors (RECIST) Version 1.1 and by the Immune-related Response Criteria Derived from RECIST 1.1 (irRECIST).

11.1 Response Evaluation Criteria in Solid Tumors (RECIST) Version 1.1 Guidelines

11.1.1 Categorizing Lesions at Baseline

Measurable Lesions

- Lesions that can be accurately measured in at least one dimension.
- Lesions with longest diameter twice the slice thickness and at least 10mm or greater when assessed by CT or MRI (slice thickness 5-8mm).
- Lesions with longest diameter at least 20mm when assessed by Chest X-ray.
- Superficial lesions with longest diameter 10mm or greater when assessed by caliper.
- Malignant lymph nodes with the short axis 15mm or greater when assessed by CT.

Note: The shortest axis is used as the diameter for malignant lymph nodes, longest axis for all other measurable lesions.

Note: Tumor lesions that are situated in a previously irradiated area will be considered measurable if there has been progression of disease at that site since radiation occurred.

Normal sites

- Cystic lesions: Simple cysts should not be considered as malignant lesions and should not be recorded either as target or non-target disease. Cystic lesions thought to represent cystic metastases can be measurable lesions, if they meet the specific definition above.
- If non-cystic lesions are also present, these are preferred as target lesions.
- Normal nodes: Nodes with short axis <10mm are considered normal and should not be recorded or followed either as measurable or non-measurable disease.

11.1.2 Recording Tumor Assessments

All sites of disease must be assessed at baseline. Baseline assessments should be done as close as possible prior to study start. For an adequate baseline assessment, all required scans must be done within 28 days prior to treatment and all disease must be documented appropriately. If baseline assessment is inadequate, subsequent statuses generally should be indeterminate.

Target lesions

All measurable lesions up to a maximum of 2 lesions per organ, 5 lesions in total, representative of all involved organs, should be identified as target lesions at baseline. Target lesions should be selected on the basis of size (longest lesions) and suitability for accurate repeated measurements.

Record the longest diameter for each lesion, except in the case of pathological lymph nodes for which the short axis should be recorded. The sum of the diameters (longest for non-nodal lesions, short axis for nodal lesions) for all target lesions at baseline will be the basis for comparison to assessments performed on study. If two target lesions coalesce, the measurement of the coalesced mass is used. If a large target lesion splits, the sum of the parts is used. Measurements for target lesions that become small should continue to be recorded. If a target lesion becomes too small to measure, 0mm should be recorded if the lesion is considered to have disappeared; otherwise a default value of 5mm should be recorded.

Note: When nodal lesions decrease to <10mm (normal), the actual measurement should still be recorded.

Non-target disease

All non-measurable disease is non-target. All measurable lesions not identified as target lesions are also included as non-target disease. Measurements are not required but rather assessments will be expressed as ABSENT, INDETERMINATE, PRESENT/NOT INCREASED, INCREASED. Multiple non-target lesions in one organ may be recorded as a single item on the case report form (eg, 'multiple enlarged pelvic lymph nodes' or 'multiple liver metastases').

11.1.3 Objective Response Status at Each Evaluation

Disease sites must be assessed using the same technique as baseline, including consistent administration of contrast and timing of scanning. If a change needs to be made the case must be discussed with the radiologist to determine if substitution is possible. If not, subsequent objective statuses are indeterminate.

Target disease

- Complete Response (CR): Complete disappearance of all target lesions with the exception of nodal disease. All target nodes must decrease to normal size (short axis <10mm). All target lesions must be assessed.
- Partial Response (PR): Greater than or equal to 30% decrease under baseline of the sum of diameters of all target measurable lesions. The short diameter is used in the sum for target nodes, while the longest diameter is used in the sum for all other target lesions. All target lesions must be assessed.
- Stable: Does not qualify for CR, PR or Progression. All target lesions must be assessed. Stable can follow PR only in the rare case that the sum increases by less than 20% from the nadir, but enough that a previously documented 30% decrease no longer holds.
- Objective Progression (PD): 20% increase in the sum of diameters of target measurable lesions above the smallest sum observed (over baseline if no decrease in the sum is observed during therapy), with a minimum absolute increase of 5mm.
- Indeterminate. Progression has not been documented, and
    - One or more target measurable lesions have not been assessed;

or

- Assessment methods used were inconsistent with those used at baseline;

  or

- One or more target lesions cannot be measured accurately (eg, poorly visible unless due to being too small to measure);

  or

- One or more target lesions were excised or irradiated and have not reappeared or increased.

Non-target disease

- CR: Disappearance of all non-target lesions and normalization of tumor marker levels. All lymph nodes must be 'normal' in size (<10mm short axis).
- Non-CR/Non-PD: Persistence of any non-target lesions and/or tumor marker level above the normal limits.
- PD: Unequivocal progression of pre-existing lesions. Generally the overall tumor burden must increase sufficiently to merit discontinuation of therapy. In the presence of SD or
- PR in target disease, progression due to unequivocal increase in non-target disease should be rare.
- Indeterminate: Progression has not been determined and one or more non-target sites were not assessed or assessment methods were inconsistent with those used at baseline.

New Lesions

The appearance of any new unequivocal malignant lesion indicates PD. If a new lesion is equivocal, for example due to its small size, continued assessment will clarify the etiology. If repeat assessments confirm the lesion, then progression should be recorded on the date of the initial assessment. A lesion identified in an area not previously scanned will be considered a new lesion. Supplemental Investigations If CR determination depends on a residual lesion that decreased in size but did not disappear completely, it is recommended the residual lesion be investigated with biopsy or fine needle aspirate. If no disease is identified, objective status is CR. If progression determination depends on a lesion with an increase possibly due to necrosis, the lesion may be investigated with biopsy or fine needle aspirate to clarify status.

Objective/Subjective

Patients requiring discontinuation of treatment without objective evidence of disease progression should not be reported as PD on tumor assessment CRFs. This should be indicated on the end of treatment CRF as off treatment due to Global Deterioration of Health Status. Every effort should be made to document objective progression even after discontinuation of treatment.

For Participants with Measurable Disease (*i.e.*, Target Disease)

Table 10: Best Overall Response for Measurable Disease (Target Disease)

Target	Non-Target	New	Overall	Best Overall Response when

Lesions	Lesions	Lesions	Response	Confirmation is Required*
CR	CR	No	CR	≥4 wks Confirmation**
CR	Non-CR/Non-PD	No	PR	≥4 wks Confirmation**
CR	Not evaluated	No	PR	
PR	Non-CR/Non-PD/not evaluated	No	PR	
SD	Non-CR/Non-PD/not evaluated	No	SD	Documented at least once ≥4 wks from baseline**
PD	Any	Yes or No	PD	no prior SD, PR or CR
Any	PD***	Yes or No	PD	
Any	Any	Yes	PD	

* See RECIST 1.1 manuscript for further details on what is evidence of a new lesion.
** Only for non-randomized trials with response as primary endpoint.
*** In exceptional circumstances, unequivocal progression in non-target lesions may be accepted as disease progression.

Note: Participants with a global deterioration of health status requiring discontinuation of treatment without objective evidence of disease progression at that time should be reported as "*symptomatic deterioration.*" Every effort should be made to document the objective progression even after discontinuation of treatment.

Determination of Best Overall Response

The best overall response is the best response recorded from the start of the treatment until disease progression/recurrence (taking as reference for progressive disease the smallest sum on study). For CR and PR, the patient's best response assignment will depend on the achievement of both measurement and confirmation criteria. CR and PR must be confirmed by 2 measurements at least 4 weeks apart. In the case of SD, follow up measurements must have met the SD criteria at least once after study entry at a minimum interval of 6 weeks.

11.2 Immune-related Response Criteria Derived from RECIST 1.1 (irRECIST)

Increasing clinical experience indicates that traditional response criteria may not be sufficient to fully characterize activity in this new era of targeted therapies and/or biologics.

This is particularly true for immunotherapeutic agents such as anti-CTLA-4 and anti-PD-1 / anti PD-L1 which exert the antitumor activity by augmenting activation and proliferation of T-cells, thus leading to tumor infiltration by T-cells and tumor regression rather than direct cytotoxic effects [31, 32]. Clinical observations of patients with advanced melanoma treated with ipilimumab, for example, suggested that conventional response assessment criteria such as Response Evaluation Criteria in Solid Tumors (RECIST) and WHO criteria are not sufficient to fully characterize patterns of tumor response to immunotherapy because tumors treated with

immunotherapeutic agentsmay show additional response patterns that are not described in these conventional criteria [33, 34].

Furthermore, the conventional tumor assessment criteria (RECIST and WHO criteria) have been reported as not capturing the existence of a subset of patients who have an OS similar to those who have experienced CR or PR but were flagged as PD by WHO criteria [34, 35].

On these grounds, a tumor assessment system has been developed that incorporates these delayed or flare-type responses into the RECIST 1.1 criteria (irRECIST) [33].

For irRECIST, only target and measurable lesions are taken into account. In contrast to the RECIST 1.1, the irRECIST criteria:

- require confirmation of both progression and response by imaging at least 4 weeks from the date first documented, and
- do not necessarily score the appearance of new lesions as progressive disease if the sum of lesion diameters of target lesions (minimum of 10 mm per lesion, maximum of 5 target lesions, maximum of 2 per organ) and measurable new lesions does not increase by >= 20%.

The same method of assessment and the same technique should be used to characterize each identified and reported target lesion(s) at baseline and throughout the trial.

irRECIST criteria are defined as follows:

- Overall immune-related complete response (irCR): Complete disappearance of all lesions (whether measurable or not) and no new lesions. All measurable lymph nodes also must have a reduction in short axis to <10 mm.
- Overall immune-related partial response (irPR): Sum of the diameters (longest for nonnodal lesions, shortest for nodal lesions) of target and new measurable lesions decreases >= 30%.
- Overall immune-related stable disease (irSD): Sum of the diameters (longest for nonnodal lesions, shortest for nodal lesions) of target and new measurable lesions neither irCR, irPR, (compared to baseline) or immune-related progressive disease (irPD, compared to nadir).
- Overall immune-related progressive disease (irPD): Sum of the diameters (longest for non-nodal lesions, shortest for nodal lesions) of target and new measurable lesions increases >= 20% (compared to nadir), confirmed by a repeat, consecutive observation at least 4weeks from the date first documented.

New measurable lesions: Incorporated into tumor burden (ie, added to the target lesion measurements). A lymph node has to be >= 15 mm in short axis to be a measurable new lesion and its short axis measurement is included in the sum. Up to 2 new lesions per organ and up to 5 new lesions in total can be added to the measurements.

New non-measurable lesions: Do not define progression but preclude irCR.

Overall responses derived from changes in index, non-index, and new lesions are outlines in the table.

Table 11: Overall Response for irRECIST Derived from Changes in Index, Non-index and New Lesions

**Measurable Response**	**Non-measurable response**		**Overall Response using irRECIST**[b]
**Index and New Measurable Lesions (Tumor Burden)**[a]	**Non-Index Lesions**	**New non-measurable lesions**	
Decrease 100%	Absent	Absent	irCR
Decrease 100%	Stable	Any	irPR
Decrease 100%	Unequivocal progression	Any	irPR
Decrease ≥ 30%	Absent / stable	Any	irPR
Decrease ≥ 30%	Unequivocal progression	Any	irPR
Decrease < 30% and increase <20%	Absent/ stable	Any	irSD
Decrease < 30% and increase <20%	Unequivocal progression	Any	irSD
Increase ≥ 20%	Any	Any	irPD

a. Decrease assessed relative to baseline. Increase assessed relative to nadir.
b. Response (irCR and irPR) and progression (irPD) must be confirmed by a second, consecutive assessment at least 4 weeks apart.

11.3 Methods for Evaluation of Disease

All measurements should be taken and recorded in metric notation using a ruler, calipers, or a digital measurement tool. All baseline evaluations should be performed as closely as possible to the beginning of treatment and never more than 4 weeks before the beginning of the treatment.

The same method of assessment and the same technique should be used to characterize each identified and reported lesion at baseline and during follow-up. Imaging-based evaluation is preferred to evaluation by clinical examination unless the lesion(s) being followed cannot be imaged but are assessable by clinical exam.

Clinical lesions. Clinical lesions will only be considered measurable when they are superficial (*e.g.*, skin nodules and palpable lymph nodes) and ≥10 mm in diameter as assessed using calipers (*e.g.*, skin nodules). In the case of skin lesions, documentation by color photography, including a ruler to estimate the size of the lesion, is recommended.

Chest x-ray. Lesions on chest x-ray are acceptable as measurable lesions when they are clearly defined and surrounded by aerated lung; however, CT is preferable.

Conventional CT and MRI. This guideline has defined measurability of lesions on CT scan based on the assumption that CT thickness is 5mm or less. If CT scans have slice thickness greater than

5 mm, the minimum size of a measurable lesion should be twice the slice thickness. MRI is also acceptable in certain situations (*e.g.* for body scans).

Use of MRI remains a complex issue. MRI has excellent contrast, spatial, and temporal resolution; however, there are many image acquisition variables involved in MRI, which greatly impact image quality, lesion conspicuity, and measurement. Furthermore, the availability of MRI is variable globally. As with CT, if an MRI is performed, the technical specifications of the scanning sequences used should be optimized for the evaluation of the type and site of disease. Furthermore, as with CT, the modality used at follow-up should be the same as was used at baseline and the lesions should be measured/assessed on the same pulse sequence. It is beyond the scope of the RECIST guidelines to prescribe specific MRI pulse sequence parameters for all scanners, body parts, and diseases. Ideally, the same type of scanner should be used and the image acquisition protocol should be followed as closely as possible to prior scans. Body scans should be performed with breath-hold scanning techniques, if possible.

<u>FDG-PET.</u> While FDG-PET response assessments need additional study, it is sometimes reasonable to incorporate the use of FDG-PET scanning to complement CT scanning in assessment of progression (particularly possible “new” disease). New lesions on the basis of FDG-PET imaging can be identified according to the following algorithm:

(a) Negative FDG-PET at baseline, with a positive FDG-PET at follow-up is a sign of PD based on a new lesion.
(b) No FDG-PET at baseline and a positive FDG-PET at follow-up: If the positive FDG-PET at follow-up corresponds to a new site of disease confirmed by CT, this is PD. If the positive FDG-PET at follow-up is not confirmed as a new site of disease on CT, additional follow-up CT scans are needed to determine if there is truly progression occurring at that site (if so, the date of PD will be the date of the initial abnormal FDG-PET scan). If the positive FDG-PET at follow-up corresponds to a pre-existing site of disease on CT that is not progressing on the basis of the anatomic images, this is not PD.
(c) FDG-PET may be used to upgrade a response to a CR in a manner similar to a biopsy in cases where a residual radiographic abnormality is thought to represent fibrosis or scarring. The use of FDG-PET in this circumstance should be prospectively described in the protocol and supported by disease-specific medical literature for the indication. However, it must be acknowledged that both approaches may lead to false positive CR due to limitations of FDG-PET and biopsy resolution/sensitivity.

Note: A “positive” FDG-PET scan lesion means one which is FDG avid with an uptake greater than twice that of the surrounding tissue on the attenuation corrected image.

<u>PET-CT.</u> At present, the low dose or attenuation correction CT portion of a combined PET-CT is not always of optimal diagnostic CT quality for use with RECIST measurements. However, if the site can document that the CT performed as part of a PET-CT is of identical diagnostic quality to a diagnostic CT (with IV and oral contrast), then the CT portion of the PET-CT can be used for RECIST measurements and can be used interchangeably with conventional CT in accurately measuring cancer lesions over time. Note, however, that the PET portion of the CT introduces additional data which may bias an investigator if it is not routinely or serially performed.

Tumor markers. Tumor markers alone cannot be used to assess response. If markers are initially above the upper normal limit, they must normalize for a participant to be considered in complete clinical response.

DATA REPORTING / REGULATORY REQUIREMENTS

Adverse event lists, guidelines, and instructions for AE reporting can be found in Section 7 (Adverse Events: List and Reporting Requirements).

12.1 Data Reporting

12.1.1 Method

The Office of Data Quality (ODQ) will collect, manage, and perform quality checks on the data for this study.

12.1.2 Responsibility for Data Submission

Investigative sites within DF/HCC or DF/PCC are responsible for submitting data and/or data forms to the Office of Data Quality (ODQ) in accordance with DF/HCC policies.

12.2 Data Safety Monitoring

The DF/HCC Data and Safety Monitoring Committee (DSMC) will review and monitor toxicity and accrual data from this study. The committee is composed of medical oncologists, research nurses, pharmacists and biostatisticians with direct experience in cancer clinical research. Information that raises any questions about participant safety will be addressed with the Overall PI and study team.

The DSMC will review each protocol up to four times a year with the frequency determined by the outcome of previous reviews. Information to be provided to the committee may include: up-to-date participant accrual; current dose level information; DLT information; all grade 2 or higher unexpected adverse events that have been reported; summary of all deaths occurring within 30 days of intervention for Phase I or II protocols; for gene therapy protocols, summary of all deaths while being treated and during active follow-up; any response information; audit results, and a summary provided by the study team. Other information (e.g. scans, laboratory values) will be provided upon request.

12.2 Multi-Center Guidelines

This protocol will adhere to DF/HCC Policy MULTI-100 and the requirements of the DF/HCC Multi-Center Data and Safety Monitoring Plan. The specific responsibilities of the Sponsor-Investigator, Coordinating Center, and Participating Institutions and the procedures for auditing are presented in Appendix B.

STATISTICAL CONSIDERATIONS

This is an open label, two-stage, non-randomized phase 2 trial examining mirvetuximab soravtansine (IMGN853) and pembrolizumab in participants with advanced or recurrent serous endometrial cancer (EC). The combination will be tested in patients with microsatellite stable (MSS) and FRα positive tumors.

Enrollment will follow a standard Simon two-stage optimal design for a single cohort to inform whether the combination has significant clinical activity worthy of further evaluation. Statistical considerations are developed for a co-primary objectives to evaluate the objective response rate (ORR) and rate of progression-free survival at 6 months (PFS6), with a two-stage design that allows for early stopping for futility.

Target enrollment is a maximum of 35 patients. Up to 19 patients of the total cohort may have received prior therapy targeting the PD-1/PD-L1 pathway.

13.1 Study Design/Endpoints

Primary Endpoints

To assess the activity of mirvetuximab soravtasine in combination with pembrolizumab in patients with recurrent or persistent MSS and FRα positive endometrial cancer by evaluating the frequency of patients who survive progression-free for at least 6 months (PFS6) after initiating therapy or have objective tumor response.

Statistical considerations are developed for co-primary objectives to evaluate the objective response rate (ORR) by RECIST 1.1 and rate of progression-free survival at 6 months (PFS6), with a two-stage design that allows for early stopping for futility.

A two-stage test was constructed using the method of Sill, Rubinstein, Litwin and Yothers [36] with the goal of stopping early for futility to limit patient exposure to an inactive agent while restricting the probabilities of type I and type II errors to approximately 10% and 15%, respectively. For the co-primary endpoints, a true ORR of 5% or less and a rate of progression-free survival at 6 months of (PFS6) 10% or less would not be of clinical interest (H0: $\pi_{OR} \leq 5\%$ AND $\pi_{PFS6} \leq 10\%$), whereas an improvement to a 20% objective response rate or 30% PFS6 rate would warrant further investigation of the study treatment. In the first stage, 16 patients will be enrolled and accrual will pause. If there are at least two objective responses or two patients progression-free at 6 months among the first 16 patients, the second stage of accrual will commence where an additional 19 patients will be enrolled. If at the end of the trial there are at least 4 treated patients with an objective response or 8 patients progression-free at 6 months, the study treatment will be considered worthy of further study. Specifically, analysis of historical data (GOG129 and GOG229 series) based on similar population of patients with endometrial cancer where the levels of activity were believed to be inactive to modestly active shows that an unacceptable rate for objective response (π_{OR}) is 5% and for PFS6 (π_{PFS6}) is 10%, respectively. A bivariate test of the co-primary endpoints is constructed as follows:

$$H0: \pi_{OR} \leq 5\% \text{ AND } \pi_{PFS6} \leq 10\%$$

vs.

$$H1: \pi_{OR} > 5\% \text{ OR } \pi_{PFS6} > 10\%$$

Targeted alternative hypothesis: An absolute improvement in the rate of OR by 15% or PFS6 by 20% would be of clinical interest. Power is determined under two specific hypotheses (Hr: π_{OR} = 20%, π_{PFS6} = 10%) and (Hs: π_{OR} = 5%, π_{PFS6} = 30%) where sufficient activity is seen in only each endpoint, respectively. Sample size is selected to have sufficient power to reject the null under cases that assume either (a) independence or (b) dependence among the co-primary endpoints. The decision rules and operating characteristics of the two-stage design are summarized in the Table below:

Table 12: Decision rules and operating characteristics of the two-stage design

	**Endpoint**	**Objective Response**	**PFS6**
	Null hypothesis	5%	10%
	Alternative Hypothesis	20%	30%
**Stage 1**	Number of Patients	16	
	Minimum number of events to continue to Stage 2	2	2
	PET (Independence)	0.417	
	PET (Dependence)	0.502	
**Stage 2**	Number of Patients	35	
	Minimum number of events to reject the null hypothesis	4	8
	Alpha (Independence)	0.098	
	Alpha (Dependence)	0.88	
	Power (Independence)	0.889	0.873
	Power (Dependence)	0.846	0.862

13.2 **Sample Size, Accrual Rate and Study Duration**

Table 13: Accrual Target Demographics

**Accrual Targets**					
**Ethnic Category**	**Sex/Gender**				
	**Females**		**Males**		**Total**
Hispanic or Latino	5	+	0	=	5
Not Hispanic or Latino	30	+	0	=	35
**Ethnic Category: Total of all subjects**	**35**	+	**0**	=	**35**
**Racial Category**					
American Indian or Alaskan Native	0	+	0	=	0
Asian	5	+	0	=	5

Black or African American	3	+	0	=	3
Native Hawaiian or other Pacific Islander	0	+	0	=	0
White	27	+	0	=	27
**Racial Category: Total of all subjects**	35	+	0	=	35
	**35**		**0**		**35**

13.3 **Stratification Factors**

N/A

13.4 **Interim Monitoring Plan**

See Section 13.1

13.5 **Analysis of Primary Endpoints**

See Section 13.1

13.6 **Analysis of Secondary Endpoints**

The distributions of progression-free- and overall survival times will be estimated by using Kaplan-Meier analysis. Immune-related objective response rate will be estimated as described in Section 11.2. Immune-related progression-free survival (irPFS) rate is defined as time from treatment initiation to death or to immune-related progression of disease (irPD) as defined in Section 11.2. Duration of response (DoR) is defined as time from best overall response to the time of disease progression, where best overall response and disease progression are as defined in Section 11.1.

Exploratory endpoints are not specifically planned but could include biomarker expression and immune population analysis, and descriptive statistics including the mean, standard deviation, median, minimum, and maximum values, for the cohort. For baseline categorical data, the number and percentage of patients in each category will be collected. Appropriate statistical methods may be used to investigate any possible relationship of biomarker levels with response to baseline characteristics and response to study treatment.

An additional exploratory endpoint includes a comparison of the assessment of activity of mirvetuximab soravtansine in combination with pembrolizumab as described in Section 13.1 between patients with and without prior exposure to IO therapy targeting the PD-1/PD-L1 pathway. If at least 6 of the 19 patients meet the endpoint of either OR and/or PFS6 (i.e. exhibit clinical benefit), this would generate a 95% binomial coefficient of 12.6% - 56.5%, which would exclude the possibility that clinical benefit with this regimen is <10% in the IO-exposed patients.

13.7 **Reporting and Exclusions**

13.7.1 Evaluation of Toxicity

All participants will be evaluable for toxicity from the time of their first treatment.

13.7.2 Evaluation of the Primary Efficacy Endpoint

All participants included in the study must be assessed for response to treatment, even if there are major protocol treatment deviations or if they are ineligible. Each participant should be assigned one of the following categories: 1) complete response, 2) partial response, 3) stable disease, 4) progressive disease, 5) early death from malignant disease, 6) early death from toxicity, 7) early death because of other cause, or 9) unknown (not assessable, insufficient data). By arbitrary convention, category 9 usually designates the “unknown” status of any type of data in a clinical database.

All participants who have measurable disease present at baseline, have received at least one cycle of therapy, and have had their disease re-evaluated will be considered evaluable for response. Participants in response categories 5-9 will not be considered evaluable but will remain within the intention to treat analysis.

Patients who develop brain metastasis on study, but continue on study after definitive treatment of their cranial disease, will be considered to have had progressive disease at the point of the diagnosis of the initial brain metastasis.

All conclusions should be based on all evaluable participants. Subanalyses may then be performed on the basis of a subset of participants, excluding those for whom major protocol deviations have been identified (e.g., early death due to other reasons, early discontinuation of treatment, major protocol violations, etc.). However, these subanalyses may not serve as the basis for drawing conclusions concerning treatment efficacy, and the reasons for excluding participants from the analysis should be clearly reported. The 95% confidence intervals should also be provided.

PUBLICATION PLAN

Publication guidelines exist within the DF/HCC Gynecologic Oncology Program. The study principal investigator will be responsible for collection of data, interpretation of data, monitoring of toxicities, and publication of abstracts and final manuscripts. The principal investigator chooses the different authorship slots per the DF/HCC gynecologic oncology program guidelines.

The information obtained during the conduct of this clinical study is confidential, and disclosure to third parties other than those noted below is prohibited. All information concerning the product as well as any matter concerning the operation of the Sponsor, such as clinical indications for the drug, its formula, methods of manufacture and other scientific data relating to it, that have been provided by the Sponsor and are unpublished, are confidential and must remain the sole property of the Sponsor. The Investigator will agree to use the information only for the

purposes of carrying out this study and for no other purpose unless prior written permission from the Sponsor is obtained.

Information obtained during the conduct of this study will be used by ImmunoGen in connection with the development of the study drug. The study Investigator is obliged to provide ImmunoGen with complete test results and all data developed in this study. This information may be disclosed to other physicians who are conducting similar studies and to the FDA, as deemed necessary by the Sponsor. Patient-specific information may be provided to other appropriate medical personnel related to the care of that patient only with patient's prior consent.

The Investigator and any other clinical personnel associated with this study will not publish the results of the study, in whole or in part, at any time, unless they have consulted with ImmunoGen, provided ImmunoGen a copy of the draft document intended for publication, and obtained ImmunoGen's written consent for such publication. All information obtained during the conduct of this study will be regarded as confidential. ImmunoGen will use the information for registration purposes and for the general development of the drug.

The results should be made public within 24 months of reaching the end of the study. The end of the study is the time point at which the last data items are to be reported, or after the outcome data are sufficiently mature for analysis, as defined in the section on Sample Size, Accrual Rate and Study Duration. If a report is planned to be published in a peer-reviewed journal, then that initial release may be an abstract that meets the requirements of the International Committee of Medical Journal Editors. A full report of the outcomes should be made public no later than three (3) years after the end of the study.

REFERENCES

1. Simon, R., *Optimal two-stage designs for phase II clinical trials.* Control Clin Trials, 1989. **10**(1): p. 1-10.
2. Siegel, R.L., et al., *Colorectal cancer statistics, 2017.* CA: a cancer journal for clinicians, 2017. **67**(3): p. 177-193.
3. Jemal, A., et al., *Cancer statistics, 2009.* CA: a cancer journal for clinicians, 2009. **59**(4): p. 225-249.
4. Thigpen, J.T., et al., *Phase III trial of doxorubicin with or without cisplatin in advanced endometrial carcinoma: a gynecologic oncology group study.* J Clin Oncol, 2004. **22**(19): p. 3902-8.
5. Ball, H.G., et al., *A phase II trial of paclitaxel in patients with advanced or recurrent adenocarcinoma of the endometrium: a Gynecologic Oncology Group study.* Gynecol Oncol, 1996. **62**(2): p. 278-81.
6. Fleming, G.F., et al., *Phase III randomized trial of doxorubicin + cisplatin versus doxorubicin + 24-h paclitaxel + filgrastim in endometrial carcinoma: a Gynecologic Oncology Group study.* Ann Oncol, 2004. **15**(8): p. 1173-8.
7. Fleming, G.F., et al., *Phase III trial of doxorubicin plus cisplatin with or without paclitaxel plus filgrastim in advanced endometrial carcinoma: a Gynecologic Oncology Group Study.* J Clin Oncol, 2004. **22**(11): p. 2159-66.
8. Bradford, L.S., et al., *Advances in the management of recurrent endometrial cancer.* Am J Clin Oncol, 2015. **38**(2): p. 206-12.
9. Fleming, G.F., *Second-Line Therapy for Endometrial Cancer: The Need for Better Options.* J Clin Oncol, 2015. **33**(31): p. 3535-40.
10. Aghajanian, C., et al., *Phase II trial of bevacizumab in recurrent or persistent endometrial cancer: a Gynecologic Oncology Group study.* J Clin Oncol, 2011. **29**(16): p. 2259-65.
11. Moore, K.N., et al., *Safety and Activity of Mirvetuximab Soravtansine (IMGN853), a Folate Receptor Alpha-Targeting Antibody-Drug Conjugate, in Platinum-Resistant Ovarian, Fallopian Tube, or Primary Peritoneal Cancer: A Phase I Expansion Study.* J Clin Oncol, 2017. **35**(10): p. 1112-1118.
12. Cancer Genome Atlas Research, N., et al., *Integrated genomic characterization of endometrial carcinoma.* Nature, 2013. **497**(7447): p. 67-73.
13. Howitt, B.E., et al., *Association of Polymerase e-Mutated and Microsatellite-Instable Endometrial Cancers With Neoantigen Load, Number of Tumor-Infiltrating Lymphocytes, and Expression of PD-1 and PD-L1.* JAMA Oncol, 2015. **1**(9): p. 1319-23.
14. Le, D.T., et al., *PD-1 Blockade in Tumors with Mismatch-Repair Deficiency.* N Engl J Med, 2015. **372**(26): p. 2509-20.
15. Diaz, L.A., et al., *Programmed death-1 blockade in mismatch repair deficient cancer independent of tumor histology*, 2016, American Society of Clinical Oncology.
16. Ott, P.A., et al., *Safety and Antitumor Activity of Pembrolizumab in Advanced Programmed Death Ligand 1-Positive Endometrial Cancer: Results From the KEYNOTE-028 Study.* J Clin Oncol, 2017. **35**(22): p. 2535-2541.
17. Senol, S., et al., *Folate receptor alpha expression and significance in endometrioid endometrium carcinoma and endometrial hyperplasia.* Int J Clin Exp Pathol, 2015. **8**(5):

p. 5633-41.
18. Brown Jones, M., et al., *Rationale for folate receptor alpha targeted therapy in "high risk" endometrial carcinomas.* Int J Cancer, 2008. **123**(7): p. 1699-703.
19. Martin, L., et al. *Phase I expansion study of mirvetuximab soravtansine, a folate receptor alpha (FRalpha)-targeting antibody-drug conjugate (ADC), in patients with endometrial cancer*. in *European Society for Gynecologic Oncology*. 2017.
20. Muller, P., et al., *Trastuzumab emtansine (T-DM1) renders HER2+ breast cancer highly susceptible to CTLA-4/PD-1 blockade.* Sci Transl Med, 2015. **7**(315): p. 315ra188.
21. Muller, P., et al., *Microtubule-depolymerizing agents used in antibody-drug conjugates induce antitumor immunity by stimulation of dendritic cells.* Cancer Immunol Res, 2014. **2**(8): p. 741-55.
22. Skaletskaya, A., et al., *Treatment of Tumor Cells with Mirvetuximab Soravtansine, a FR-alpha-Targeting Antibody-Drug Conjugate (ADC), Activates Monocytes Through Fc-Fc gamma R Interaction and Immunogenic Cell Death*, in *Society for the Immunotherapy of Cancer*2016.
23. O'Malley, D.M., et al. *Safety findings from FORWARD II: A phase 1b study evaluating the folate receptor alpha (FRα)-targeting antibody-drug conjugate (ADC) mirvetuximab soravtansine (IMGN853) in combination with bevacizumab, carboplatin, pegylated liposomal doxorubicin (PLD), or pembrolizumab in patients (pts) with ovarian cancer.* in *ASCO*. 2017.
24. Ascierto, P.A., *Ipilimumab in the treatment of metastatic melanoma: a summary of recent studies.* Tumori, 2013. **99**(6): p. 302e-5e.
25. Herbst, R.S., et al., *Predictive correlates of response to the anti-PD-L1 antibody MPDL3280A in cancer patients.* Nature, 2014. **515**(7528): p. 563-7.
26. Garon, E.B., et al., *Pembrolizumab for the treatment of non-small-cell lung cancer.* N Engl J Med, 2015. **372**(21): p. 2018-28.
27. Borghaei, H., et al., *Nivolumab versus Docetaxel in Advanced Nonsquamous Non-Small-Cell Lung Cancer.* N Engl J Med, 2015. **373**(17): p. 1627-39.
28. Brahmer, J., et al., *Nivolumab versus Docetaxel in Advanced Squamous-Cell Non-Small-Cell Lung Cancer.* N Engl J Med, 2015. **373**(2): p. 123-35.
29. Rizvi, N.A., et al., *Cancer immunology. Mutational landscape determines sensitivity to PD-1 blockade in non-small cell lung cancer.* Science, 2015. **348**(6230): p. 124-8.
30. Snyder, A., et al., *Genetic basis for clinical response to CTLA-4 blockade in melanoma.* N Engl J Med, 2014. **371**(23): p. 2189-2199.
31. Hodi, F.S., et al., *Immunologic and clinical effects of antibody blockade of cytotoxic T lymphocyte-associated antigen 4 in previously vaccinated cancer patients.* Proc Natl Acad Sci U S A, 2008. **105**(8): p. 3005-10.
32. Hoos, A., et al., *Improved endpoints for cancer immunotherapy trials.* J Natl Cancer Inst, 2010. **102**(18): p. 1388-97.
33. Nishino, M., et al., *Optimizing immune-related tumor response assessment: does reducing the number of lesions impact response assessment in melanoma patients treated with ipilimumab?* J Immunother Cancer, 2014. **2**: p. 17.
34. Wolchok, J.D., et al., *Guidelines for the evaluation of immune therapy activity in solid tumors: immune-related response criteria.* Clin Cancer Res, 2009. **15**(23): p. 7412-20.
35. Nishino, M., et al., *Personalized tumor response assessment in the era of molecular medicine: cancer-specific and therapy-specific response criteria to complement pitfalls of*

RECIST. AJR Am J Roentgenol, 2012. **198**(4): p. 737-45.

36. Sill, M.W., et al., *A method for utilizing co-primary efficacy outcome measures to screen regimens for activity in two-stage Phase II clinical trials.* Clin Trials, 2012. **9**(4): p. 385-95.

APPENDIX A ECOG PERFORMANCE STATUS

**ECOG Performance Status Scale**		**Karnofsky Performance Scale**	
Grade	Descriptions	Percent	Description
0	Normal activity. Fully active, able to carry on all pre-disease performance without restriction.	100	Normal, no complaints, no evidence of disease.
		90	Able to carry on normal activity; minor signs or symptoms of disease.
1	Symptoms, but ambulatory. Restricted in physically strenuous activity, but ambulatory and able to carry out work of a light or sedentary nature (*e.g.*, light housework, office work).	80	Normal activity with effort; some signs or symptoms of disease.
		70	Cares for self, unable to carry on normal activity or to do active work.
2	In bed <50% of the time. Ambulatory and capable of all self-care, but unable to carry out any work activities. Up and about more than 50% of waking hours.	60	Requires occasional assistance, but is able to care for most of his/her needs.
		50	Requires considerable assistance and frequent medical care.
3	In bed >50% of the time. Capable of only limited self-care, confined to bed or chair more than 50% of waking hours.	40	Disabled, requires special care and assistance.
		30	Severely disabled, hospitalization indicated. Death not imminent.
4	100% bedridden. Completely disabled. Cannot carry on any self-care. Totally confined to bed or chair.	20	Very sick, hospitalization indicated. Death not imminent.
		10	Moribund, fatal processes progressing rapidly.
5	Dead.	0	Dead.

DF/HCC Protocol #: 18-602

APPENDIX B: DANA-FARBER/HARVARD CANCER CENTER MULTI-CENTER DATA AND SAFETY MONITORING PLAN

TABLE OF CONTENTS

1. INTRODUCTION 4
1.1. Purpose..4

2. GENERAL ROLES AND RESPONSIBILITIES 4
2.1. Coordinating Center..4
2.2. External Site..5

3. DF/HCC REQUIREMENTS FOR MULTI-CENTER PROTOCOLS 6
3.1. Protocol Revisions and Closures 6
3.2. Informed Consent Requirements 6
3.3. IRB Re-Approval..6
3.4. DF/HCC Multi-Center Protocol Confidentiality 7
3.5. Participant Registration and Randomization 7
3.6. Initiation of Therapy 8
3.7. Eligibility Exceptions..8
3.8. Data Management..8
3.9. Protocol Reporting Requirements..8

4. MONITORING: QUALITY CONTROL 9
4.1. Ongoing Monitoring of Protocol Compliance 9
4.2. Monitoring Reports..10
4.3. Accrual Monitoring..10

5. AUDITING: QUALITY ASSURANCE 11
5.1. DF/HCC Internal Audits..11
5.2. Audit Notifications..11
5.3. Audit Reports..11
5.4. External Site Performance 11

1. INTRODUCTION

The Dana-Farber/Harvard Cancer Center Multi-Center Data and Safety Monitoring Plan (DF/HCC DSMP) outlines the procedures for conducting a DF/HCC Multi-Center research protocol. The DF/HCC DSMP serves as a reference for any sites external to DF/HCC that are participating in a DF/HCC clinical trial.

1.1. Purpose

To establish standards that will ensure that a Dana-Farber/Harvard Cancer Center Multi-Center protocol will comply with Federal Regulations, Health Insurance Portability and Accountability Act (HIPAA) requirements and applicable DF/HCC Policies and Operations..

2. GENERAL ROLES AND RESPONSIBILITIES

For DF/HCC Multi-Center Protocols, the following general responsibilities apply, in addition to those outlined in DF/HCC Policies for Sponsor-Investigators:

2.1. Coordinating Center

The general responsibilities of the Coordinating Center may include but are not limited to:

- Assist in protocol development.
- Maintain FDA correspondence, as applicable.
- Review registration materials for eligibility and register participants from Participating Institutions in the DF/HCC clinical trial management system (CTMS).
- Distribute protocol and informed consent document updates to External Sites as needed.
- Oversee the data collection process from External Sites.
- Maintain documentation of Serious Adverse Event (SAE) reports and deviations/violation submitted by External Sites and provide to the DF/HCC Sponsor for timely review and submission to the IRB of record, as necessary.
- Distribute serious adverse events reported to the DF/HCC Sponsor that fall under the reporting requirements for the IRB of record to all External Sites.
- Provide External Sites with information regarding DF/HCC requirements that they will be expected to comply with.
- Carry out plan to monitor External Sites either by on-site or remote monitoring.
- Maintain Regulatory documents of all External Sites which includes but is not limited to the following: local IRB approvals/notifications from all External Sites, confirmation of Federalwide Assurances (FWAs) for all sites, all SAE submissions, Screening Logs for all sites, IRB approved consents for all sites
- Conduct regular communications with all External Sites (conference calls, emails, etc) and maintain documentation all relevant communications.

2.2. External Site

An External Site is an institution that is outside the DF/HCC and DF/PCC consortium that is collaborating with DF/HCC on a protocol where the sponsor is a DF/HCC investigator. The External Site acknowledges the DF/HCC Sponsor as having the ultimate authority and responsibility for the overall conduct of the study.

Each External Site is expected to comply with all applicable DF/HCC requirements stated within this Data and Safety Monitoring Plan and/or the protocol document..

The general responsibilities for each External Site may include but are not limited to:

- Document the delegation of research specific activities to study personnel.
- Commit to the accrual of participants to the protocol.
- Submit protocol and/or amendments to their IRB of record. For studies under a single IRB, the Coordinating Center will facilitate any study-wide submissions..
- Maintain regulatory files as per ICH GCP and federal requirements.
- Provide the Coordinating Center with regulatory documents or source documents as requested.
- Participate in protocol training prior to enrolling participants and throughout the trial as required.
- Update Coordinating Center with research staff changes on a timely basis.
- Register participants through the Coordinating Center prior to beginning research related activities when required by the sponsor.
- Submit Serious Adverse Event (SAE) reports to sponsor, Coordinating Center, and IRB of record as applicable, in accordance with DF/HCC requirements.
- Submit protocol deviations and violations to the Sponsor, Coordinating Center, and IRB of record as applicable..
- Order, store and dispense investigational agents and/or other protocol mandated drugs per federal guidelines and protocol requirements.
- Participate in any quality assurance activities and meet with monitors or auditors at the conclusion of a visit to review findings.
- Promptly provide follow-up and/or corrective action plans for any monitoring queries or audit findings.
- Notify the sponsor immediately of any regulatory authority inspection of this protocol at the External Site.

3. DF/HCC REQUIREMENTS FOR MULTI-CENTER PROTOCOLS

Certain DF/HCC Policy requirements apply to External Sites participating in DF/HCC research. The following section will clarify DF/HCC requirements and further detail the expectations for participating in a DF/HCC Multi-Center protocol.

3.1. Protocol Revisions and Closures

The External Sites will receive notification of protocol revisions and closures from the Coordinating Center. When under a separate IRB, it is the individual External Site's responsibility to notify its IRB of these revisions.

- **Protocol revisions:** External Sites will receive written notification of protocol revisions from the Coordinating Center. All protocol revisions should be IRB approved and implemented within a timely manner from receipt of the notification.

- **Protocol closures and temporary holds:** External Sites will receive notification of protocol closures and temporary holds from the Coordinating Center. Closures and holds will be effective immediately. In addition, the Coordinating Center, will update the External Sites on an ongoing basis about protocol accrual data so that they will be aware of imminent protocol closures.

3.2. Informed Consent Requirements

The DF/HCC approved informed consent document will serve as a template for the informed consent for External Sites. The External Site consent form must follow the consent template as closely as possible and should adhere to specifications outlined in the DF/HCC Guidance Document on Model Consent Language for Investigator-Sponsored Multi-Center Trials. This document will be provided separately to each External Site upon request.

External Sites must send their version of the informed consent document to the Coordinating Center for sponsor review and approval. If the HIPAA authorization is a separate document, please submit to the sponsor for the study record. Once sponsor approval is obtained, the External site may submit to their IRB of record, as applicable. In these cases, the approved consent form must also be submitted to the Coordinating Center after approval by the local IRB for all consent versions.

The Principal Investigator (PI) at each External Site will identify the appropriate members of the study team who will be obtaining consent and signing the consent form for protocols. External Sites must follow the DF/HCC requirement that for all interventional drug, biologic, or device research, only attending physicians may obtain initial informed consent and any re-consent that requires a full revised consent form.

3.3. IRB Re-Approval

Verification of IRB re-approval for the External Sites is required in order to continue research activities. There is no grace period for continuing approvals.

The Coordinating Center will not register participants if a re-approval letter is not received for the External Site on or before the anniversary of the previous approval date.

3.4. DF/HCC Multi-Center Protocol Confidentiality

All documents, investigative reports, or information relating to the participant are strictly confidential. Whenever reasonably feasible, any participant specific reports (i.e. Pathology Reports, MRI Reports, Operative Reports, etc.) submitted to the Coordinating Center should be de-identified. It is recommended that the assigned protocol case number be used for all participant specific documents. Participant initials may be included or retained for cross verification of identification.

3.5. Participant Registration

Refer to Section 4 of the protocol for the participant registration process.

3.6. Initiation of Therapy

Participants must be registered with the DF/HCC CTMS before the initiation of treatment or other protocol-specific interventions. Treatment and other protocol-specific interventions may not be initiated until the External Site receives confirmation of the participant's registration from the Coordinating Center. The DF/HCC Sponsor and IRB of record must be notified of any violations to this policy.

3.7. Eligibility Exceptions

No exceptions to the eligibility requirements for a protocol without IRB approval will be permitted. All External Sites are required to fully comply with this requirement. The process for requesting an eligibility exception is defined below.

3.8. Data Management

DF/HCC develops case report forms (CRF/eCRFs), for use with the protocol. These forms are designed to collect data for each study. DF/HCC provides a web-based training for all eCRF users.

3.8.1. Data Forms Review

Data submissions are monitored for timeliness and completeness of submission. If study forms are received with missing or questionable data, the submitting institution will receive a written or electronic query from the DF/HCC Office of Data Quality, Coordinating Center, or designee.

Responses to all queries should be completed and submitted within 14 calendar days.

If study forms are not submitted on schedule, the External Sites will receive a Missing Form Report from the Coordinating Center noting the missing forms. These reports are distributed by the Coordinating Center on a monthly basis.

3.9. Protocol Reporting Requirements

3.9.1. Protocol Deviations, Exceptions and Violations

Federal Regulations require an IRB to review proposed changes in a research activity to ensure that researchers do not initiate changes in approved research without IRB review and approval, except when necessary to eliminate apparent immediate hazards to the participant. DF/HCC requires all departures from the defined procedures set forth in the IRB approved protocol to be reported to the DF/HCC Sponsor and to the IRB of record.

3.9.2. Reporting Procedures

Requests to deviate from the protocol require approval from the IRB of record and the sponsor.

All protocol violations must be sent to the Coordinating Center in a timely manner. The Coordinating Center will provide training for the requirements for the reporting of violations.

3.9.3. Guidelines for Processing IND Safety Reports

The DF/HCC Sponsor will review all IND Safety Reports per DF/HCC requirements, and ensure that all IND Safety Reports are distributed to the External Sites as required by DF/HCC Policy. External Sites will review/submit to the IRB according to their institutional policies and procedures.

4. MONITORING: QUALITY CONTROL

The Coordinating Center, with the aid of the DF/HCC Office of Data Quality, provides quality control oversight for the protocol.

4.1. Ongoing Monitoring of Protocol Compliance

The External Sites may be required to submit participant source documents to the Coordinating Center for monitoring. External sites may also be subject to on-site monitoring conducted by the Coordinating Center.

The Coordinating Center will implement ongoing monitoring activities to ensure that External Sites are complying with regulatory and protocol requirements, data quality, and participant safety. Monitoring practices may include but are not limited to source data verification, and review and analysis of eligibility requirements, informed consent procedures, adverse events and all associated documentation, review of study drug administration/treatment, regulatory files, protocol departures reporting, pharmacy records, response assessments, and data management.

External Sites will be required to participate in Coordinating Center initiated teleconferences, as scheduled. The Coordinating Center will keep in close touch with the External Sites via email and phone.

Remote Monitoring: The Coordinating Center will request source documentation from Participating Institutions as needed to complete monitoring activities. Participating Institutions will be asked to forward de-identified copies of participants' medical record and source documents to the Coordinating Center to aid in source data verification.

4.2. Monitoring Reports

The DF/HCC Sponsor will review all monitoring reports to ensure protocol compliance. The DF/HCC Sponsor may increase the monitoring activities at External Sites that are unable to comply with the protocol, DF/HCC Sponsor requirements or federal and local regulations.

4.3. Accrual Monitoring

Prior to extending a protocol to an external site, the DF/HCC Sponsor will establish accrual requirements for each External Site. Accrual will be monitored for each External Site by the DF/HCC Sponsor or designee. Sites that are not meeting their accrual expectations may be subject to termination.

A minimum of 3 participants per site annually is recommended for Phase II trials.

5. AUDITING: QUALITY ASSURANCE

5.1. DF/HCC Internal Audits

All External Sites are subject to audit by the DF/HCC Office of Data Quality (ODQ). Typically, approximately 3-4 participants would be audited at the site over a 2-day period. If violations which impact participant safety or the integrity of the study are found, more participant records may be audited.

5.2. Audit Notifications

It is the External Site's responsibility to notify the Coordinating Center of all external audits or inspections (e.g., FDA, EMA, NCI) that involve this protocol. All institutions will forward a copy of final audit and/or re-audit reports and corrective action plans (if applicable) to the Coordinating Center, within 12 weeks after the audit date.

5.3. Audit Reports

The DF/HCC Sponsor will review all final audit reports and corrective action plans, if applicable. The Coordinating Center, must forward any reports to the DF/HCC ODQ per DF/HCC policy for review by the DF/HCC Audit Committee. For unacceptable audits, the

DF/HCC Audit Committee would forward the final audit report and corrective action plan to the IRB as applicable.

5.4. External Site Performance

The DF/HCC Sponsor and the IRB of record are charged with considering the totality of an institution's performance in considering institutional participation in the protocol.

External Sites that fail to meet the performance goals of accrual, submission of timely and accurate data, adherence to protocol requirements, and compliance with state and federal regulations, may be put on hold or closed.

APPENDIX C CTCAE VERSION 5.0 GRADING FOR SELECTED ADVERSE EVENTS

Common Terminology Criteria for Adverse Events (CTCAE), Version 5.0; National Cancer Institute; November 27, 2017; https://ctep.cancer.gov/protocolDevelopment/electronic_applications/docs/

**Adverse Event**	**Grade**				
	**1**	**2**	**3**	**4**	**5**
Pneumonitis	Asymptomatic; clinical or diagnositic observations only; intervention not indicated	Symptomatic; medical intervention indicated; limiting instrumental ADL	Severe symptoms; limiting self-care ADL; oxygen indicated	Life-threatening respiratory compromise; urgent intervention indicated (e.g., tracheotomy or intubation)	Death
Blurred vision	Intervention not indicated	Symptomatic; moderate decrease in visual acuity (best corrected visual acuity 20/40 and better or 3 lines or less decreased vision from known baseline); limiting instrumental ADL	Symptomatic with marked decrease in visual acuity (best corrected visual acuity worse than 20/40 or more than 3 lines of decreased vision from known baseline, up to 20/200); limiting self care ADL	Best corrected visual acuity of 20/200 or worse in the affected eye	-
Dry eye	Asymptomatic; clinical or diagnostic observations only; mild symptoms relieved by lubricants	Symptomatic; moderate decrease in visual acuity (best corrected visual acuity 20/40 and better or 3 lines or less decreased vision from known baseline)	Symptomatic with marked decrease in visual acuity (best corrected visual acuity worse than 20/40 or more than 3 lines of decreased vision from known baseline, up to 20/200); limiting self care ADL		-

**Adverse Event**	**Grade**				
	**1**	**2**	**3**	**4**	**5**
Keratitis	Asymptomatic; clinical or diagnostic observations only; intervention not indicated	Symptomatic; moderate decrease in visual acuity (best corrected visual acuity 20/40 and better or 3 lines or less decreased vision from known baseline)	Symptomatic with marked decrease in visual acuity (best corrected visual acuity worse than 20/40 or more than 3 lines of decreased vision from known baseline, up to 20/200); corneal ulcer; limiting self care ADL	Perforation or blindness (20/200 or worse) in the affected eye	-
Eye disorders – Other, specify	Asymptomatic or mild symptoms; clinical or diagnostic observations only; intervention not indicated	Moderate; minimal, local or noninvasive intervention indicated; limiting instrumental ADL; best corrected visual acuity 20/40 and better or 3 lines or less decreased vision from known baseline	Severe or medically significant but not immediately sight-threatening; limiting self care ADL; decrease in visual acuity (best corrected visual acuity worse than 20/40 or more than 3 lines of decreased vision from known baseline, up to 20/200)	Sight-threatening consequences; urgent intervention indicated; blindness (20/200 or worse) in the affected eye	-
Abbreviations: Activities of daily living (ADL)					

APPENDIX D SCHEMA FOR ADMINISTRATION OF STUDY-REQUIRED EYE DROPS

**Steroid eye drops –** 6 times daily **Lubricating eye drops** at least 15 minutes after steroid eye drops – daily as directed				**Steroid eye drops –** 4 times daily **Lubricating eye drops** at least 15 minutes after steroid eye drops – daily as directed				**Lubricating eye drops –** daily as directed												
←→				←→				←→												
1	2	3	4	5	6	7	8	9	10	11	12	13	14	15	16	17	18	19	20	21
Cycle Days																				

* Lubricating drops are continued if dose is held and/or cycle is delayed.
