# Supplementary material for: Mirvetuximab soravtansine plus pembrolizumab in recurrent folate receptor alpha-positive uterine serous carcinoma: a phase II trial
Source: Nat Commun. 2026 Mar 26;17:4449. doi: 10.1038/s41467-026-71102-x (PMC13183910; doi:10.1038/s41467-026-71102-x)
Supplement: Supplementary file 2 — Reporting Summary [file 41467_2026_71102_MOESM2_ESM.pdf]

nature portfolio

Corresponding author(s): Rebecca Porter, MD, PhD

Last updated by author(s): January 26. 2026

Reporting Summary

Nature Portfolio wishes to improve the reproducibility of the work that we publish. This form provides structure for consistency and transparency in reporting. For further information on Nature Portfolio policies, see our Editorial Policies and the Editorial Policy Checklist.

Statistics

For all statistical analyses, confirm that the following items are present in the figure legend, table legend, main text, or Methods section.

n/a	Confirmed	
☐	☒	The exact sample size (*n*) for each experimental group/condition, given as a discrete number and unit of measurement
☐	☒	A statement on whether measurements were taken from distinct samples or whether the same sample was measured repeatedly
☐	☒	The statistical test(s) used AND whether they are one- or two-sided<br>*Only common tests should be described solely by name; describe more complex techniques in the Methods section.*
☐	☒	A description of all covariates tested
☐	☒	A description of any assumptions or corrections, such as tests of normality and adjustment for multiple comparisons
☐	☒	A full description of the statistical parameters including central tendency (e.g. means) or other basic estimates (e.g. regression coefficient) AND variation (e.g. standard deviation) or associated estimates of uncertainty (e.g. confidence intervals)
☐	☒	For null hypothesis testing, the test statistic (e.g. *F*, *t*, *r*) with confidence intervals, effect sizes, degrees of freedom and *P* value noted<br>*Give P values as exact values whenever suitable.*
☒	☐	For Bayesian analysis, information on the choice of priors and Markov chain Monte Carlo settings
☒	☐	For hierarchical and complex designs, identification of the appropriate level for tests and full reporting of outcomes
☐	☒	Estimates of effect sizes (e.g. Cohen's *d*, Pearson's *r*), indicating how they were calculated

Our web collection on statistics for biologists contains articles on many of the points above.

Software and code

Policy information about availability of computer code

Data collection	OncoPanel : Garcia EP, Minkovsky A, Jia Y, Ducar MD, Shivdasani P, Gong X, Ligon AH, Sholl LM, Kuo FC, MacConaill LE, Lindeman NI, Dong F. Validation of OncoPanel: A Targeted Next- Generation Sequencing Assay for the Detection of Somatic Variants in Cancer. Arch Pathol Lab Med. 2017 Jun;141(6):751-758 . doi: 10.5858/arpa. 2016-05 27-OA. Epub 2017 Mar 3. PMID: 28557599.
Data analysis	Not applicable

For manuscripts utilizing custom algorithms or software that are central to the research but not yet described in published literature, software must be made available to editors and reviewers. We strongly encourage code deposition in a community repository (e.g. GitHub). See the Nature Portfolio guidelines for submitting code & software for further information.

Data

Policy information about availability of data

All manuscripts must include a data availability statement. This statement should provide the following information, where applicable:

- Accession codes, unique identifiers, or web links for publicly available datasets
- A description of any restrictions on data availability
- For clinical datasets or third party data, please ensure that the statement adheres to our policy

All 18 patients who initiated protocol therapy had targeted NGS testing performed previously as part of their clinical care (i.e., as standard of care) and data for these patients was retrieved directly from their clinical charts.

Research involving human participants, their data, or biological material

Policy information about studies with human participants or human data. See also policy information about sex, gender (identity/presentation), and sexual orientation and race, ethnicity and racism.

Reporting on sex and gender	This study enrolled patients of female gender only.
Reporting on race, ethnicity, or other socially relevant groupings	This study enrolled female patients with recurrent endometrial cancer irrespective of race, ethnicity or other socially relevant groupings. No analyses were made between races, ethnicities and socially relevant groups. Details of race and ethnicity are presented in the manuscript table.
Population characteristics	Eligible participants had pathologically confirmed EC that was recurrent or metastatic and/or resistant to standard therapies. Patients were required to have histologically confirmed MSS disease of serous histology to sign consent for pre-screening. Patients eligible fo rthe full study were required to have tumors with FOLR1 PS2+ expression of at least 50% or higher by central FOLR1 immunohistochemistry. Other eligibility criteria included measurable disease, 1-4 prior lines of therapies, ECOG performance status of ≤1, availability of archival formalin fixed paraffin embedded (FFPE) tissue and normal organ and marrow function. Initially, prior immune checkpoint inhibitor (ICI) therapy was excluded; an amendment during the study subsequently allowed patients to receive prior ICI. Key exclusion criteria included known active CNS disease, active or chronic corneal disorders, active autoimmune disease, history or pneumonitis or inflammatory lung disease (ILD), and history of allergy to monoclonal antibody therapy.
Recruitment	This was an investigator initiated study that was open at 3 institutions (Dana-Farber Cancer Institute, University of Massachusetts Memorial Medical Center and Northwell Heatlh). Of the 144 patients that were evaluated for FOLR1 IHC results via pre-screening, 45 patients met the positivity cut off for trial eligibility with ≥50% of cells exhibiting at least 2+ membrane staining intensity (PS2+). Of these patients, patients were eligible if they met the eligibility criteria of the study. Members of all races and ethnic groups were eligible for the trial. There was no bias in recruitment.
Ethics oversight	This trial was approved by the Dana-Farber Harvard Cancer Center IRB. All procedures involving human participants were carried out in accordance with the Declaration of Helsinki. Written informed consented was obtained from patients before enrollment to the study.

Note that full information on the approval of the study protocol must also be provided in the manuscript.

Field-specific reporting

Please select the one below that is the best fit for your research. If you are not sure, read the appropriate sections before making your selection.

- [x] Life sciences
- [ ] Behavioural & social sciences
- [ ] Ecological, evolutionary & environmental sciences

For a reference copy of the document with all sections, see nature.com/documents/nr-reporting-summary-flat.pdf

Life sciences study design

All studies must disclose on these points even when the disclosure is negative.

Sample size

Statistical considerations were developed for co-primary objectives to evaluate the objective response rate (ORR) by RECIST 1.1 and rate of progression-free survival at 6 months (PFS6), with a two-stage design that allows for early stopping for futility.
A two-stage test was constructed using the method of Sill, Rubinstein, Litwin and Yothers with the goal of stopping early for futility to limit patient exposure to an inactive agent while restricting the probabilities of type I and type II errors to approximately 10% and 15%, respectively. For the co-primary endpoints, a true ORR of 5% or less and a rate of progression-free survival at 6 months of (PFS6) 10% or less would not be of clinical interest (H0: πOR ≤ 5% AND π PFS6 ≤ 10%), whereas an improvement to a 20% objective response rate or 30% PFS6 rate would warrant further investigation of the study treatment. In the first stage, 16 patients were to be enrolled after which accrual was to be paused. If there were at least two objective responses or two patients progression-free at 6 months among the first 16 patients, the second stage of accrual would commence where an additional 19 patients would be enrolled. If at the end of the trial there were at least 4 treated patients with an objective response or 8 patients progression-free at 6 months, the study treatment would be considered worthy of further study. Specifically, analysis of historical data (GOG129 and GOG229 series) based on similar population of patients with endometrial cancer where the levels of activity were believed to be inactive to modestly active shows that an unacceptable rate for objective response (πOR) is 5% and for PFS6 (π PFS6) is 10%, respectively. A bivariate test of the co-primary endpoints was therefore constructed as follows:
H0: πOR ≤ 5% AND πPFS6 ≤ 10%
vs.
H1: πOR > 5% OR π PFS6 > 10%
Targeted alternative hypothesis: An absolute improvement in the rate of OR by 15% or PFS6 by 20% would be of clinical interest. Power was determined under two specific hypotheses (Hr: πOR = 20%, π PFS6 = 10%) and (Hs: πOR = 5%, π PFS6 = 30%) where sufficient activity is seen in only each endpoint, respectively. Sample size was selected to have sufficient power to reject the null under cases that assume either (a) independence or (b) dependence among the co-primary endpoints.

Data exclusions

All participants who had measurable disease at baseline, have received at least one cycle of therapy, and have had their disease re-evaluated will be considered evaluable for response. All participants will be evaluable for toxicity from the time of their first treatment on the study.

Replication

There were no attempts for replication due to the nature of the study. Specifically, each patient received one treatment (mirvetuximab/ pembrolizumab) and due to tumor tissue availability, biomarker assessment was performed once for each patient.

Randomization	This was a non-randomized study.
Blinding	There was no blinding.

Reporting for specific materials, systems and methods

We require information from authors about some types of materials, experimental systems and methods used in many studies. Here, indicate whether each material, system or method listed is relevant to your study. If you are not sure if a list item applies to your research, read the appropriate section before selecting a response.

Materials & experimental systems

n/a	Involved in the study
☐	☒ Antibodies
☒	☐ Eukaryotic cell lines
☒	☐ Palaeontology and archaeology
☒	☐ Animals and other organisms
☐	☒ Clinical data
☒	☐ Dual use research of concern
☒	☐ Plants

Methods

n/a	Involved in the study
☒	☐ ChIP-seq
☒	☐ Flow cytometry
☒	☐ MRI-based neuroimaging

Antibodies

Antibodies used	CD8: Leica, clone 4B11; cat # MCA1817T<br>PD-L1: Cell Signaling Technology, clone E1L3N; cat # 13684<br>FOXP3: Cell Signaling Technology, clone D2W8E; cat # 98377<br>PD-1, Abcam, clone EPR4877(2); cat # ab137132<br>CD163: Leica, clone 10D6; cat # 10D6<br>Cytokeratin: Dako, clone AE1/AE3; cat # IR05361-2<br>FOLR1: Abcam, clone EPR20277; cat # EPR20277
Validation	Validation of antibodies for ImmunoProfile analysis was performed as previously reported:<br>Lee EK, Konstantinopoulos PA. et al Phase 2, two-stage study of avelumab and axitinib in patients with mismatch repair proficient recurrent or persistent endometrial cancer. Gynecol Oncol. 2025 Jul;198:1-8. doi: 10.1016/j.ygyno.2025.05.006. Epub 2025 May 20. PMID: 40393272.

Clinical data

Policy information about clinical studies

All manuscripts should comply with the ICMJE guidelines for publication of clinical research and a completed CONSORT checklist must be included with all submissions.

Clinical trial registration	NCT03835819
Study protocol	The protocol is included as a supplement file in this submission.
Data collection	Patients were enrolled in 3 institutions: Dana-Farber Cancer Institute, University of Massachusetts Memorial Medical Center and Northwell Heatlh.
Outcomes	The primary objective was to assess the activity of mirvetuximab soravtansine and pembrolizumab in advanced or recurrent EC as determined by the frequency of patients who are alive and progression-free for a minimum of 6 months (PFS6) after initiating therapy, or who have objective tumor responses by RECIST 1.1 criteria. Secondary objectives included progression-free survival, overall survival, duration of response and the toxicity profile as classified using CTCAE v5.0.

Plants

Seed stocks	*Report on the source of all seed stocks or other plant material used. If applicable, state the seed stock centre and catalogue number. If plant specimens were collected from the field, describe the collection location, date and sampling procedures.*
Novel plant genotypes	*Describe the methods by which all novel plant genotypes were produced. This includes those generated by transgenic approaches, gene editing, chemical/radiation-based mutagenesis and hybridization. For transgenic lines, describe the transformation method, the number of independent lines analyzed and the generation upon which experiments were performed. For gene-edited lines, describe the editor used, the endogenous sequence targeted for editing, the targeting guide RNA sequence (if applicable) and how the editor was applied.*
Authentication	*Describe any authentication procedures for each seed stock used or novel genotype generated. Describe any experiments used to assess the effect of a mutation and, where applicable, how potential secondary effects (e.g. second site T-DNA insertions, mosiacism, off-target gene editing) were examined.*
